# Altered Expression and Localization of Tumor Suppressive E3 Ubiquitin Ligase SMURF2 in Human Prostate and Breast Cancer

**DOI:** 10.3390/cancers11040556

**Published:** 2019-04-18

**Authors:** Andrea Emanuelli, Dhanoop Manikoth Ayyathan, Praveen Koganti, Pooja Anil Shah, Liat Apel-Sarid, Biagio Paolini, Rajesh Detroja, Milana Frenkel-Morgenstern, Michael Blank

**Affiliations:** 1 Laboratory of Molecular and Cellular Cancer Biology, Azrieli Faculty of Medicine, Bar-Ilan University, 1311502 Safed, Israel; andrea.ema.2@gmail.com (A.E.); manikoth_dhanoop@yahoo.co.in (D.M.A.); kogantipraveen1@gmail.com (P.K.); poojaanil.98@gmail.com (P.A.S.); 2 Department of Pathology, The Galilee Medical Center, 22100 Nahariya, Israel; LiatA@gmc.gov.il; 3 Department of Pathology and Laboratory Medicine, Anatomic Pathology Unit 1, Fondazione IRCCS, Istituto Nazionale dei Tumori, 20133 Milan, Italy; Biagio.Paolini@istitutotumori.mi.it; 4 Laboratory of Cancer Genomics and BioComputing of Complex Diseases, Azrieli Faculty of Medicine, Bar-Ilan University, 1311502 Safed, Israel; rajesh007detroja@gmail.com (R.D.); milana.morgenstern@biu.ac.il (M.F.-M.)

**Keywords:** SMURF2, prostate and breast cancer, gene and protein expression, molecular localization, interactome

## Abstract

SMURF2, an E3 ubiquitin ligase and suggested tumor suppressor, operates in normal cells to prevent genomic instability and carcinogenesis. However, the mechanisms underlying SMURF2 inactivation in human malignancies remain elusive, as *SMURF2* is rarely found mutated or deleted in cancers. We hypothesized that SMURF2 might have a distinct molecular biodistribution in cancer versus normal cells and tissues. The expression and localization of SMURF2 were analyzed in 666 human normal and cancer tissues, with primary focus on prostate and breast tumors. These investigations were accompanied by *SMURF2* gene expression analyses, subcellular fractionation and biochemical studies, including SMURF2’s interactome analysis. We found that while in normal cells and tissues SMURF2 has a predominantly nuclear localization, in prostate and aggressive breast carcinomas SMURF2 shows a significantly increased cytoplasmic sequestration, associated with the disease progression. Mechanistic studies showed that the nuclear export machinery was not involved in cytoplasmic accumulation of SMURF2, while uncovered that its stability is markedly increased in the cytoplasmic compartment. Subsequent interactome analyses pointed to 14-3-3s as SMURF2 interactors, which could potentially affect its localization. These findings link the distorted expression of SMURF2 to human carcinogenesis and suggest the alterations in SMURF2 localization as a potential mechanism obliterating its tumor suppressor activities.

## 1. Introduction

Alterations in the expression, localization and functions of tumor suppressors and oncogenes are key hallmarks of cancer. In affected cells, these changes could trigger chromatin structural and epigenetic alterations, genomic instability, reprogram energy metabolism, and promote cell transformation, invasion and metastases. E3 ubiquitin ligases (E3s) are critical players in the regulation of protein homeostasis, localization and function both under physiological conditions and in disease states, particularly in cancer. Accumulating evidence shows that expression of E3s and their activities are often altered in human malignancies, marking these proteins as attractive therapeutic targets and disease biomarkers [1,2,3,4,5]. The evolutionarily conserved HECT-type E3 ubiquitin ligase SMURF2 emerges as one such factor. Recently, we and subsequently others showed that depletion of *Smurf2* in mouse tissues triggers a series of cascading events which under the stress of aging lead to carcinogenesis [6,7,8,9]. Mechanistically, we showed that SMURF2 regulates chromatin structure landscape and affects gene expression, DNA damage response and genomic integrity. Moreover, the data revealed that SMURF2 tumor suppressor activities were associated with and at least in part relied on its ability to regulate RNF20, another E3 ligase and chromatin modifier responsible for histone H2B mono-ubiquitination and, thereby, for the regulation of chromatin compaction, gene expression and DNA damage response [6,10,11,12,13]. Recently, we uncovered an additional mechanism by which SMURF2 could exert its tumor suppressor functions. We found that SMURF2 operates as a molecular editor of DNA topoisomerase IIα, protecting this enzyme from proteasomal degradation and preventing the formation of pathological chromatin bridges, a major cause of chromosomal translocations [14].

SMURF2 has also been shown to regulate stability, activity and/or localization of additional key cellular factors implicated in carcinogenesis and therapeutic responses. These factors include SMAD transducer/s [15] and the epidermal growth factor receptor (EGFR) [16], the Wnt/β-catenin signaling regulators GSK-3β [17] and AXIN [18], E3 ubiquitin ligases MDM2 [19] and SCFβTrCP [20], nuclear lamins (i.e., lamin A and its mutant form progerin) [21], histone methyltransferase EZH2 [22,23], transcription factors KLF5 [24], YY1 [25,26], ID1 [27], and special AT-rich sequence-binding protein-1 (SATB1) [28]. These and other studies established SMURF2 as an influential E3 ubiquitin ligase, whose activities are highly pertinent to human carcinogenesis. Interestingly, the evidence also showed that despite that SMURF2 operates in normal cells as a potent tumor suppressive barrier, in established tumors SMURF2 could switch a role and act as an oncogene rather than a tumor suppressor [9]. It has been hypothesized that the pleotropic substrate repertoire of SMURF2, including its interactors and targets which have not yet been identified, and the differential access of SMURF2 to its interactors in normal and cancer cells are related to its duality.

In this work, we systematically analyzed the expression levels and subcellular localization of SMURF2 in more than 660 human normal and cancer clinical samples, with primary focus on prostate and breast cancer. These tumors compose more than 25% of all human malignancies and are responsible for about 74,000 deaths annually in the US alone [29]. Cell fractionation, biochemical and mass spectrometry analyses designed to shed light on mechanisms underlying SMURF2’s alterations in cancer cells were also conducted in this study.

## 2. Results

### 2.1. Validation of the Specificity of Anti-SMURF2 Antibody in IHC Studies

**Figure 1:**
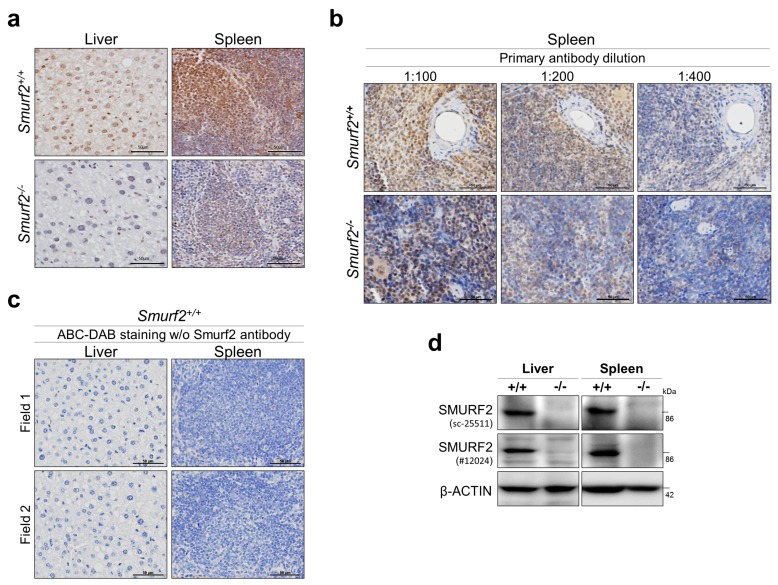
Examination of the specificity of SMURF2 antibody in IHC and immunoblots. (**a**) Representative IHC images of murine liver and spleen tissues, derived from *Smurf2*-deficient and wild-type control littermate mice, following their staining with anti-SMURF2 antibody (1:100, sc-25511; brown). The nuclei were counterstained with hematoxylin (blue). Scale bars: 50 μm. (**b**) IHC images of spleen samples stained with different dilutions of anti-SMURF2 antibody tested in (**a**). (**c**) ABC/DAB and hematoxylin-stainings without primary anti-SMURF2 antibody demonstrate the specificity of SMURF2 IHCs shown in panels (**a**,**b**). Scale bars: 50 μm. (**d**) Western blot analysis verifying *Smurf2* knock out in murine tissues.

One of the most critical aspects and a prerequisite for generation of reliable IHC data is validation of the specificity of reagents used for immunohistochemical staining, in particular of primary antibodies. To this end, we rigorously tested several anti-SMURF2 antibodies from a few different vendors using tissues derived from *Smurf2*^−/−^ mice. Murine and human SMURF2 share a very high homology, and are 99.2% identical in their amino acid composition [9]. The analyses revealed that one particular antibody (sc-25511, lot#F2414) shows a high specificity against endogenous Smurf2 in IHC staining (Figure 1). The antibody was raised against amino acids 204–253 of human SMURF2; the region which is highly conserved between human and mouse SMURF2 proteins. This antibody was subsequently used in all IHC examinations described in this study.

### 2.2. SMURF2 is Widely Expressed in Different Types of Human Normal Tissues

**Figure 2:**
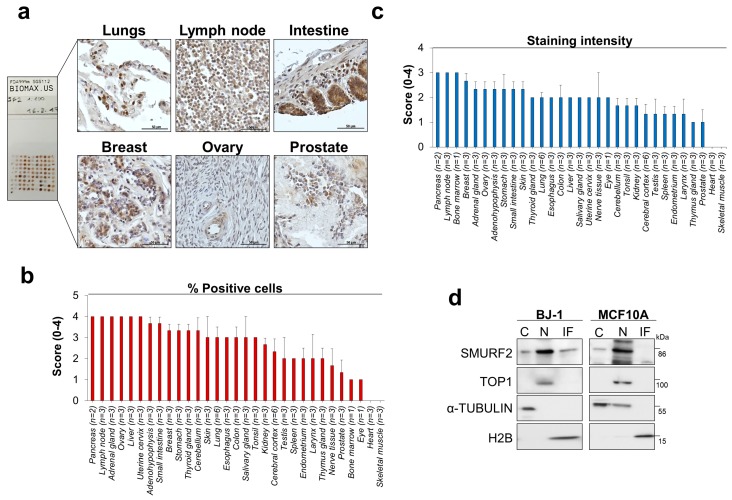
SMURF2 protein expression and biodistribution in human normal tissues and untransformed cells. (**a**) IHC analysis of SMURF2 expression in a panel of human normal tissues (FDA999m TMA). These tissues were sampled on the same slide and processed for IHC simultaneously. Scale bars: 50 μm. (**b**,**c**) Quantification of the percentage of SMURF2 positive cells and staining intensity in FDA999m TMA. The following scoring system was used: 0 ≤ 10%; 1 = 10–24%; 2 = 25–49%; 3 = 50–74%; 4 = 75–100%. Data are presented as mean ± SEM. (**d**) Western blot analysis of subcellular fractions, in which cytosolic (C), nucleoplasmic (N), and insoluble fractions (IF) were extracted from non-tumorigenic BJ1-hTERT and MCF10A cells, showing predominant nuclear localization of SMURF2 in these cell models. Protein loadings and degree of fractionations are demonstrated by membrane probing with antibodies specific against the nuclear-sequestered topoisomerase 1 (TOP1), cytosolic α-TUBULIN, and a chromatin component histone H2B.

Using the SMURF2-validated antibody, we analyzed the expression levels and biodistribution of SMURF2 protein in 32 types of human normal organs and tissues taken from three normal individuals (FDA999m TMA). Our IHC analyses revealed that SMURF2 is widely expressed in almost all human tissues, with the exception of heart and skeletal muscles, where SMURF2 levels were undetected (Figure 2a–c). Remarkably, in many tissues SMURF2 showed a prominent nuclear sequestration, although in some types of tissues (e.g., intestine) SMURF2 exhibited a mixed subcellular localization. Subsequent cell fractionation studies conducted on non-tumorigenic human mammary epithelial MCF10A cells and BJ1-hTERT dermal fibroblasts also showed nuclear sequestration of SMURF2 (Figure 2d).

The wide expression pattern of SMURF2 in human tissues was also evident from analyses of the *SMURF2* gene expression datasets. These analyses showed that *SMURF2* mRNA is ubiquitously expressed in all tested human organs/tissues (Figure S1a). Of note, gene expression analyses detected *SMURF2* mRNA also in skeletal muscles and heart, the organs in which SMURF2 protein levels were undetected by IHC. Additionally, the differential expression patterns of *SMURF2* mRNA and protein levels were monitored in testis, prostate, pancreas, lymph node and liver (Figure S1b).

### 2.3. Smurf2 Exhibits Altered Localization in Prostate Cancer

**Figure 3:**
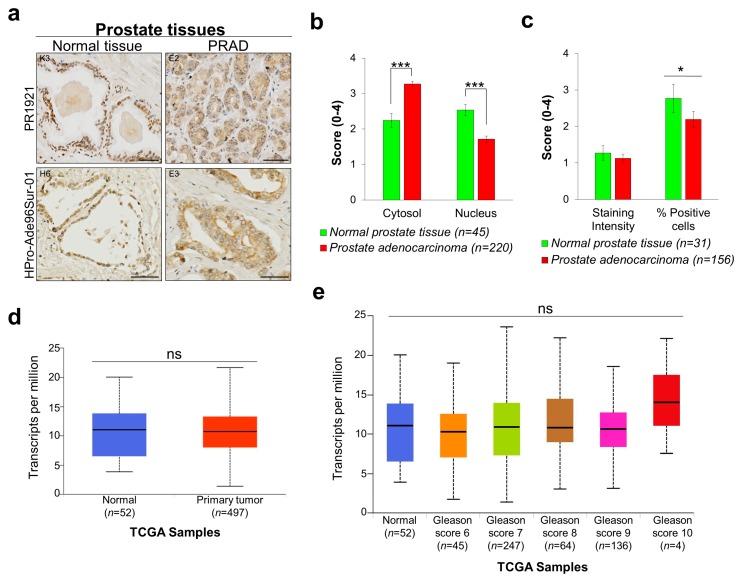
SMURF2 expression and aberrant localization in PRAD tissues. (**a**) Representative images of IHC staining of SMURF2 in PR1921 and HPro-Ade96Sur-01 TMAs containing prostate normal and carcinoma tissues. K3, E2, H6 and E3 are the coordinates of the samples in the tissue arrays. Scale bars: 50 μm. (**b**) The histological scores of SMURF2 subcellular localization in human prostate normal tissues and PRAD samples. Bars are Mean ± SEM. *** *p* < 0.001. (**c**) Quantification of SMURF2 staining intensity in PR1921 TMA. Bars are Mean ± SEM. * *p* < 0.05. (**d**,**e**) Boxplots showing relative expression of *SMURF2* in normal and PRAD clinical samples with different Gleason scores. ns—non-significant.

Next, we analyzed the expression levels and subcellular biodistribution of SMURF2 in two different sets of prostate TMAs: PR1921 and HPro-Ade96Sur-01, containing in total 287 human normal and prostate adenocarcinoma (PRAD) tissue samples. We found significant changes in SMURF2 protein localization in cancer versus normal tissues with elevated cytoplasmic and reduced nuclear levels of SMURF2 in cancer tissues (Figure 3a,b). Notably, the expression levels of SMURF2, quantified in IHCs as SMURF2 staining intensity, were comparable between normal and PRAD samples, though some reduction in the percentage of SMURF2-positive cells was recorded in cancer (Figure 3c). Similar to our IHC results, the *SMURF2* gene expression analysis conducted on prostate TCGA datasets showed no significant differences in mRNA expression of *SMURF2* between normal and PRAD tissues (Figure 3d,e). These findings stipulate that the main changes in the expression of SMURF2 in prostate cancer are related to its molecular localization.

### 2.4. Aberrant Expression of SMURF2 in Human Breast Carcinoma (BRCA) Tissues and Cell Strains

**Figure 4:**
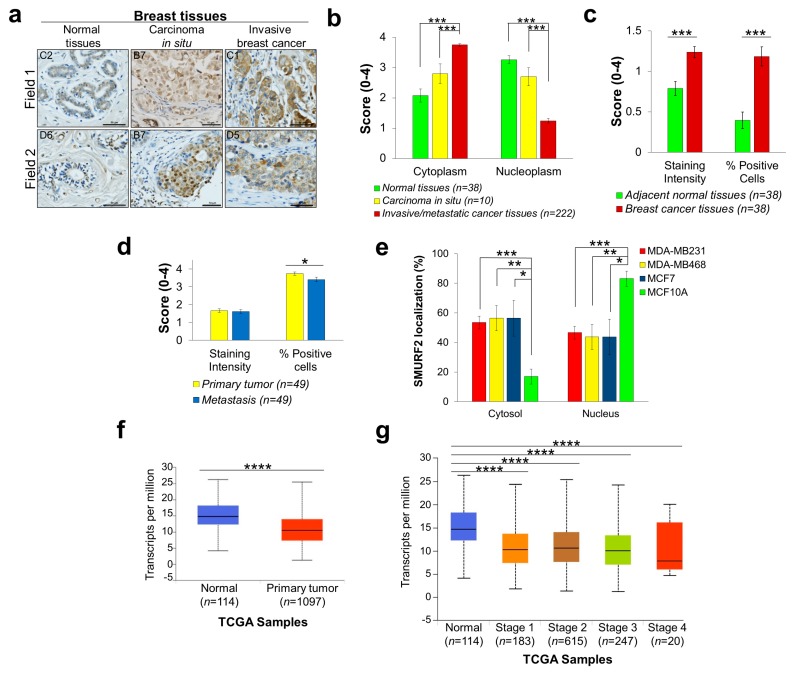
Altered expression and biodistribution of SMURF2 in human breast carcinoma tissues and cell strains. (**a**) Representative images of IHC staining of SMURF2 in normal breast tissues, carcinoma in situ and invasive breast cancer, C1, C2, B7 (field 2), D5 and D6 samples are coordinates in BR804a TMA; B7 (field 1) is from BR10011a TMA. Scale bars, 50 μm. (**b**) Quantification of the IHC data on SMURF2 localization in human normal and BRCA clinical samples obtained in three TMAs: BR804a, BR10011a and BR10010c. Bars represent Mean ± SEM. *** *p* < 0.001. (**c**) Quantification of SMURF2 staining intensity and percentage of SMURF2-positive cells in BR804a TMA, containing primary breast carcinoma and adjacent normal tissues. Bars are Mean ± SEM. *** *p* < 0.001. (**d**) Quantification of SMURF2 staining intensity and percentage of positive cells in BR10010c, which incorporates invasive and matching metastatic tissues. Bars are Mean ± SEM. * *p* < 0.05. (**e**) Quantification of the data on SMURF2 subcellular localization in untransformed and BRCA cells obtained from five independent experiments (western blot analyses). Bars represent Mean ± SEM. * *p* < 0.05; ** *p* < 0.01; *** *p* < 0.001. (**f**,**g**) Boxplots showing relative expression of *SMURF2* in normal and BRCA clinical samples. **** *p* < 0.0001.

To analyze the expression levels and subcellular localization of SMURF2 in breast tumors, we conducted IHC analyses on three different breast carcinoma TMAs: BR804a, composing primary breast carcinoma and adjacent normal tissues; BR10011a, consisting of invasive breast tumors, most of which are triple-negative (not expressing estrogen, progesterone and HER2/neu receptors); and BR10010c, incorporating invasive and matching metastatic tissues. Our analyses revealed that in contrast to normal and carcinoma in situ tissue samples, where SMURF2 exhibited a predominantly nuclear localization, in invasive and metastatic tumors SMURF2 was mostly sequestered in the cytoplasmic compartment of BRCA cells (Figure 4a,b). Remarkably, the staining intensity and percentage of SMURF2-positive cells were also considerably changed between BRCA and adjacent normal tissues, with significant elevation in BRCAs (Figure 4c). However, analysis of Smurf2 expression levels in primary versus matching metastatic tissues did not detect significant changes in SMURF2 staining intensity, although some reduction in the percentage of SMURF2-positive cells was monitored in metastatic tumors (Figure 4d).

To further corroborate our findings on altered SMURF2 biodistribution in BRCAs, we prepared the cytoplasmic and nuclear fractions from several breast carcinoma cell models, derived from patients’ metastatic sites: MDA-MB-231, MDA-MB-468 and MCF7 cells, and compared SMURF2 localization in these cells to mammary epithelial MCF10A cells. The data (Figure 4e and Figure S2a,b) show that despite the heterogeneous expression of SMURF2 in different BRCA strains, SMURF2 has a higher cytoplasmic/nuclear ratio in cancer cells in comparison to the non-tumorigenic MCF10A cell model, supporting our findings obtained in clinical tissue samples. Interestingly, although SMURF2 protein levels were significantly elevated in BRCA tumors, analysis of the TCGA RNA-Seq data showed a strong decrease in mRNA expression of *SMURF2* in cancer versus normal tissues (Figure 4f). This decrease was evident in all stages of the disease (Figure 4g).

### 2.5. SMURF2 Cytoplasmic Accumulation Does Not Involve the Nuclear Export Machinery

**Figure 5:**
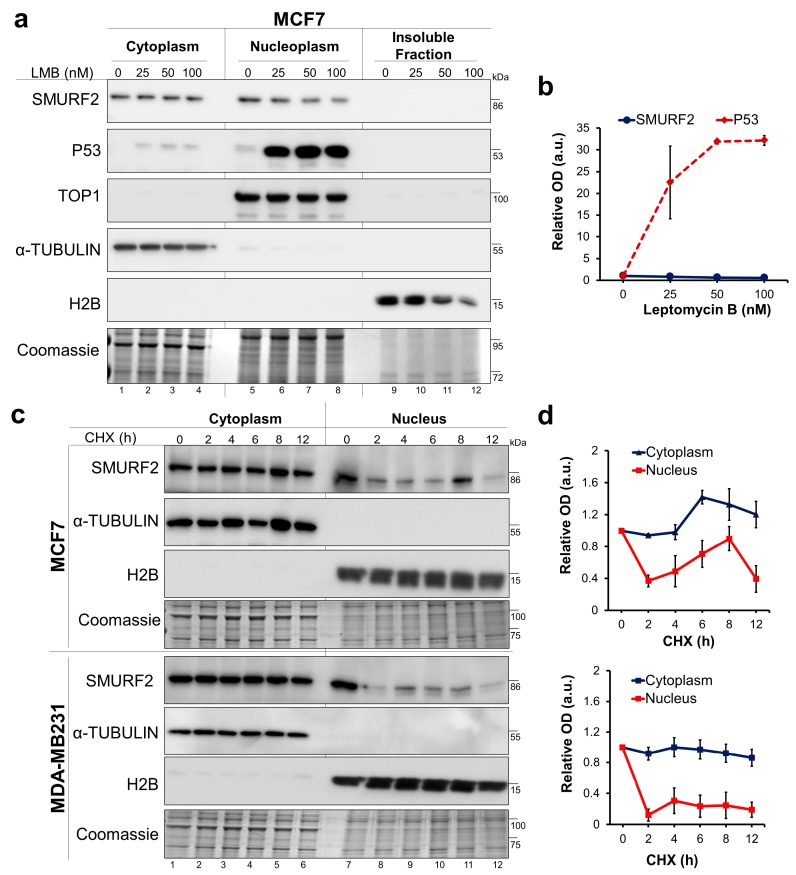
Mechanistic aspects associated with the distorted biodistribution of SMURF2 in cancer cells. (**a**) Western blot analysis of SMURF2 and p53 expression and localization in MCF7 cells following cell treatment with CRM1 inhibitor Leptomycin B (LMB; 4 h). α-TUBULIN, TOP1 and H2B were used as controls to validate the equal protein loading and quality of fractionation. Coomassie-gel staining was incorporated into the experiment as an additional control. (**b**) Quantification of the western blot analysis data of SMURF2 nuclear levels following cell treatment with LMB relative to nuclear loading control TOP1. Data are Mean ± SEM (*n* = 3). (**c**) Immunoblot analyses conducted on CHX-treated (50 μg/mL) and subsequently fractionated MCF7 and MDA-MB231 cells show that cytoplasmic and nuclear SMURF2 significantly differ in their protein stabilities. In these experiments, nuclear fractions contained both soluble and insoluble fractions solubilized with sonication. (**d**) Quantification of the western blot analysis data on SMURF2 turnover levels in the cytoplasmic and nuclear fractions of MCF7 (upper panel) and MDA-MB231 cells (bottom panel) obtained from three independent experiments. SMURF2 cytoplasmic and nuclear levels were quantified relative to the cytoplasmic expressed α-TUBULIN and nuclear histone H2B loading controls. Data are Mean ± SEM (*n* = 3).

We next asked whether the accumulation of SMURF2 in the cytoplasmic compartment of tumor cells is related to alterations in the nuclear export machinery, as such alterations were reported in different types of cancer, affecting protein localization and, consequently, tumor progression and chemoresistance [30,31]. CRM1 (Chromosomal Maintenance 1, also known as Exportin 1)-mediated nuclear export is a central mechanism regulating protein trafficking and translocations from the nucleus to the cytoplasm. Thus, if the cytoplasmic accumulation of SMURF2 depends on the CRM1-mediated nuclear export, its inhibition would increase the nuclear pool of SMURF2. To test this hypothesis, we treated breast carcinoma MCF7 cells with CRM1 inhibitor leptomycin B (LMB) and monitored the nucleo-cytoplasmic translocation of SMURF2 in these cells using western blot analysis. As a control, we measured effects of LMB treatment on localization of tumor suppressor p53, an established CRM1 substrate. The data (Figure 5a,b) show that LMB treatment efficiently inhibited the nucleo-cytoplasmic shuttling of p53, leading to its accumulation in the nucleus, but failed to do so on SMURF2, suggesting that accumulation of SMURF2 in the cytoplasm does not involve the CRM1-mediated nuclear export.

### 2.6. The Protein Stability of the Cytoplasmic and Nucelar Pools of SMURF2 is Vastly Different

Another possibility for elevated levels of SMURF2 in the cytoplasmic compartment of cancer cells is the differential protein stability of cytoplasmic versus nuclear pools of SMURF2. To examine this possibility, we treated MCF7 and MDA-MB-231 BRCA cells with the translation inhibitor cycloheximide (CHX) and monitored the kinetics of SMURF2 protein turnover in the cytoplasmic and nuclear fractions of these cells. Our findings revealed that the cytoplasm-sequestered SMURF2 is markedly more stable than its nuclear counterpart (Figure 5c,d), suggesting that SMURF2 accumulation in the cytoplasm is a result of its increased protein stability in this cellular compartment. Interestingly, a slower turnover rate of cytoplasmic SMURF2 was also observed in untransformed MCF10A cells (Figure S3), though a ratio between the cytoplasmic and nuclear pools of SMURF2 in these cells was considerably lower in comparison to tumor cells.

### 2.7. Characterization of the SMURF2 Interactome and Identification of 14-3-3 Isotypes as SMURF2 Novel Binding Partners Which Could Potentially Regulate SMURF2 Stability and Localization

The protein stability of SMURF2 could be significantly affected by its binding partners and substrates. The repertoire and abundance of these factors can be profoundly changed by the carcinogenic processes, affecting the ability of E3 ubiquitin ligase SMURF2 not only to control the expression of its substrates but also of itself. However, the lack of data on the SMURF2 interactome significantly impedes on the elucidation of mechanisms underlying SMURF2 stability regulation, localization and functions. To fill this gap, we performed two independent MS analyses of SMURF2 binding partners following its expression in human HEK293T cells. These cells were selected for analyses based on their exceptional ability to produce high yields of recombinant proteins (i.e., MYC-SMURF2). SMURF2 was expressed in these cells as either wild-type (WT) or E3 ligase-dead form (CG) (Figure S4a). Incorporation in the analyses of enzymatically inactive SMURF2 allowed us to access to proteins which otherwise might be invisible in the SMURF2 interactome due to their possible degradation by SMURF2. The expression levels of MYC-SMURF2WT and MYC-SMURF2CG and the efficiency of SMURF2 pull down from these cells were validated in immunoblots (Figure S4b). In addition, LC-MS/MS analyses verified the efficient pull-down of affinity-purified SMURF2 from these cells (Figure S4c). Following affinity purification and MS, the obtained SMURF2 interactome/s were annotated against human proteome database as described in the materials and methods and shown in Figure S4a.

**Figure 6:**
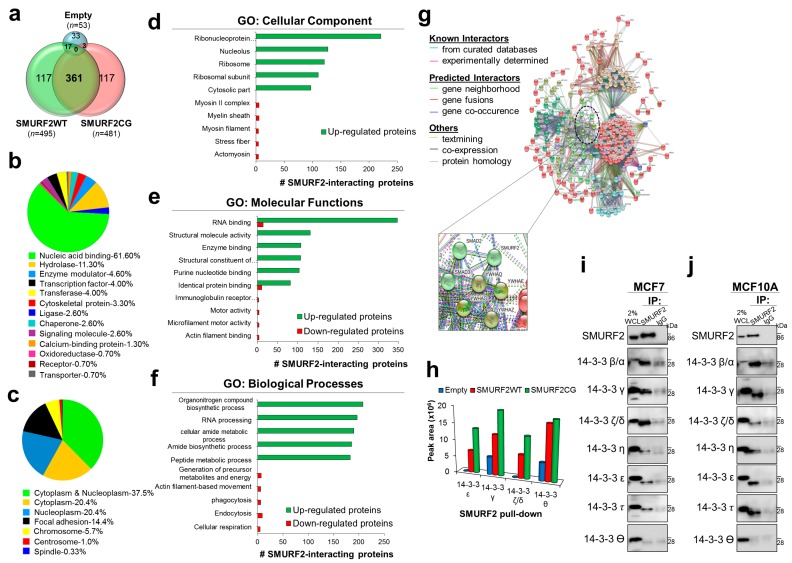
Molecular profiling and characterization of the SMURF2 interactome, and identification of 14-3-3 isoforms as Smurf2 binding partners. (**a**) Venn diagram showing the number of proteins identified in MS analyses as SMURF2WT and SMURF2CG interactors. Note, only 53 proteins were detected in empty control samples versus almost 500 unique proteins found in association with SMURF2. (**b**) The protein class of SMURF2 interacting proteins identified by PANTHER classification system. (**c**) The cellular localization of SMURF2 interactors investigated using the UniProtKB tool. (**d**–**f**) GO analysis of cellular components, molecular functions and biological processes enriched for SMURF2. Note, only top 10 enriched functions are shown. The detailed list is presented in Table S1. For statistical significance only q-values FDR Benjamini and Hochberg (B&H) ≤ 0.05 were considered. (**g**) Protein-protein interaction network of the SMURF2 interactome extracted from STRING 11.0 platform. Shown are only interactors that are connected within a network. Proteins are indicated by nodes labeled with the encoding gene symbol. Clusters identified by k-means clustering are shown in different colors: Red, ribosomal proteins; Brown, RNA biogenesis; Green, cytoskeletal proteins; Cyan, mitochondrial ribosomal proteins; Blue, elongation factors and ribosomal proteins; Olive, Purple and Light green, miscellaneous groups. Inset shows an enlargement of SMURF2 interaction network with 14-3-3 isoforms: YWHAE—epsilon (ε), YWHAQ—theta (θ), YWHAZ—Zeta/delta (ζ/δ) and YWHAG—gamma (γ). (**h**) The peak area of each particular isoform of 14-3-3 identified in LC-MS/MS for SMURF2WT, CG and empty Myc control samples. (**i**,**j**) Co-IP analyses of endogenous SMURF2 and different 14-3-3 isoforms showing the interactions between these proteins in both MCF7 and MCF10A cell models.

We found that the SMURF2 interactome consists of at least 495 proteins as its potential binding partners; 481 proteins passed the cut-off threshold as interactors of SMURF2CG. Substantial number of these proteins (*n* = 361) was overlapping between SMURF2WT and CG samples (Figure 6a). Further analysis with the PANTHER platform revealed that the SMURF2 interactome incorporates different classes of cellular proteins involved in nucleic acid bindings, enzymatic activities, cytoskeleton organization, molecular signaling, and other processes (Figure 6b). Based on the UniprotKB annotations, most of the identified SMURF2 binding partners were situated in the cytoplasm and nucleoplasm, although some of its interactors were also present in additional cellular compartments, including focal adhesion, chromosomes, centrosomes and spindles (Figure 6c). This distinct subcellular distribution of SMURF2 interactors was also evident in GO: cellular component analysis, which allowed us to further specify the localization of SMURF2 interactors—in ribonucleoprotein complexes, nucleolus, ribosomes, mitochondrion, cytoskeleton and cell junctions among others (Figure 6d).

GO enrichment analysis also pointed out the tight association of SMURF2 with critical molecular functions and biological processes governing cellular homeostasis and implicated in carcinogenesis. These include regulation of telomere organization and maintenance, epigenetic structure regulation, activities of DNA and RNA helicases, DNA binding and translational processes, activities of GTPases, protein kinases and phosphatases, metabolic and antioxidant activities, as well as the regulation of protein localization, cell cycle, cellular response to DNA damage and programmed cell death (Figure 6e,f and Table S1). The complexity of SMURF2 associations and its involvement in multiple molecular networks was further visualized with the STRING tool (Figure 6g). Remarkably, although SMURF2WT and CG interactomes shared some commonalities, the GO analyses revealed the differential involvement of these proteins in diverse molecular functions and biological processes, including structural and molecular activities, unfolded protein response, metabolic regulation, and others (Figure S5a–c and Table S2). These findings suggest that SMURF2 enzymatic activities could affect its molecular associations.

Intriguingly, the SMURF2 interactome analysis uncovered the 14-3-3 protein family members as SMURF2 novel interactors (Figure 6h). 14-3-3s (seven in humans) are abundant phospho-serine/phospho-threonine binding cytoplasmic proteins playing essential roles in cell signaling and cancer by regulation of protein localization, trafficking, and stability [32,33]. In addition, these proteins are prevalent in multiple human cancers, implying an intriguing possibility that SMURF2 stability and localization are coordinated by 14-3-3s.

To further verify the interactions between SMURF2 and 14-3-3 isotypes in cancerous and non-cancerous cell models, we conducted co-IP analyses in MCF7 and MCF10A cells, respectively, using the endogenously expressed SMURF2 and 14-3-3 proteins. The data show that in both cell lines SMURF2 interacts with six from seven 14-3-3 isoforms: β/α, γ, ζ/δ, η, ε and τ (Figure 6i,j). Subsequent cell fractionation studies corroborated the predominant localization of 14-3-3s in the cytoplasmic compartment of cancer cells (Figure S4d). Furthermore, our data supported the previous findings showing that expression of 14-3-3s is increased in tumor cells (Figure S4e).

## 3. Discussion

Dysregulated expression and altered localization of tumor suppressors and oncoproteins are notable features of human cancers. Indeed, several cancer-related proteins including p53, retinoblastoma (RB), sirtuin 1 (SIRT1), DNA topoisomerase IIα and breast cancer gene 1 (BRCA1) show profound alterations in subcellular localization in cancer versus normal cells and tissues [30]. This is particularly notable for tumor suppressors that normally reside and operate in the nucleus to keep the genomic integrity in check. The E3 ubiquitin ligase SMURF2 emerges as one such factor.

In this study, we show that SMURF2, a chromatin modifier and signal transduction regulator that operates in the nucleus of normal cells to prevent genomic instability, transformation and carcinogenesis, is severely altered in aggressive epithelial tumors. We demonstrated that in PRADs and invasive and metastatic BRCA tumors SMURF2 subcellular distribution is profoundly changed from nuclear to mostly cytoplasmic. In breast cancer this alteration in SMURF2 localization was also associated with the disease progression. In addition, our findings show that in primary BRCA tumors SMURF2 protein levels were considerably elevated in comparison to adjacent normal tissues, although no significant changes were detected in SMURF2 levels in primary versus matching metastatic tumors.

Interestingly, the *SMURF2* gene expression analysis revealed opposite to IHC results, and showed a significant decrease in *SMURF2* mRNA levels in BRCA samples in comparison to normal tissues. This finding implies a potential interference of post-translational mechanisms in SMURF2 regulation and suggests that monitoring of SMURF2 expression at protein level is advantageous over its gene expression analysis. In addition, the discrepancies between *SMURF2* mRNA and protein expression were also recorded in some types of human normal tissues, furthering the role of posttranslational factors in SMURF2 regulation.

Investigations of the mechanistic aspects associated with the distorted distribution of SMURF2 in human cancer showed that such an alteration does not involve the CRM1-mediated nuclear export. Further studies uncovered that SMURF2 protein stability is dramatically increased in the cytoplasm in comparison to the SMURF2’s nuclear pool, suggesting it as a mechanism underlying the cytoplasmic accumulation of SMURF2 in cancer. Interestingly, results obtained in CHX studies indicated that the cytoplasmic SMURF2 is considerably more stable than its nuclear counterpart in both cancerous and non-cancerous cell models, though the ratio between the cytoplasmic and nuclear pools of SMURF2 was higher in cancer cells. These data suggested that the increased abundance of SMURF2 in the cytoplasmic compartment of cancer cells is probably a secondary effect, and might be related to SMURF2 cellular interactions.

Subsequent investigations, incorporating the SMURF2 interactome analysis, revealed a conspicuous involvement of SMURF2 in diverse molecular processes pertinent to cell homeostasis maintenance, as well as to carcinogenesis and drug response/s. These analyses also pointed to the potential role of SMURF2’s enzymatic activities in its molecular associations and functions, suggesting that moderate structural alterations in SMURF2 could affect its binding preferences.

Furthermore, the stratification of the SMURF2 interactome exposed 14-3-3 proteins as its potential binding partners, which might be involved in SMURF2 regulation. Subsequent co-IP analyses conducted on endogenous proteins confirmed that SMURF2 complexes with six out of seven 14-3-3 isoforms in both cancerous and non-cancerous human cell models. 14-3-3s are abundant proteins, predominantly expressed in the cytoplasm, as was also observed in our study (Figure S4d). In addition, 14-3-3-s are often overexpressed in cancer cells [32], and our own data support this notion (Figure S4e). It is, therefore, plausible to assume that in cancer cells, where the abundance of 14-3-3s is increased, 14-3-3s sequester SMURF2 more efficiently, affecting SMURF2 protein turnover and leading to its accumulation in the cytoplasm.

Mechanistically, the decrease in the nuclear pool of SMURF2 and increase in its cytoplasmic abundance could change the SMURF2’s access to its protein substrates, which include both tumor suppressor and oncogenes. The decrease in the nuclear pool of SMURF2 would diminish its ability to negatively regulate the pro-tumorigenic factors residing in the nucleus (e.g., KLF5, YY1, ID1, SATB1 and others); while increased SMURF2 abundance in the cytoplasm would facilitate the cancer-promoting pathways, including EGFR-induced and KRAS-mediated signaling pathways and, suggestively, the WNT/β-CATENIN pathway (through the degradation of its negative regulators GSK-3β and AXIN) [9,16,17,18,20].

Additional influence on the accumulation of SMURF2 in the cytoplasm of cancer cells might be ascribed to the altered activities of protein kinases, in particular of serine/threonine kinases, which are known to facilitate the binding of 14-3-3 proteins to their substrates. Thus, in future studies it will be important to determine the impact of these factors on the ability of SMURF2 to complex with 14-3-3s. Remarkably, recent studies showed that SMURF2 could be phosphorylated by AKT [34] and ERK5 [35], two pro-tumorigenic serine/threonine kinases which expression and/or activities are upregulated in many cancers. Consequently, future studies focusing on the elucidation of interplay between expression and localization of SMURF2, 14-3-3 isoforms, and protein kinases and phosphatases associated with the SMURF2/14-3-3 axis regulation could provide the detailed mechanism on SMURF2 regulation in human normal and disease-affected tissues. These studies could also stipulate whether SMURF2 interacts with 14-3-3s directly or through the associated cellular factors shared by these proteins. All these efforts are needed to determine why and how SMURF2 loses its tumor suppressor functions and transforms to oncoprotein.

In summary, our findings together with the low mutation rate of *SMURF2* in human cancer (0.19% for prostate cancer and 0.48% for breast cancer [36]), suggest that the main alteration in the expression of SMURF2 in prostate and breast tumors is associated with its localization.

## 4. Materials and Methods

### 4.1. Human Tissue Microarrays, Mouse Tissues, Immunohistochemistry (IHC) and Ethics Statement

Three different tissue microarrays (TMAs) of breast cancer (BR804a, 80 samples; BR10011a, 100 samples; BR10010c, 100 samples), two TMAs of prostate cancer patients (PR1921, 192 samples; HPro-Ade96Sur-01, 95 samples) and TMA of human normal tissues (FDA999m, 99 samples) were purchased from a commercial source US Biomax, Inc (Rockville, MD, USA). These TMAs have accompanying histopathological information (available at www.biomax.us), including patient age, TNM, clinical stage, pathology grade and IHC markers (ER, PR and HER-2 for breast and PSA for prostate carcinomas). Prostate TMAs also contain Gleason score and grade annotations. All TMAs were of 5 µm thickness and have H&E and IHC quality controls, as indicated by the supplier. The patients’ identities are concealed and, therefore, untraceable.

*Smurf2* knock-out (*Smurf2*^−/−^) and littermate control wild-type (*Smurf2*^+/+^) C57BL/B6 mice were maintained at the faculty SPF animal facility and used for analyses under approved experimental IACUC protocol/s (Protocol 1: #29-05-2016; Approved on 3 May 2016; Protocol 2: #105-12-2017; Approved on 28 December 2017). Mice were genotyped as previously described [15]. Tissues derived from these mice were fixed in 4% formalin and 5 µm tissue sections were prepared. Antigen was retrieved using the heat-induced epitope retrieval (HIER) method in sodium citrate (10 mM, [pH 6.0]).

IHC stainings of mouse tissues and human TMAs were conducted on horizontally positioned slides using anti-SMURF2 antibody (sc-25511, lot#F2414; 1:100, Santa Cruz, Heidelberg, Germany) and enzymatic ABC-DAB staining (Vector Lab, Peterborough, UK). All IHC samples were counterstained with hematoxylin. All comparable tissue samples were positioned on the same slide and processed simultaneously. Histological images were taken using Axio Scan Z1 (20×/0.8 M27 objective; Zeiss, Jena, Germany).

TMA scorings were conducted by two board-certified pathologists at the Galilee Medical Center and at Istituto Nazionale dei Tumori. Based on the percentage of cells expressing SMURF2 either in cytosol or nucleus (or both), positive cells were scored for staining intensity and SMURF2 localization using the following system: 0 ≤ 10%; 1 = 10–24%; 2 = 25–49%; 3 = 50–74%; 4 = 75–100%, as previously described [6].

### 4.2. SMURF2 Gene Expression Analysis

The *SMURF2* gene expression analysis in human breast and prostate adenocarcinoma patient samples was performed using the TCGA datasets with the UALCAN web-server [37]. Transcripts per kilobase million (TPM) scores of the *SMURF2* gene expression were used to generate boxplots to assess the relative expression of *SMURF2*, and its significance, in breast and prostate cancer patients.

The data on the quantitative analysis of *SMURF2* mRNA expression levels in human normal organs and tissues were obtained from the Human Protein Atlas (HPA) (https://www.proteinatlas.org/ENSG00000108854-SMURF2/tissue), which included data from the HPA [38], the genome-based tissue expression (GTEx) [39], and the Fantom consortia [40].

### 4.3. Cell Cultures and Reagents

Cells used in this study were obtained from American Type Culture Collection (ATCC, Manassas, VA, USA). Cell authentication was performed at the Genomic Center of Biomedical Core Facility (Technion, Haifa, Israel). Human breast carcinoma MDA-MB-231, MDA-MB-468 and MCF7 cells as well as hTERT-immortalized normal human fibroblasts (BJ1-hTERT) and embryonic kidney HEK293T cells were cultured in Dulbecco’s Modified Eagle’s Medium containing 2mM L-Glutamine (DMEM, GIBCO, Paisley, UK). This medium was supplemented with 10% (*v*/*v*) fetal bovine serum (Biological Industries, Cromwell, CT, USA) and 1% (*v*/*v*) Pen-Strep (GIBCO). Spontaneously immortalized human mammary epithelial MCF10A cells were cultured in DMEM/F12 medium (Biological Industries) containing 2 mM L-glutamine, 5% donor horse serum (Sigma, St. Louis, MO, USA), 20 ng/mL epidermal growth factor (EGF; Peprotech, Rocky Hill, NJ, USA), 10 µg/mL insulin (Sigma), 0.5 µg/mL hydrocortisone (Sigma), 100 ng/mL cholera toxin (Sigma), and 1% (*v*/*v*) Pen-Strep (GIBCO). All cells were maintained in 5% CO2 at 37 °C. Leptomycin B and cycloheximide were obtained from Sigma.

### 4.4. Protein Extraction, Fractionation and Western Blot Analysis

To obtain whole cell lysates, cells were collected and re-suspended in RIPA buffer (50 mM Tris-HCl (pH 7.8), 1% NP40 substitute, 150 mM NaCl, 0.1% SDS, 0.5% p/v sodium deoxycholate) supplemented with freshly added protease and phosphatase inhibitors (Sigma). Samples were maintained on ice for 30 min and then sonicated for 1 min at 30% amplitude. Following sonication, the samples were centrifuged at 14,000 rpm for 10 min and supernatants were collected.

Cytoplasmic, nuclear and insoluble chromatin fractions were obtained as previously described [41]. In brief, cytoplasmic extracts were prepared by incubating cell pellets in hypotonic buffer A (10 mM Hepes (pH 7.9), 10 mM KCl, 1 mM EDTA, 1 mM EGTA, 1 mM DTT and 0.6% Nonidet P40 Substitute) for 15 min on ice. Nuclear extracts were then prepared by resuspension of cell nuclei in hypertonic buffer C (20 mM Hepes (pH 7.9), 400 mM KCl, 1 mM EDTA, 1 mM EGTA, 1 mM DTT) using vortex at 4 °C for 30 min. Chromatin fractions were then extracted from the previous step by sonication of residual nuclear pellet in buffer C (30% amplitude, 1 min on ice). All buffers were supplemented with protease and phosphatase inhibitors.

Protein concentrations were assessed using Pierce BCA protein assay kit (ThermoScientific, Waltham, MA, USA). Twenty micrograms of protein extracts from each sample were resolved in SDS-PAGE and detected in immunoblots using following antibodies: anti-SMURF2 (sc-25511, 1:1000, Santa Cruz, Dallas, TX, USA or #12024, 1:1500, Cell Signaling, Danver, MA, USA), anti-p53 (#2524, 1:1000, Cell Signaling), anti-PARP-1 (45D11, 1:5000, Cell Signaling), anti-TOP1 (#3552-1, 1:5000, Epitomics/Abcam, Cambridge, UK), histone-H2B (ab1790, 1:20000, Abcam Cambridge, UK), anti-β-ACTIN (#600401886, 1:3000, Rockland, Limerick, PA, USA) and anti-α-TUBULIN (T9026, 1:5000, Sigma). Horseradish peroxidase-conjugated secondary antibodies (Jackson ImmunoResearch Laboratories, West Grove, PA, USA) were used at the dilution of 1:10,000. Immunoblots were visualized using WesternBrightTM ECL (K-12045-D20, Advansta, San Jose, CA, USA) and documented using the Syngene G: BOX gel system (Syngene, Cambridge, UK). The expression of SMURF2 measured in immunoblots was quantified using Gel.Quant.NET (OmicX, Le-Petit-Quevilly, France) in the relation to appropriate loading controls.

### 4.5. Elucidation and Characterization of the SMURF2 Interactome

HEK293T cells were transfected with a full length MYC-tagged SMURF2 (either wild-type (WT) or its catalytically inactive Cys716Gly mutant (CG)) or an empty MYC vector as an additional control. Using these cells and recombinant SMURF2 proteins with an attached small tag allowed us both to produce sufficient protein yield for subsequent IP/MS analyses and to affinity-purify SMURF2 from cell extracts. Twenty-six hours post transfection, cells were lysed in 1% NP-40 buffer supplemented with protease and phosphatase inhibitors. Cell lysates were then pre-cleared with Protein-G Sepharose beads (4 Fast Flow, GE Healthcare, Chicago, IL, USA), and SMURF2 was pulled down using anti-MYC antibody (9E10, Santa Cruz), followed by incubation with Protein-G-Sepharose beads. The beads were then thoroughly washed in wash buffer (50 mM Tris (pH 8.5), 1 mM EGTA, 75 mM KCl) and eluted with 8 M urea buffer (8 M urea, 20 mM Tris (pH 7.5), 100 mM NaCl). The eluted samples were subjected to LC-MS/MS analysis at the Smoler Protein Research Center (Technion, Haifa, Israel), where proteins were digested with trypsin and analyzed in Q ExactiveTM Plus (ThermoScientific). The resulting peptides of the SMURF2 interactomes were annotated against human proteome database using the Proteome Discoverer™ software and two search algorithms—Sequest (ThermoScientific) and Mascot (Matrix Science, Boston, MA, USA). All identified peptides were filtered with high confidence (the false discovery rate (FDR) ≤ 1%), top rank, mass accuracy, and a minimum coverage of at least two peptides for each identified protein.

Subsequently, SMURF2 interactors were subjected to Gene Ontology (GO) analysis and classified using the ToppFun Suite bioinformatics tool [42]. The subcellular localization of identified SMURF2 interactors was also analyzed using UniProtKB [43]. Further protein classification was performed with the PANTHER platform [44]. In both UniProtKB and PANTHER-based analyses we used only proteins identified in the SMURF2 interactome with at least three different peptides.

For the re-construction of SMURF2 interaction network/s, we used STRING-11.0 tool with K-means clustering method applied for SMURF2 interactors (both WT and CG), which showed at least a two-fold increase in protein abundance and ≥ 3 peptides in coverage, in comparison to control. As a prediction method, we used a high confidence threshold (0.9) selected for text mining, experiments, databases, co-expression, neighborhood, gene fusion and co-occurrence, as described [45].

### 4.6. Co-Immunoprecipitation (co-IP)

For co-IP experiments, cells were lysed using 1% NP-40 buffer (1% NP-40 substitute, 25 mM Tris-HCl (pH 7.5), 137 mM NaCl, 1 mM EDTA, 1 mM EGTA, 5% glycerol, protease and phosphatase inhibitors). Lysates (500 μg of each protein sample) were incubated overnight at 4 °C with either anti-SMURF2 antibody (sc-25511; Santa Cruz) or rabbit IgG (I5006; Sigma) as a control. Protein G-Sepharose beads (4 Fast Flow; GE Healthcare) were then added, and samples were incubated for an additional 2 h at 4 °C under rotation. Subsequently, beads were washed four times with an ice-cold lysis buffer and boiled for 5 min in 5× SDS loading buffer (50 mM Tris-HCl (pH 8), 5 mM EDTA, 5% SDS, 50% glycerol, 50 mM DTT, 0.05% *w*/*v* bromophenol blue, 6% 2-mercaptoethanol). Immunoprecipitates were resolved in SDS-PAGE and probed with anti-14-3-3 family antibody kit (#9769, 1:1000, Cell Signaling), which incorporates antibodies specific for six different 14-3-3 isoforms, or with anti-14-3-3-θ-specific antibody (A303-146AT, 1:1000, Bethyl, Montgomery, TX, USA), which is not included in 14-3-3 family antibody kit. Immunoblots were then visualized and documented as described above.

### 4.7. Statistical Analyses

For TMA analyses, the Mann-Whitney *U* test was used to measure the differences in SMURF2 expression and localization between different groups. In all other experiments the data were analyzed using two-tail Student *t*-test. *p*-values < 0.05 were considered statistically significant (* *p* < 0.05; ** *p* < 0.01; *** *p* < 0.001; **** *p* < 0.0001).

## 5. Conclusions

Overall, our work illuminates the potential mechanism of how the tumor suppressive E3 ubiquitin ligase SMURF2 could be inactivated in human cancer through the alterations in its subcellular localization, and connects the altered biodistribution of SMURF2 to human carcinogenesis.

## Supplementary Materials

The following are available online at https://www.mdpi.com/2072-6694/11/4/556/s1, Figure S1: SMURF2 gene and protein expressions in human organs and tissues, Figure S2: The expression and molecular localization of SMURF2 in human breast cell models, Figure S3: Examination of SMURF2 turnover rate in the cytoplasmic and nuclear fraction of MCF10A cells, Figure S4: Experimental workflow and validation of SMURF2 affinity purification in immunoblots and MS analysis, and investigation of 14-3-3s in SMURF2 complexes and expression in breast cell models, Figure S5: Gene Ontology analyses of the SMURF2 interactomes for SMURF2WT and E3 ligase-dead SMURF2CG, Table S1: Details of GO terms for SMURF2, Table S2: Details of GO terms for SMURF2WT and SMURF2CG.

## References

[1] Popovic D., Vucic D., Dikic I. (2014). Ubiquitination in disease pathogenesis and treatment. Nat. Med..

[2] Rape M. (2018). Ubiquitylation at the crossroads of development and disease. Nat. Rev. Mol. Cell. Biol..

[3] Kumari N., Jaynes P.W., Saei A., Iyengar P.V., Richard J.L.C., Eichhorn P.J.A. (2017). The roles of ubiquitin modifying enzymes in neoplastic disease. Biochim. Biophys. Acta Rev. Cancer.

[4] Wang D., Ma L., Wang B., Liu J., Wei W. (2017). E3 ubiquitin ligases in cancer and implications for therapies. Cancer Metastasis Rev..

[5] Uchida C., Kitagawa M. (2016). RING-, HECT-, and RBR-type E3 Ubiquitin Ligases: Involvement in Human Cancer. Curr. Cancer Drug Targets.

[6] Blank M., Tang Y., Yamashita M., Burkett S.S., Cheng S.Y., Zhang Y.E. (2012). A tumor suppressor function of Smurf2 associated with controlling chromatin landscape and genome stability through RNF20. Nat. Med..

[7] Ramkumar C., Kong Y., Cui H., Hao S., Jones S.N., Gerstein R.M., Zhang H. (2012). Smurf2 regulates the senescence response and suppresses tumorigenesis in mice. Cancer Res..

[8] Zou X., Levy-Cohen G., Blank M. (2015). Molecular functions of NEDD4 E3 ubiquitin ligases in cancer. Biochim. Biophys. Acta Rev. Cancer.

[9] Koganti P., Levy-Cohen G., Blank M. (2018). Smurfs in Protein Homeostasis, Signaling, and Cancer. Front. Oncol..

[10] Fierz B., Chatterjee C., McGinty R.K., Bar-Dagan M., Raleigh D.P., Muir T.W. (2011). Histone H2B ubiquitylation disrupts local and higher-order chromatin compaction. Nat. Chem. Biol..

[11] Minsky N., Shema E., Field Y., Schuster M., Segal E., Oren M. (2008). Monoubiquitinated H2B is associated with the transcribed region of highly expressed genes in human cells. Nat. Cell Biol..

[12] Moyal L., Lerenthal Y., Gana-Weisz M., Mass G., So S., Wang S.Y., Eppink B., Chung Y.M., Shalev G., Shema E. (2011). Requirement of ATM-Dependent Monoubiquitylation of Histone H2B for Timely Repair of DNA Double-Strand Breaks. Mol. Cell.

[13] Tarcic O., Granit R.Z., Pateras I.S., Masury H., Maly B., Zwang Y., Yarden Y., Gorgoulis V.G., Pikarsky E., Ben-Porath I. (2017). RNF20 and histone H2B ubiquitylation exert opposing effects in Basal-Like versus luminal breast cancer. Cell Death Differ..

[14] Emanuelli A., Borroni A.P., Apel-Sarid L., Shah P.A., Manikoth Ayyathan D., Koganti P., Levy-Cohen G., Blank M. (2017). Smurf2-Mediated Stabilization of DNA Topoisomerase IIα Controls Genomic Integrity. Cancer Res..

[15] Tang L.Y., Yamashita M., Coussens N.P., Tang Y., Wang X., Li C., Deng C.X., Cheng S.Y., Zhang Y.E. (2011). Ablation of Smurf2 reveals an inhibition in TGF-β signalling through multiple mono-ubiquitination of Smad3. EMBO J..

[16] Ray D., Ahsan A., Helman A., Chen G., Hegde A., Gurjar S.R., Zhao L., Kiyokawa H., Beer D.G., Lawrence T.S. (2011). Regulation of EGFR protein stability by the HECT-type ubiquitin ligase SMURF2. Neoplasia.

[17] Wu Q., Huang J., Sampson E., Kim K.O., Zuscik M.J., O’Keefe R.J., Chen D., Rosier R.N. (2009). Smurf2 induces degradation of GSK-3β and upregulates β-catenin in chondrocytes: A potential mechanism for Smurf2-induced degeneration of articular cartilage. Exp. Cell Res..

[18] Kim S., Jho E. (2010). The Protein Stability of Axin, a Negative Regulator of Wnt Signaling, Is Regulated by Smad Ubiquitination Regulatory Factor 2 (Smurf2). J. Biol. Chem..

[19] Nie J., Xie P., Liu L., Xing G., Chang Z., Yin Y., Tian C., He F., Zhang L. (2010). Smad ubiquitylation regulatory factor 1/2 (Smurf1/2) promotes p53 degradation by stabilizing the E3 ligase MDM2. J. Biol. Chem..

[20] Shukla S., Allam U.S., Ahsan A., Chen G., Krishnamurthy P.M., Marsh K., Rumschlag M., Shankar S., Whitehead C., Schipper M. (2014). KRAS protein stability is regulated through SMURF2: UBCH5 complex-mediated β-TrCP1 degradation. Neoplasia.

[21] Borroni A.P., Emanuelli A., Shah P.A., Ilić N., Apel-Sarid L., Paolini B., Manikoth Ayyathan D., Koganti P., Levy-Cohen G., Blank M. (2018). Smurf2 regulates stability and the autophagic-lysosomal turnover of lamin A and its disease-associated form progerin. Aging Cell.

[22] Yu Y.L., Chou R.H., Shyu W.C., Hsieh S.C., Wu C.S., Chiang S.Y., Chang W.J., Chen J.N., Tseng Y.J., Lin Y.H. (2013). Smurf2-mediated degradation of EZH2 enhances neuron differentiation and improves functional recovery after ischaemic stroke. EMBO Mol. Med..

[23] Hu P., Nebreda A.R., Hanenberg H., Kinnebrew G.H., Ivan M., Yoder M.C., Filippi M.D., Broxmeyer H.E., Kapur R. (2018). P38α/JNK signaling restrains erythropoiesis by suppressing Ezh2-mediated epigenetic silencing of Bim. Nat Commun..

[24] Du J.X., Hagos E.G., Nandan M.O., Bialkowska A.B., Yu B., Yang V.W. (2011). The E3 ubiquitin ligase SMAD ubiquitination regulatory factor 2 negatively regulates Krüppel-like factor 5 protein. J. Biol. Chem..

[25] Ramkumar C., Cui H., Kong Y., Jones S.N., Gerstein R.M., Zhang H. (2013). Smurf2 suppresses B-cell proliferation and lymphomagenesis by mediating ubiquitination and degradation of YY1. Nat. Commun..

[26] Jeong H.M., Lee S.H., Yum J., Yeo C.Y., Lee K.Y. (2014). Smurf2 regulates the degradation of YY1. Biochim. Biophys. Acta.

[27] Kong Y., Cui H., Zhang H. (2011). Smurf2-mediated ubiquitination and degradation of Id1 regulates p16 expression during senescence. Aging Cell.

[28] Yu L., Dong L., Wang Y., Liu L., Long H., Li H., Li J., Yang X., Liu Z., Duan G. (2019). Reversible Regulation of SATB1 Ubiquitination by USP47 and SMURF2 Mediates Colon Cancer Cell Proliferation and Tumor Progression. Cancer Lett..

[29] Siegel R.L., Miller K.D., Jemal A. (2019). Cancer statistics, 2019. CA Cancer J. Clin..

[30] Wang X., Li S. (2014). Protein mislocalization: Mechanisms, functions and clinical applications in cancer. Biochim. Biophys. Acta.

[31] El-Tanani M., Dakir E.-H., Raynor B., Morgan R. (2016). Mechanisms of Nuclear Export in Cancer and Resistance to Chemotherapy. Cancers (Basel).

[32] Aghazadeh Y., Papadopoulos V. (2016). The role of the 14-3-3 protein family in health, disease, and drug development. Drug Discov. Today.

[33] Pennington K.L., Chan T.Y., Torres M.P., Andersen J.L. (2018). The dynamic and stress-adaptive signaling hub of 14-3-3: Emerging mechanisms of regulation and context-dependent protein-protein interactions. Oncogene.

[34] Choi Y.H., Kim Y.J., Jeong H.M., Jin Y.H., Yeo C.Y., Lee K.Y. (2014). Akt enhances Runx2 protein stability by regulating Smurf2 function during osteoblast differentiation. FEBS J..

[35] Iezaki T., Fukasawa K., Horie T., Park G., Robinson S., Nakaya M., Fujita H., Onishi Y., Ozaki K., Kanayama T. (2018). The MAPK Erk5 is necessary for proper skeletogenesis involving a Smurf-Smad-Sox9 molecular axis. Development.

[36] Tate J.G., Bamford S., Jubb H.C., Sondka Z., Beare D.M., Bindal N., Boutselakis H., Cole C.G., Creatore C., Dawson E. (2019). COSMIC: The Catalogue of Somatic Mutations In Cancer. Nucleic Acids Res..

[37] Chandrashekar D.S., Bashel B., Balasubramanya S.A.H., Creighton C.J., Rodriguez I.P., Chakravarthi B.V.S.K., Varambally S. (2017). UALCAN: A portal for facilitating tumor subgroup gene expression and survival analyses. Neoplasia.

[38] Uhlén M., Fagerberg L., Hallström B.M., Lindskog C., Oksvold P., Mardinoglu A., Sivertsson Å., Kampf C., Sjöstedt E., Asplund A. (2015). Proteomics. Tissue-based map of the human proteome. Science.

[39] Keen J.C., Moore H.M. (2015). The Genotype-Tissue Expression (GTEx) Project: Linking Clinical Data with Molecular Analysis to Advance Personalized Medicine. J. Pers. Med..

[40] Yu N.Y., Hallstrom B.M., Fagerberg L., Ponten F., Kawaji H., Carninci P., Forrest A.R., Fantom C.T., Hayashizaki Y., Uhlen M. (2015). Complementing tissue characterization by integrating transcriptome profiling from the Human Protein Atlas and from the FANTOM5 consortium. Nucleic Acids Res..

[41] Blank M., Lerenthal Y., Mittelman L., Shiloh Y. (2006). Condensin I recruitment and uneven chromatin condensation precede mitotic cell death in response to DNA damage. J. Cell Biol..

[42] Chen J., Bardes E.E., Aronow B.J., Jegga A.G. (2009). ToppGene Suite for gene list enrichment analysis and candidate gene prioritization. Nucleic Acids Res..

[43] UniProt Consortium (2015). UniProt: A hub for protein information. Nucleic Acids Res..

[44] Mi H., Poudel S., Muruganujan A., Casagrande J.T., Thomas P.D. (2016). PANTHER version 10: Expanded protein families and functions, and analysis tools. Nucleic Acids Res..

[45] Szklarczyk D., Morris J.H., Cook H., Kuhn M., Wyder S., Simonovic M., Santos A., Doncheva N.T., Roth A., Bork P. (2017). The STRING database in 2017: Quality-controlled protein-protein association networks, made broadly accessible. Nucleic Acids Res..

