# Supplementary material for: Altered Expression and Localization of Tumor Suppressive E3 Ubiquitin Ligase SMURF2 in Human Prostate and Breast Cancer
Source: Cancers (Basel). 2019 Apr 18;11(4):556. doi: 10.3390/cancers11040556 (PMC6521037; doi:10.3390/cancers11040556)
Supplement: Supplementary file 1 [file cancers-11-00556-s001.pdf]

Supplementary Materials For:

Altered expression and localization of tumor suppressive E3 ubiquitin ligase SMURF2 in human prostate and breast cancer

Andrea Emanuelli, Dhanoop Manikoth Ayyathan, Praveen Koganti, Pooja Anil Shah, Liat Apel-Sarid, Biagio Paolini, Rajesh Detroja, Milana Frenkel-Morgenstern and Michael Blank

a

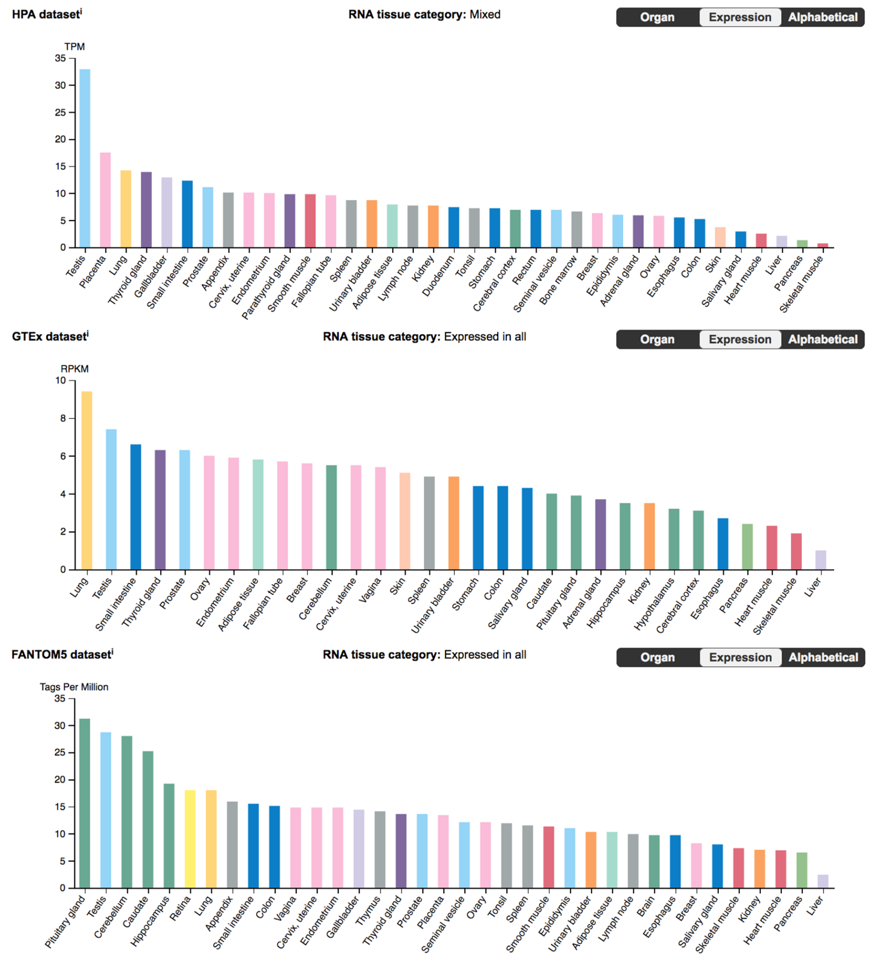

b

Organs	Datasets			IHC
	HPA	GTEx	Fantom5	
Testis	+++	+++	+++	+
Placenta	++	NA	++	NA
Lung	++	+++	++	++
Thyroid gland	++	++	++	++
Gall bladder	++	NA	++	NA
Small intestine	++	++	++	++
Prostate	++	++	++	+
Appendix	++	NA	++	NA
Cervix, Uterine	++	++	++	++
Endometrium	++	++	++	+
Parathyroid gland	++	NA	NA	NA
Smooth muscle	++	NA	++	NA
Fallopian tube	++	++	NA	NA
Spleen	++	++	++	+
Urinary bladder	++	++	++	NA
Adipose tissue	++	++	+	NA
Lymph node	++	NA	+	+++
Kidney	++	++	+	++
Duodenum	++	NA	NA	NA
Tonsil	++	NA	++	++
Stomach	++	++	NA	++
Cerebral cortex	++	++	NA	++
Rectum	++	NA	NA	NA
Seminal vesicle	++	NA	++	NA
Bone marrow	++	NA	NA	+++
Breast	++	++	++	++
Epididymis	++	NA	++	NA
Adrenal gland	++	++	NA	++
Ovary	++	++	++	++
Esophagus	++	+	++	++
Colon	++	++	++	++
Skin	+	++	NA	++
Salivary gland	+	++	+	++
Heart muscle	+	++	++	ND
Liver	+	+	+	++
Pancreas	++	+	+	+++
Skeletal muscle	+	++	++	ND
Cerebellum	NA	++	+++	++
Vagina	NA	++	++	NA
Caudate	NA	++	++	NA
Pituitary gland	NA	++	+++	NA
Hippocampus	NA	++	++	NA
Hypothalamus	NA	++	NA	NA
Retina	NA	NA	++	NA
Thymus	NA	NA	++	+
Brain	NA	NA	++	NA
Adenohypophysis	NA	NA	NA	++
Nerve tissue	NA	NA	NA	++
Eye	NA	NA	NA	++
Endometrium	NA	NA	NA	+
Larynx	NA	NA	NA	+

Low – + , Medium – ++ , High – +++ ,
NA – not available, ND – not detected

Figure S1. *SMURF2* gene and protein expressions in human organs and tissues. (**a**) The mRNA expression levels of SMURF2 in a panel of human normal organs and tissues: HPA, GTEx and FANTOM datasets. (**b**) Comparative analysis of IHC-based SMURF2 protein expression versus its gene expression.

a

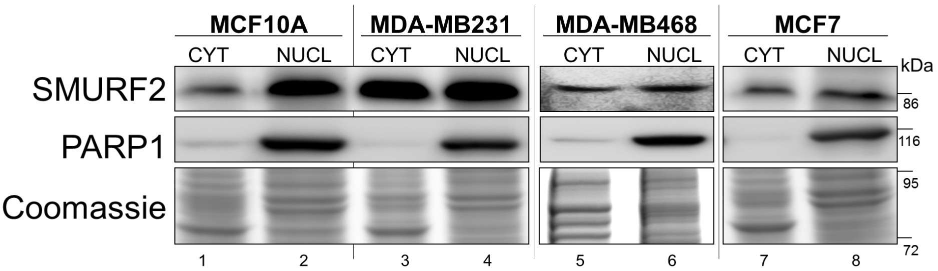

Cell line	Cytoplasmic/ nuclear ratio
MCF10A	0.29
MDA-MB231	1.24
MDA-MB468	0.73
MCF7	0.81

b

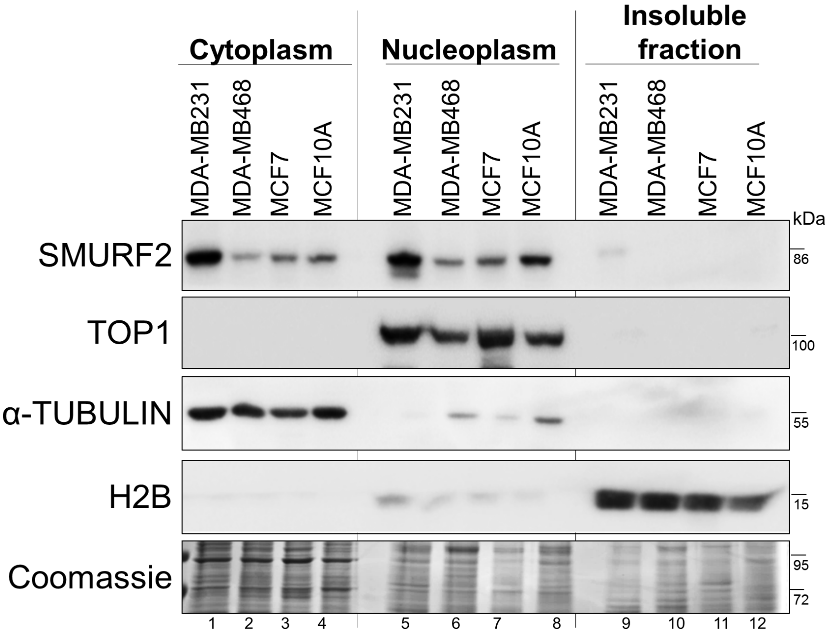

Cell line	Cytoplasmic/ nuclear ratio
MCF10A	0.55
MDA-MB231	1.01
MDA-MB468	0.62
MCF7	0.72

Figure S2. The expression and molecular localization of SMURF2 in human breast cell models. (**a**) Western blot analysis of SMURF2 expression in protein fractions prepared from non-tumorigenic mammary epithelial MCF10A cells and metastatic breast carcinoma cell models. Right panel shows the cytoplasm/nucleoplasm ratio of SMURF2 in the cytoplasmic (CYT) and nucleoplasmic (NUCL) compartments of the cells, normalized to coomassie staining. (**b**) SMURF2 expression analysis conducted as in (a), but incorporating three cellular fractions: cytoplasm, nucleoplasm and insoluble, chromatin-containing, fraction solubilized with sonication. All samples were run on the same SDS-PAGE and probed with the indicated antibodies. Coomassie gel staining was also conducted, and used for sample normalization. The quality of fractionation is demonstrated by sample probing with anti-TOP1 (nuclear marker), anti-α-TUBULIN (cytoplasmic marker), and anti-histone-H2B (chromatin marker) antibodies. Right panel shows the cytoplasm/nucleoplasm ratio of SMURF2 expression in untransformed and breast cancer cells normalized to coomassie.

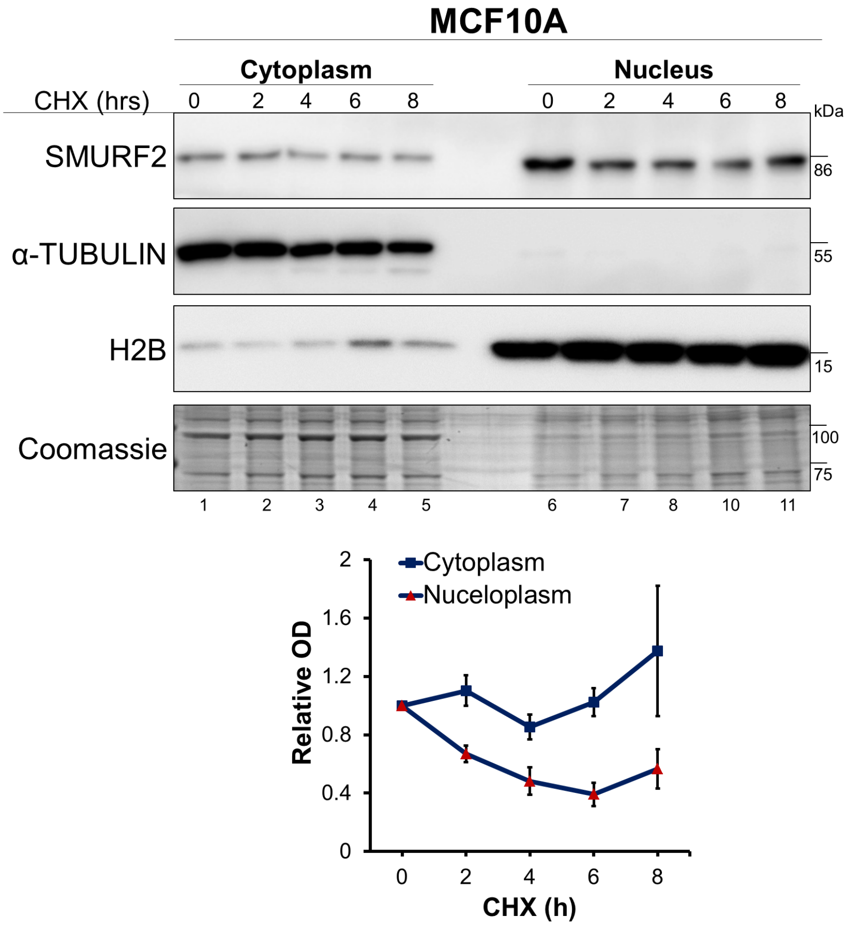

Figure S3. Examination of SMURF2 turnover rate in the cytoplasmic and nuclear fraction of MCF10A cells. Cells were treated with CHX and fractionated as described in Figure 5. The bottom panel shows the quantification of the data relative to the appropriate loading controls (α-TUBULIN for cytoplasmic fraction and H2B for nuclear fraction) derived from two independent experiments. The data are presented as Mean±SEM. The sample loading was also visualized with coomassie gel staining.

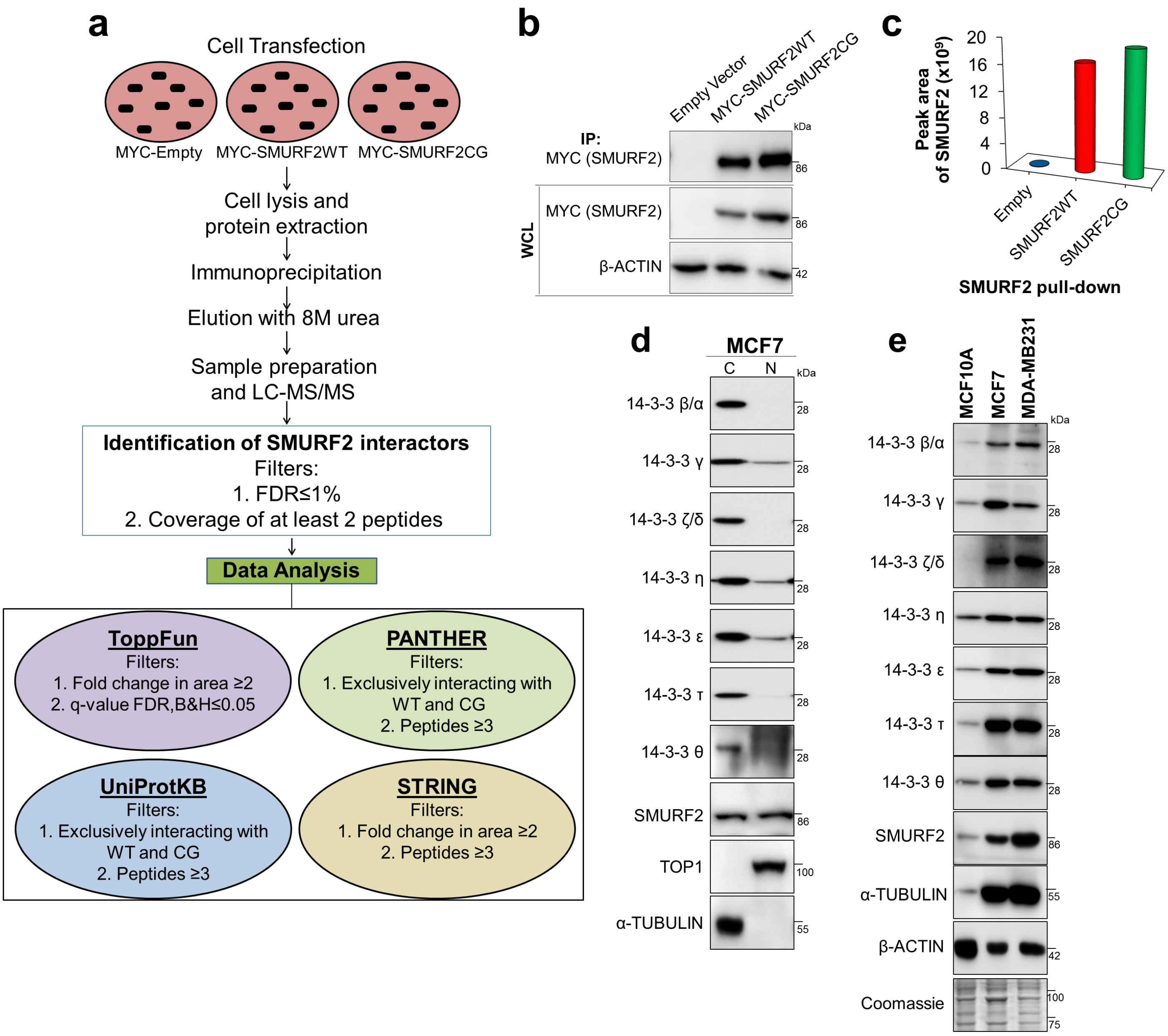

Figure S4. Experimental workflow and validation of SMURF2 affinity purification in immunoblots and MS analysis, and investigation of 14-3-3s in SMURF2 complexes and expression in breast cell models.

(**a**) Schematic diagram of experimental design used for identification and characterization of the SMURF2 interactome. (**b**) Western blot analysis showing the efficiency of SMURF2 pull-down from HEK293T cells. (**c**) LC-MS/MS analysis showing the efficiency of SMURF2 pull-down from SMURF2WT and SMURF2CG expressing cells. Empty vector was used as control. Data are presented as peak area of SMURF2.

(**d**) Subcellular localization of 14-3-3 isoforms in MCF7 cells. The quality of fractionation is demonstrated by membrane probing with cytoplasmic marker α-TUBULIN, and nuclear protein marker TOP1. (**e**) Comparative western blot analysis of the expression levels of 14-3-3s in untransformed and breast cancer cells. Equal amount of proteins (20 µg per lane) were resolved in SDS-PAGE and analysed in immunoblots with the indicated antibodies. Because these different cell strains differ in the expression levels of housekeeping genes (*e.g.* α–TUBULIN and β–ACTIN), these samples were also subjected to coomassie staining.

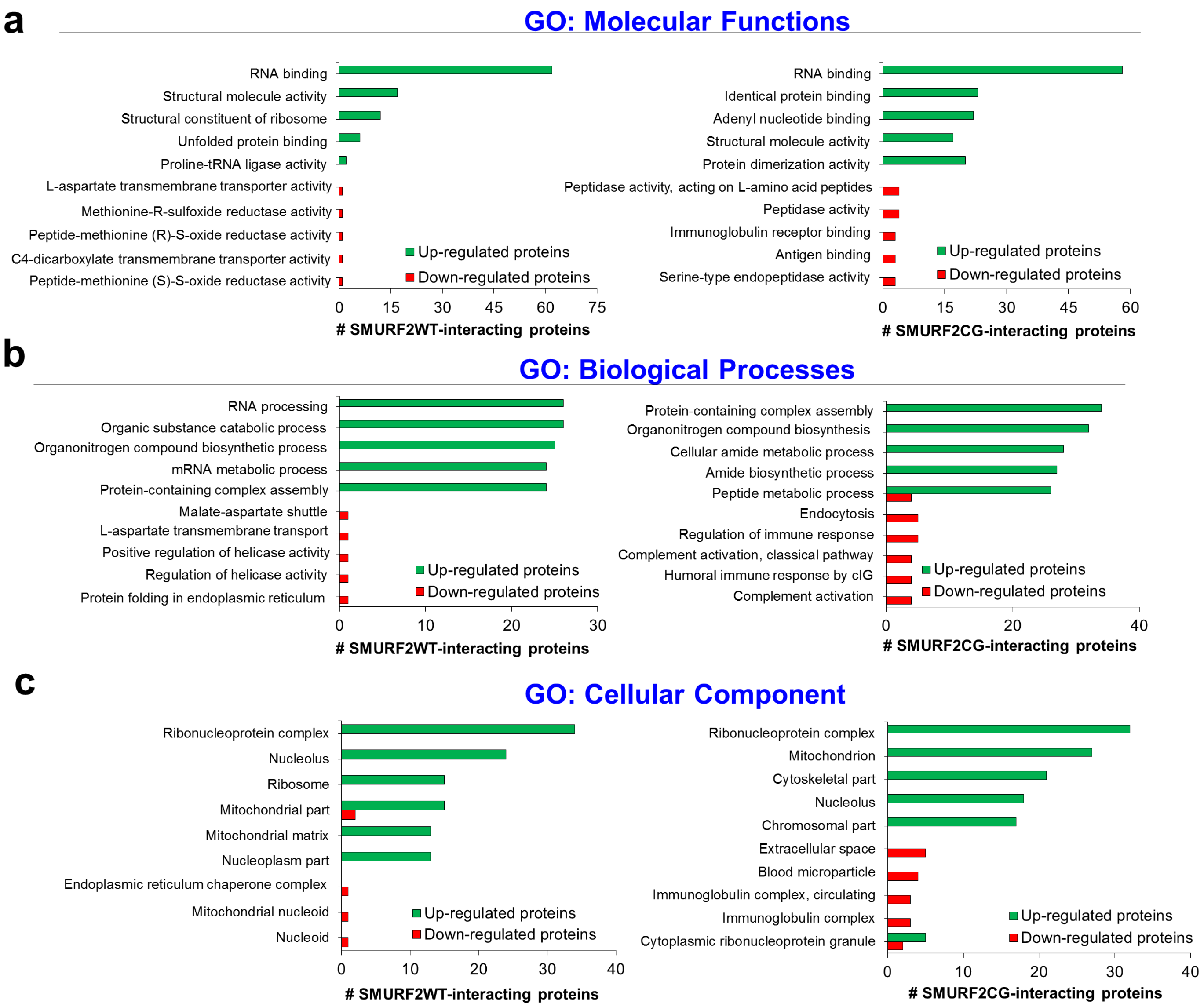

Figure S5. Gene Ontology analyses of the SMURF2 interactomes for SMURF2WT and E3 ligase-dead SMURF2CG. (**a-c**), GO terms for molecular functions, biological processes and cellular components enriched in SMURF2WT and SMURF2CG samples. For statistical significance, only q-values FDR B&H ≤0.05 were considered. cIG- circulating immunoglobulin. Note, only top ten enriched functions are shown. The full list is presented in Table S2

Table S1: **Details of GO terms for SMURF2**

Increased in the presence of SMURF2 (wild-type and mutant Cys716Gly form)				
Category	ID	Name	q-value FDR B&H	Hit Count in Query List
**Molecular Functions**				
GO: Molecular Function	GO:0003723	RNA binding	2.22E-217	347
GO: Molecular Function	GO:0005198	structural molecule activity	4.784E-58	131
GO: Molecular Function	GO:0019899	enzyme binding	1.034E-07	108
GO: Molecular Function	GO:0003735	structural constituent of ribosome	1.429E-100	107
GO: Molecular Function	GO:0017076	purine nucleotide binding	0.000000341	104
GO: Molecular Function	GO:0035639	purine ribonucleoside triphosphate binding	7.577E-07	100
GO: Molecular Function	GO:0032550	purine ribonucleoside binding	0.000001041	100
GO: Molecular Function	GO:0032549	ribonucleoside binding	0.000001044	100
GO: Molecular Function	GO:0001883	purine nucleoside binding	0.000001044	100
GO: Molecular Function	GO:0001882	nucleoside binding	0.000001217	100
GO: Molecular Function	GO:0032555	purine ribonucleotide binding	0.000002354	100
GO: Molecular Function	GO:0032553	ribonucleotide binding	0.000003451	100
GO: Molecular Function	GO:0044877	protein-containing complex binding	1.294E-07	95
GO: Molecular Function	GO:0030554	adenyl nucleotide binding	0.0000198	83
GO: Molecular Function	GO:0042802	identical protein binding	3.981E-07	82
GO: Molecular Function	GO:0005524	ATP binding	0.00002856	80
GO: Molecular Function	GO:0032559	adenyl ribonucleotide binding	0.00007844	80
GO: Molecular Function	GO:0016462	pyrophosphatase activity	1.88E-08	62
GO: Molecular Function	GO:0016818	hydrolase activity, acting on acid anhydrides, in phosphorus-containing anhydrides	1.88E-08	62
GO: Molecular Function	GO:0016817	hydrolase activity, acting on acid anhydrides	1.88E-08	62
GO: Molecular Function	GO:0003729	mRNA binding	7.706E-43	60
GO: Molecular Function	GO:0017111	nucleoside-triphosphatase activity	9.789E-08	58
GO: Molecular Function	GO:0019900	kinase binding	0.001287	41
GO: Molecular Function	GO:0042803	protein homodimerization activity	0.0382	40
GO: Molecular Function	GO:0019901	protein kinase binding	0.001219	38

GO: Molecular Function	GO:0016887	ATPase activity	0.00002187	35
GO: Molecular Function	GO:0019843	rRNA binding	3.726E-31	34
GO: Molecular Function	GO:0003682	chromatin binding	0.005641	31
GO: Molecular Function	GO:0008134	transcription factor binding	0.03041	31
GO: Molecular Function	GO:0042623	ATPase activity, coupled	0.0003794	26
GO: Molecular Function	GO:0019001	guanyl nucleotide binding	0.005549	26
GO: Molecular Function	GO:0032561	guanyl ribonucleotide binding	0.01121	25
GO: Molecular Function	GO:0051082	unfolded protein binding	1.465E-12	24
GO: Molecular Function	GO:0005525	GTP binding	0.02131	23
GO: Molecular Function	GO:0043021	ribonucleoprotein complex binding	2.518E-09	22
GO: Molecular Function	GO:0003725	double-stranded RNA binding	1.657E-13	21
GO: Molecular Function	GO:0003924	GTPase activity	0.0003711	21
GO: Molecular Function	GO:0031625	ubiquitin protein ligase binding	0.01222	19
GO: Molecular Function	GO:0044389	ubiquitin-like protein ligase binding	0.01418	19
GO: Molecular Function	GO:0003730	mRNA 3'-UTR binding	2.403E-11	17
GO: Molecular Function	GO:0003727	single-stranded RNA binding	3.443E-08	17
GO: Molecular Function	GO:0004386	helicase activity	0.0005742	17
GO: Molecular Function	GO:0070035	purine NTP-dependent helicase activity	0.00001442	16
GO: Molecular Function	GO:0008026	ATP-dependent helicase activity	0.00001442	16
GO: Molecular Function	GO:0003697	single-stranded DNA binding	0.00002451	15
GO: Molecular Function	GO:0005200	structural constituent of cytoskeleton	0.00004594	15
GO: Molecular Function	GO:0001047	core promoter binding	0.008752	14
GO: Molecular Function	GO:0019902	phosphatase binding	0.02478	14
GO: Molecular Function	GO:0008135	translation factor activity, RNA binding	0.0001053	13
GO: Molecular Function	GO:0008186	RNA-dependent ATPase activity	0.00002451	12
GO: Molecular Function	GO:0004004	ATP-dependent RNA helicase activity	0.00002451	12
GO: Molecular Function	GO:0003724	RNA helicase activity	0.00008453	12
GO: Molecular Function	GO:0046332	SMAD binding	0.0001053	12
GO: Molecular Function	GO:0031072	heat shock protein binding	0.001946	12
GO: Molecular Function	GO:0042826	histone deacetylase binding	0.003781	12

GO: Molecular Function	GO:0031490	chromatin DNA binding	0.001182	11
GO: Molecular Function	GO:0036002	pre-mRNA binding	1.169E-07	10
GO: Molecular Function	GO:0035326	enhancer binding	0.0194	10
GO: Molecular Function	GO:0047485	protein N-terminus binding	0.03401	10
GO: Molecular Function	GO:0045182	translation regulator activity	0.000108	9
GO: Molecular Function	GO:0000049	tRNA binding	0.0003711	9
GO: Molecular Function	GO:0016209	antioxidant activity	0.007895	9
GO: Molecular Function	GO:0070180	large ribosomal subunit rRNA binding	4.124E-09	8
GO: Molecular Function	GO:0003746	translation elongation factor activity	0.000004572	8
GO: Molecular Function	GO:0070717	poly-purine tract binding	0.00001976	8
GO: Molecular Function	GO:0004722	protein serine/threonine phosphatase activity	0.01418	8
GO: Molecular Function	GO:0002039	p53 binding	0.0194	8
GO: Molecular Function	GO:0048027	mRNA 5'-UTR binding	4.187E-07	7
GO: Molecular Function	GO:0008187	poly-pyrimidine tract binding	0.00007997	7
GO: Molecular Function	GO:0042162	telomeric DNA binding	0.0007069	7
GO: Molecular Function	GO:0004812	aminoacyl-tRNA ligase activity	0.005549	7
GO: Molecular Function	GO:0016875	ligase activity, forming carbon-oxygen bonds	0.005549	7
GO: Molecular Function	GO:0043022	ribosome binding	0.01561	7
GO: Molecular Function	GO:0003678	DNA helicase activity	0.03642	7
GO: Molecular Function	GO:0000979	RNA polymerase II core promoter sequence-specific DNA binding	0.03805	7
GO: Molecular Function	GO:0008097	5S rRNA binding	0.000000341	6
GO: Molecular Function	GO:0008143	poly(A) binding	0.0001805	6
GO: Molecular Function	GO:0008266	poly(U) RNA binding	0.0002536	6
GO: Molecular Function	GO:0017091	AU-rich element binding	0.001048	6
GO: Molecular Function	GO:0043531	ADP binding	0.01761	6
GO: Molecular Function	GO:0016684	oxidoreductase activity, acting on peroxide as acceptor	0.03041	6
GO: Molecular Function	GO:0004003	ATP-dependent DNA helicase activity	0.03041	6
GO: Molecular Function	GO:0030331	estrogen receptor binding	0.03041	6
GO: Molecular Function	GO:0051920	peroxiredoxin activity	0.00004049	5
GO: Molecular Function	GO:0032552	deoxyribonucleotide binding	0.00004049	5

GO: Molecular Function	GO:0005072	transforming growth factor beta receptor, cytoplasmic mediator activity	0.0001418	5
GO: Molecular Function	GO:0019103	pyrimidine nucleotide binding	0.0002434	5
GO: Molecular Function	GO:0060590	ATPase regulator activity	0.02878	5
GO: Molecular Function	GO:0030544	Hsp70 protein binding	0.03465	5
GO: Molecular Function	GO:0051059	NF-kappaB binding	0.03764	5
GO: Molecular Function	GO:0016814	hydrolase activity, acting on carbon-nitrogen (but not peptide) bonds, in cyclic amidines	0.03764	5
GO: Molecular Function	GO:0017069	snRNA binding	0.03764	5
GO: Molecular Function	GO:0004532	exoribonuclease activity	0.0415	5
GO: Molecular Function	GO:0030618	transforming growth factor beta receptor, pathway-specific cytoplasmic mediator activity	0.0001053	4
GO: Molecular Function	GO:0050733	RS domain binding	0.0005742	4
GO: Molecular Function	GO:0070181	small ribosomal subunit rRNA binding	0.001048	4
GO: Molecular Function	GO:0008494	translation activator activity	0.002685	4
GO: Molecular Function	GO:0046933	proton-transporting ATP synthase activity, rotational mechanism	0.003999	4
GO: Molecular Function	GO:0070410	co-SMAD binding	0.005465	4
GO: Molecular Function	GO:0044183	protein binding involved in protein folding	0.01162	4
GO: Molecular Function	GO:0023026	MHC class II protein complex binding	0.01424	4
GO: Molecular Function	GO:0023023	MHC protein complex binding	0.026	4
GO: Molecular Function	GO:0042288	MHC class I protein binding	0.0337	4
GO: Molecular Function	GO:0090079	translation regulator activity, nucleic acid binding	0.03764	4
GO: Molecular Function	GO:0070412	R-SMAD binding	0.04824	4
GO: Molecular Function	GO:1990932	5.8S rRNA binding	0.0005745	3
GO: Molecular Function	GO:0002134	UTP binding	0.001913	3
GO: Molecular Function	GO:0032551	pyrimidine ribonucleoside binding	0.001913	3
GO: Molecular Function	GO:0004826	phenylalanine-tRNA ligase activity	0.00417	3
GO: Molecular Function	GO:0001884	pyrimidine nucleoside binding	0.00417	3
GO: Molecular Function	GO:1990446	U1 snRNP binding	0.00417	3
GO: Molecular Function	GO:0032557	pyrimidine ribonucleotide binding	0.00417	3
GO: Molecular Function	GO:0008379	thioredoxin peroxidase activity	0.00745	3
GO: Molecular Function	GO:0032554	purine deoxyribonucleotide binding	0.00745	3
GO: Molecular Function	GO:0008312	7S RNA binding	0.01162	3

GO: Molecular Function	GO:1990247	N6-methyladenosine-containing RNA binding	0.01162	3
GO: Molecular Function	GO:0070990	snRNP binding	0.01162	3
GO: Molecular Function	GO:0001094	TFIID-class transcription factor binding	0.01162	3
GO: Molecular Function	GO:0001091	RNA polymerase II basal transcription factor binding	0.01648	3
GO: Molecular Function	GO:0000774	adenyl-nucleotide exchange factor activity	0.02926	3
GO: Molecular Function	GO:0035613	RNA stem-loop binding	0.02926	3
GO: Molecular Function	GO:0030235	nitric-oxide synthase regulator activity	0.02926	3
GO: Molecular Function	GO:0070411	I-SMAD binding	0.03642	3
GO: Molecular Function	GO:0098847	sequence-specific single stranded DNA binding	0.04425	3
GO: Molecular Function	GO:0002135	CTP binding	0.01162	2
GO: Molecular Function	GO:0003938	IMP dehydrogenase activity	0.01162	2
GO: Molecular Function	GO:0032556	pyrimidine deoxyribonucleotide binding	0.01162	2
GO: Molecular Function	GO:0004487	methylenetetrahydrofolate dehydrogenase (NAD+) activity	0.02794	2
GO: Molecular Function	GO:1990825	sequence-specific mRNA binding	0.02794	2
GO: Molecular Function	GO:0004827	proline-tRNA ligase activity	0.02794	2
GO: Molecular Function	GO:1990829	C-rich single-stranded DNA binding	0.02794	2
GO: Molecular Function	GO:0017098	sulfonylurea receptor binding	0.02794	2
GO: Molecular Function	GO:0032564	dATP binding	0.04425	2
GO: Molecular Function	GO:0004477	methenyltetrahydrofolate cyclohydrolase activity	0.04425	2
GO: Molecular Function	GO:0004329	formate-tetrahydrofolate ligase activity	0.04425	2
GO: Molecular Function	GO:1990715	mRNA CDS binding	0.04425	2
GO: Molecular Function	GO:0004488	methylenetetrahydrofolate dehydrogenase (NADP+) activity	0.04425	2
**Biological Processes**				
GO: Biological Process	GO:1901566	organonitrogen compound biosynthetic process	1.777E-83	208
GO: Biological Process	GO:0006396	RNA processing	2.204E-111	197
GO: Biological Process	GO:0043603	cellular amide metabolic process	7.743E-95	190
GO: Biological Process	GO:0043604	amide biosynthetic process	1.413E-112	186
GO: Biological Process	GO:0006518	peptide metabolic process	1.16E-102	183
GO: Biological Process	GO:0006412	translation	1.359E-120	182
GO: Biological Process	GO:0043043	peptide biosynthetic process	2.881E-117	182

GO: Biological Process	GO:0016071	mRNA metabolic process	1.287E-116	178
GO: Biological Process	GO:0044248	cellular catabolic process	4.033E-29	153
GO: Biological Process	GO:1901575	organic substance catabolic process	1.816E-25	150
GO: Biological Process	GO:0070727	cellular macromolecule localization	4.168E-31	149
GO: Biological Process	GO:0034613	cellular protein localization	1.019E-29	146
GO: Biological Process	GO:0015031	protein transport	1.568E-18	137
GO: Biological Process	GO:0065003	protein-containing complex assembly	1.235E-20	136
GO: Biological Process	GO:0009057	macromolecule catabolic process	9.338E-36	132
GO: Biological Process	GO:0033365	protein localization to organelle	5.944E-49	131
GO: Biological Process	GO:0022613	ribonucleoprotein complex biogenesis	2.351E-85	130
GO: Biological Process	GO:0044419	interspecies interaction between organisms	9.075E-55	130
GO: Biological Process	GO:0044403	symbiont process	9.075E-55	130
GO: Biological Process	GO:0044764	multi-organism cellular process	3.465E-56	129
GO: Biological Process	GO:0034660	ncRNA metabolic process	1.914E-72	128
GO: Biological Process	GO:0016032	viral process	3.024E-56	128
GO: Biological Process	GO:0044265	cellular macromolecule catabolic process	7.624E-43	127
GO: Biological Process	GO:0006886	intracellular protein transport	2.107E-32	120
GO: Biological Process	GO:0072594	establishment of protein localization to organelle	4.327E-54	119
GO: Biological Process	GO:0034655	nucleobase-containing compound catabolic process	1.919E-74	115
GO: Biological Process	GO:0044270	cellular nitrogen compound catabolic process	3.025E-69	115
GO: Biological Process	GO:0046700	heterocycle catabolic process	4.917E-69	115
GO: Biological Process	GO:0019439	aromatic compound catabolic process	6.108E-68	115
GO: Biological Process	GO:1901361	organic cyclic compound catabolic process	3.371E-64	115
GO: Biological Process	GO:0006605	protein targeting	7.036E-45	112
GO: Biological Process	GO:0034470	ncRNA processing	2.537E-72	111
GO: Biological Process	GO:0051173	positive regulation of nitrogen compound metabolic process	7.682E-09	111
GO: Biological Process	GO:0061024	membrane organization	1.891E-25	108
GO: Biological Process	GO:0042254	ribosome biogenesis	4.68E-76	105
GO: Biological Process	GO:0019058	viral life cycle	4.296E-59	104
GO: Biological Process	GO:0051172	negative regulation of nitrogen compound metabolic process	9.169E-11	104

GO: Biological Process	GO:0006401	RNA catabolic process	7.224E-86	102
GO: Biological Process	GO:0010628	positive regulation of gene expression	0.000001509	101
GO: Biological Process	GO:0010629	negative regulation of gene expression	3.059E-09	99
GO: Biological Process	GO:0006402	mRNA catabolic process	8.669E-87	98
GO: Biological Process	GO:0072657	protein localization to membrane	4.033E-46	97
GO: Biological Process	GO:0031328	positive regulation of cellular biosynthetic process	0.00005648	96
GO: Biological Process	GO:0009891	positive regulation of biosynthetic process	0.0001241	96
GO: Biological Process	GO:0006364	rRNA processing	7.725E-74	95
GO: Biological Process	GO:0016072	rRNA metabolic process	1.4E-72	95
GO: Biological Process	GO:0006413	translational initiation	5.611E-87	94
GO: Biological Process	GO:0000956	nuclear-transcribed mRNA catabolic process	5.987E-85	94
GO: Biological Process	GO:0090150	establishment of protein localization to membrane	5.17E-56	94
GO: Biological Process	GO:0034622	cellular protein-containing complex assembly	5.091E-20	93
GO: Biological Process	GO:0051276	chromosome organization	9.413E-15	93
GO: Biological Process	GO:0009890	negative regulation of biosynthetic process	3.209E-07	93
GO: Biological Process	GO:0010557	positive regulation of macromolecule biosynthetic process	0.00001272	93
GO: Biological Process	GO:0045935	positive regulation of nucleobase-containing compound metabolic process	0.0001323	91
GO: Biological Process	GO:0031327	negative regulation of cellular biosynthetic process	0.000001069	90
GO: Biological Process	GO:0044033	multi-organism metabolic process	1.863E-76	89
GO: Biological Process	GO:0010558	negative regulation of macromolecule biosynthetic process	3.323E-07	89
GO: Biological Process	GO:0019080	viral gene expression	2.239E-78	87
GO: Biological Process	GO:0000184	nuclear-transcribed mRNA catabolic process, nonsense-mediated decay	4.251E-100	86
GO: Biological Process	GO:0008380	RNA splicing	2.953E-44	85
GO: Biological Process	GO:0006397	mRNA processing	4.903E-38	85
GO: Biological Process	GO:0019083	viral transcription	8.866E-77	84
GO: Biological Process	GO:2000113	negative regulation of cellular macromolecule biosynthetic process	8.432E-07	84
GO: Biological Process	GO:0006612	protein targeting to membrane	5.632E-71	83
GO: Biological Process	GO:0006614	SRP-dependent cotranslational protein targeting to membrane	8.333E-107	81
GO: Biological Process	GO:0006613	cotranslational protein targeting to membrane	1.147E-101	81
GO: Biological Process	GO:0045047	protein targeting to ER	4.819E-101	81

GO: Biological Process	GO:0072599	establishment of protein localization to endoplasmic reticulum	1.389E-98	81
GO: Biological Process	GO:0070972	protein localization to endoplasmic reticulum	6.724E-89	81
GO: Biological Process	GO:0045934	negative regulation of nucleobase-containing compound metabolic process	0.00002718	81
GO: Biological Process	GO:0012501	programmed cell death	0.04574	81
GO: Biological Process	GO:0007049	cell cycle	0.01019	79
GO: Biological Process	GO:0010608	posttranscriptional regulation of gene expression	1.216E-30	76
GO: Biological Process	GO:0033043	regulation of organelle organization	6.243E-08	76
GO: Biological Process	GO:0051253	negative regulation of RNA metabolic process	0.0000168	76
GO: Biological Process	GO:0051254	positive regulation of RNA metabolic process	0.003805	75
GO: Biological Process	GO:0000375	RNA splicing, via transesterification reactions	2.754E-42	74
GO: Biological Process	GO:0080134	regulation of response to stress	0.004583	74
GO: Biological Process	GO:0000398	mRNA splicing, via spliceosome	1.094E-41	73
GO: Biological Process	GO:0000377	RNA splicing, via transesterification reactions with bulged adenosine as nucleophile	1.094E-41	73
GO: Biological Process	GO:0010941	regulation of cell death	0.03585	71
GO: Biological Process	GO:0022402	cell cycle process	0.0008821	70
GO: Biological Process	GO:0051247	positive regulation of protein metabolic process	0.03139	70
GO: Biological Process	GO:0032270	positive regulation of cellular protein metabolic process	0.01291	69
GO: Biological Process	GO:0006259	DNA metabolic process	0.000000418	65
GO: Biological Process	GO:0051130	positive regulation of cellular component organization	0.000946	65
GO: Biological Process	GO:0007005	mitochondrion organization	8.852E-10	61
GO: Biological Process	GO:0051248	negative regulation of protein metabolic process	0.001477	61
GO: Biological Process	GO:1902679	negative regulation of RNA biosynthetic process	0.01716	61
GO: Biological Process	GO:1903507	negative regulation of nucleic acid-templated transcription	0.01996	60
GO: Biological Process	GO:0032269	negative regulation of cellular protein metabolic process	0.0009289	59
GO: Biological Process	GO:0006417	regulation of translation	2.847E-23	57
GO: Biological Process	GO:0034248	regulation of cellular amide metabolic process	2.925E-21	57
GO: Biological Process	GO:0000278	mitotic cell cycle	0.0003045	57
GO: Biological Process	GO:1903047	mitotic cell cycle process	0.00005299	56
GO: Biological Process	GO:0045892	negative regulation of transcription, DNA-templated	0.04663	56
GO: Biological Process	GO:0006913	nucleocytoplasmic transport	1.562E-14	55

GO: Biological Process	GO:0051169	nuclear transport	3.119E-14	55
GO: Biological Process	GO:0022411	cellular component disassembly	5.224E-09	54
GO: Biological Process	GO:0022618	ribonucleoprotein complex assembly	2.546E-30	52
GO: Biological Process	GO:0071826	ribonucleoprotein complex subunit organization	3.008E-29	52
GO: Biological Process	GO:0010638	positive regulation of organelle organization	2.695E-08	50
GO: Biological Process	GO:0006325	chromatin organization	0.00002333	50
GO: Biological Process	GO:0080135	regulation of cellular response to stress	0.0002608	46
GO: Biological Process	GO:0006403	RNA localization	9.369E-23	45
GO: Biological Process	GO:0071705	nitrogen compound transport	0.02776	43
GO: Biological Process	GO:0070201	regulation of establishment of protein localization	0.03416	43
GO: Biological Process	GO:0006414	translational elongation	1.008E-28	42
GO: Biological Process	GO:0045087	innate immune response	0.02475	42
GO: Biological Process	GO:1903827	regulation of cellular protein localization	0.00005699	41
GO: Biological Process	GO:0070925	organelle assembly	0.000396	41
GO: Biological Process	GO:0055086	nucleobase-containing small molecule metabolic process	0.008044	41
GO: Biological Process	GO:0006974	cellular response to DNA damage stimulus	0.01726	41
GO: Biological Process	GO:0032984	protein-containing complex disassembly	4.02E-14	40
GO: Biological Process	GO:0044770	cell cycle phase transition	0.00002349	40
GO: Biological Process	GO:0044772	mitotic cell cycle phase transition	0.00004619	38
GO: Biological Process	GO:0009894	regulation of catabolic process	0.04186	38
GO: Biological Process	GO:0043624	cellular protein complex disassembly	1.003E-15	37
GO: Biological Process	GO:0010942	positive regulation of cell death	0.01532	37
GO: Biological Process	GO:1903829	positive regulation of cellular protein localization	1.541E-07	36
GO: Biological Process	GO:0007017	microtubule-based process	0.01188	36
GO: Biological Process	GO:0033044	regulation of chromosome organization	9.923E-10	35
GO: Biological Process	GO:0006753	nucleoside phosphate metabolic process	0.04326	35
GO: Biological Process	GO:0051052	regulation of DNA metabolic process	5.988E-07	34
GO: Biological Process	GO:1904951	positive regulation of establishment of protein localization	0.003713	34
GO: Biological Process	GO:0043065	positive regulation of apoptotic process	0.02686	34
GO: Biological Process	GO:0043068	positive regulation of programmed cell death	0.02984	34

GO: Biological Process	GO:0031329	regulation of cellular catabolic process	0.03416	34
GO: Biological Process	GO:0051301	cell division	0.03682	34
GO: Biological Process	GO:0032386	regulation of intracellular transport	0.03897	34
GO: Biological Process	GO:0032543	mitochondrial translation	1.854E-20	33
GO: Biological Process	GO:0034504	protein localization to nucleus	0.000007289	33
GO: Biological Process	GO:0071103	DNA conformation change	1.562E-08	32
GO: Biological Process	GO:0010564	regulation of cell cycle process	0.02596	32
GO: Biological Process	GO:0006415	translational termination	4.856E-21	31
GO: Biological Process	GO:0050657	nucleic acid transport	4.215E-13	31
GO: Biological Process	GO:0050658	RNA transport	4.215E-13	31
GO: Biological Process	GO:0051236	establishment of RNA localization	6.704E-13	31
GO: Biological Process	GO:0015931	nucleobase-containing compound transport	6.015E-11	31
GO: Biological Process	GO:0006457	protein folding	6.221E-10	31
GO: Biological Process	GO:0006260	DNA replication	0.000004698	31
GO: Biological Process	GO:0016569	covalent chromatin modification	0.001114	31
GO: Biological Process	GO:0043900	regulation of multi-organism process	0.004976	31
GO: Biological Process	GO:0070125	mitochondrial translational elongation	4.566E-22	30
GO: Biological Process	GO:0051168	nuclear export	9.852E-12	30
GO: Biological Process	GO:0016570	histone modification	0.001412	30
GO: Biological Process	GO:0051259	protein complex oligomerization	0.02079	30
GO: Biological Process	GO:0071407	cellular response to organic cyclic compound	0.03223	30
GO: Biological Process	GO:0070126	mitochondrial translational termination	1.098E-20	29
GO: Biological Process	GO:0060249	anatomical structure homeostasis	0.00058	29
GO: Biological Process	GO:0043488	regulation of mRNA stability	4.184E-13	28
GO: Biological Process	GO:0043487	regulation of RNA stability	8.295E-13	28
GO: Biological Process	GO:0006281	DNA repair	0.03483	28
GO: Biological Process	GO:1903649	regulation of cytoplasmic transport	0.03667	28
GO: Biological Process	GO:1903311	regulation of mRNA metabolic process	2.315E-13	27
GO: Biological Process	GO:0017148	negative regulation of translation	3.537E-12	27
GO: Biological Process	GO:0034249	negative regulation of cellular amide metabolic process	1.554E-11	27

GO: Biological Process	GO:0051170	import into nucleus	0.00008097	27
GO: Biological Process	GO:0018205	peptidyl-lysine modification	0.006558	27
GO: Biological Process	GO:0040029	regulation of gene expression, epigenetic	0.000007851	26
GO: Biological Process	GO:0006520	cellular amino acid metabolic process	0.006183	26
GO: Biological Process	GO:0009116	nucleoside metabolic process	0.008695	26
GO: Biological Process	GO:0009615	response to virus	0.01349	26
GO: Biological Process	GO:1901657	glycosyl compound metabolic process	0.01651	26
GO: Biological Process	GO:0042255	ribosome assembly	4.299E-21	25
GO: Biological Process	GO:0042273	ribosomal large subunit biogenesis	1.182E-20	25
GO: Biological Process	GO:0043484	regulation of RNA splicing	2.128E-13	25
GO: Biological Process	GO:0034250	positive regulation of cellular amide metabolic process	5.373E-12	25
GO: Biological Process	GO:0032200	telomere organization	5.069E-10	25
GO: Biological Process	GO:0009123	nucleoside monophosphate metabolic process	0.000256	25
GO: Biological Process	GO:0002181	cytoplasmic translation	1.961E-20	24
GO: Biological Process	GO:0042274	ribosomal small subunit biogenesis	3.863E-18	24
GO: Biological Process	GO:0045727	positive regulation of translation	9.564E-13	24
GO: Biological Process	GO:0031123	RNA 3'-end processing	1.628E-11	24
GO: Biological Process	GO:0000723	telomere maintenance	4.611E-10	24
GO: Biological Process	GO:0051028	mRNA transport	2.213E-09	24
GO: Biological Process	GO:2001252	positive regulation of chromosome organization	4.352E-09	24
GO: Biological Process	GO:1900180	regulation of protein localization to nucleus	0.00001063	24
GO: Biological Process	GO:0032259	methylation	0.00456	24
GO: Biological Process	GO:0009119	ribonucleoside metabolic process	0.01726	24
GO: Biological Process	GO:2001233	regulation of apoptotic signaling pathway	0.02728	24
GO: Biological Process	GO:0006353	DNA-templated transcription, termination	3.55E-12	23
GO: Biological Process	GO:0031647	regulation of protein stability	0.00004162	23
GO: Biological Process	GO:0009161	ribonucleoside monophosphate metabolic process	0.0008984	23
GO: Biological Process	GO:0071824	protein-DNA complex subunit organization	0.0003255	22
GO: Biological Process	GO:0009167	purine ribonucleoside monophosphate metabolic process	0.001293	22
GO: Biological Process	GO:0009126	purine nucleoside monophosphate metabolic process	0.001353	22

GO: Biological Process	GO:1901987	regulation of cell cycle phase transition	0.01342	22
GO: Biological Process	GO:0017038	protein import	0.01971	22
GO: Biological Process	GO:0050684	regulation of mRNA processing	1.223E-09	21
GO: Biological Process	GO:0006405	RNA export from nucleus	5.28E-09	21
GO: Biological Process	GO:0071426	ribonucleoprotein complex export from nucleus	5.28E-09	21
GO: Biological Process	GO:0071166	ribonucleoprotein complex localization	8.394E-09	21
GO: Biological Process	GO:0006275	regulation of DNA replication	0.000003081	21
GO: Biological Process	GO:0006399	tRNA metabolic process	0.000006453	21
GO: Biological Process	GO:0051054	positive regulation of DNA metabolic process	0.00004199	21
GO: Biological Process	GO:0044839	cell cycle G2/M phase transition	0.00004871	21
GO: Biological Process	GO:0006323	DNA packaging	0.00005241	21
GO: Biological Process	GO:0071897	DNA biosynthetic process	0.0000747	21
GO: Biological Process	GO:0065004	protein-DNA complex assembly	0.0001934	21
GO: Biological Process	GO:0009266	response to temperature stimulus	0.0003781	21
GO: Biological Process	GO:0043414	macromolecule methylation	0.002496	21
GO: Biological Process	GO:0097193	intrinsic apoptotic signaling pathway	0.007922	21
GO: Biological Process	GO:1901990	regulation of mitotic cell cycle phase transition	0.01425	21
GO: Biological Process	GO:0007059	chromosome segregation	0.02287	21
GO: Biological Process	GO:0010639	negative regulation of organelle organization	0.02513	21
GO: Biological Process	GO:0048024	regulation of mRNA splicing, via spliceosome	1.623E-12	20
GO: Biological Process	GO:1900182	positive regulation of protein localization to nucleus	3.662E-07	20
GO: Biological Process	GO:0050821	protein stabilization	5.907E-07	20
GO: Biological Process	GO:0016458	gene silencing	0.002848	20
GO: Biological Process	GO:0006606	protein import into nucleus	0.01726	20
GO: Biological Process	GO:0009165	nucleotide biosynthetic process	0.02984	20
GO: Biological Process	GO:1901293	nucleoside phosphate biosynthetic process	0.0319	20
GO: Biological Process	GO:0051098	regulation of binding	0.03897	20
GO: Biological Process	GO:0006369	termination of RNA polymerase II transcription	1.301E-12	19
GO: Biological Process	GO:0031497	chromatin assembly	0.00002049	19
GO: Biological Process	GO:0006333	chromatin assembly or disassembly	0.0001667	19

GO: Biological Process	GO:0050792	regulation of viral process	0.0001792	19
GO: Biological Process	GO:0043903	regulation of symbiosis, encompassing mutualism through parasitism	0.001213	19
GO: Biological Process	GO:0046822	regulation of nucleocytoplasmic transport	0.002809	19
GO: Biological Process	GO:0000082	G1/S transition of mitotic cell cycle	0.004709	19
GO: Biological Process	GO:0044843	cell cycle G1/S phase transition	0.007207	19
GO: Biological Process	GO:0009141	nucleoside triphosphate metabolic process	0.02717	19
GO: Biological Process	GO:0032204	regulation of telomere maintenance	9.169E-11	18
GO: Biological Process	GO:0031124	mRNA 3'-end processing	1.584E-08	18
GO: Biological Process	GO:1903900	regulation of viral life cycle	0.0002404	18
GO: Biological Process	GO:0000086	G2/M transition of mitotic cell cycle	0.0009194	18
GO: Biological Process	GO:0009895	negative regulation of catabolic process	0.007288	18
GO: Biological Process	GO:0000302	response to reactive oxygen species	0.01333	18
GO: Biological Process	GO:0090305	nucleic acid phosphodiester bond hydrolysis	0.02632	18
GO: Biological Process	GO:0006839	mitochondrial transport	0.03465	18
GO: Biological Process	GO:1903320	regulation of protein modification by small protein conjugation or removal	0.04326	18
GO: Biological Process	GO:0010833	telomere maintenance via telomere lengthening	5.444E-10	17
GO: Biological Process	GO:0009124	nucleoside monophosphate biosynthetic process	1.566E-07	17
GO: Biological Process	GO:0034728	nucleosome organization	0.0005561	17
GO: Biological Process	GO:0009408	response to heat	0.0005561	17
GO: Biological Process	GO:0007179	transforming growth factor beta receptor signaling pathway	0.001249	17
GO: Biological Process	GO:0001649	osteoblast differentiation	0.00948	17
GO: Biological Process	GO:0071560	cellular response to transforming growth factor beta stimulus	0.0129	17
GO: Biological Process	GO:0071559	response to transforming growth factor beta	0.01424	17
GO: Biological Process	GO:0034599	cellular response to oxidative stress	0.02375	17
GO: Biological Process	GO:0072331	signal transduction by p53 class mediator	0.03764	17
GO: Biological Process	GO:0009636	response to toxic substance	0.04543	17
GO: Biological Process	GO:0000245	spliceosomal complex assembly	5.248E-10	16
GO: Biological Process	GO:0006278	RNA-dependent DNA biosynthetic process	4.278E-09	16
GO: Biological Process	GO:0006406	mRNA export from nucleus	0.000005339	16
GO: Biological Process	GO:0071427	mRNA-containing ribonucleoprotein complex export from nucleus	0.000005339	16

GO: Biological Process	GO:0006334	nucleosome assembly	0.0002561	16
GO: Biological Process	GO:0031047	gene silencing by RNA	0.0007876	16
GO: Biological Process	GO:0007623	circadian rhythm	0.015	16
GO: Biological Process	GO:0000819	sister chromatid segregation	0.02311	16
GO: Biological Process	GO:0000075	cell cycle checkpoint	0.03064	16
GO: Biological Process	GO:0009156	ribonucleoside monophosphate biosynthetic process	0.000001889	15
GO: Biological Process	GO:2000278	regulation of DNA biosynthetic process	0.0000115	15
GO: Biological Process	GO:0019079	viral genome replication	0.0000168	15
GO: Biological Process	GO:0051262	protein tetramerization	0.001524	15
GO: Biological Process	GO:2001242	regulation of intrinsic apoptotic signaling pathway	0.002602	15
GO: Biological Process	GO:0043902	positive regulation of multi-organism process	0.004314	15
GO: Biological Process	GO:0006479	protein methylation	0.005864	15
GO: Biological Process	GO:0008213	protein alkylation	0.005864	15
GO: Biological Process	GO:0070585	protein localization to mitochondrion	0.01437	15
GO: Biological Process	GO:0010821	regulation of mitochondrion organization	0.04547	15
GO: Biological Process	GO:1903312	negative regulation of mRNA metabolic process	1.454E-09	14
GO: Biological Process	GO:1904356	regulation of telomere maintenance via telomere lengthening	9.136E-09	14
GO: Biological Process	GO:0007004	telomere maintenance via telomerase	4.45E-08	14
GO: Biological Process	GO:0009168	purine ribonucleoside monophosphate biosynthetic process	0.000001798	14
GO: Biological Process	GO:0009127	purine nucleoside monophosphate biosynthetic process	0.000001798	14
GO: Biological Process	GO:0043331	response to dsRNA	0.000009883	14
GO: Biological Process	GO:0045740	positive regulation of DNA replication	0.00003229	14
GO: Biological Process	GO:1902275	regulation of chromatin organization	0.01144	14
GO: Biological Process	GO:0031330	negative regulation of cellular catabolic process	0.02577	14
GO: Biological Process	GO:0072655	establishment of protein localization to mitochondrion	0.02678	14
GO: Biological Process	GO:0009566	fertilization	0.02754	14
GO: Biological Process	GO:2001235	positive regulation of apoptotic signaling pathway	0.03139	14
GO: Biological Process	GO:0000028	ribosomal small subunit assembly	9.34E-14	13
GO: Biological Process	GO:0033119	negative regulation of RNA splicing	1.392E-10	13
GO: Biological Process	GO:0032210	regulation of telomere maintenance via telomerase	2.519E-08	13

GO: Biological Process	GO:0045069	regulation of viral genome replication	0.00002807	13
GO: Biological Process	GO:0034605	cellular response to heat	0.000971	13
GO: Biological Process	GO:0009163	nucleoside biosynthetic process	0.003929	13
GO: Biological Process	GO:1901659	glycosyl compound biosynthetic process	0.004518	13
GO: Biological Process	GO:0016571	histone methylation	0.005372	13
GO: Biological Process	GO:0007338	single fertilization	0.00764	13
GO: Biological Process	GO:0031056	regulation of histone modification	0.008973	13
GO: Biological Process	GO:0034614	cellular response to reactive oxygen species	0.01136	13
GO: Biological Process	GO:0043901	negative regulation of multi-organism process	0.02475	13
GO: Biological Process	GO:0048255	mRNA stabilization	1.791E-08	12
GO: Biological Process	GO:0043489	RNA stabilization	2.538E-08	12
GO: Biological Process	GO:0032206	positive regulation of telomere maintenance	4.783E-07	12
GO: Biological Process	GO:2000573	positive regulation of DNA biosynthetic process	0.00001096	12
GO: Biological Process	GO:1900034	regulation of cellular response to heat	0.00009238	12
GO: Biological Process	GO:0032479	regulation of type I interferon production	0.004736	12
GO: Biological Process	GO:0032606	type I interferon production	0.005489	12
GO: Biological Process	GO:0042455	ribonucleoside biosynthetic process	0.006183	12
GO: Biological Process	GO:1903747	regulation of establishment of protein localization to mitochondrion	0.01019	12
GO: Biological Process	GO:0042177	negative regulation of protein catabolic process	0.01155	12
GO: Biological Process	GO:0042542	response to hydrogen peroxide	0.02446	12
GO: Biological Process	GO:0007093	mitotic cell cycle checkpoint	0.04585	12
GO: Biological Process	GO:0000027	ribosomal large subunit assembly	1.018E-09	11
GO: Biological Process	GO:0048025	negative regulation of mRNA splicing, via spliceosome	5.039E-09	11
GO: Biological Process	GO:0032212	positive regulation of telomere maintenance via telomerase	6.244E-08	11
GO: Biological Process	GO:1904358	positive regulation of telomere maintenance via telomere lengthening	8.976E-08	11
GO: Biological Process	GO:0050686	negative regulation of mRNA processing	3.375E-07	11
GO: Biological Process	GO:0000380	alternative mRNA splicing, via spliceosome	0.00000465	11
GO: Biological Process	GO:1903313	positive regulation of mRNA metabolic process	0.000008643	11
GO: Biological Process	GO:0034502	protein localization to chromosome	0.00006179	11
GO: Biological Process	GO:0032508	DNA duplex unwinding	0.0004787	11

GO: Biological Process	GO:0032392	DNA geometric change	0.0008308	11
GO: Biological Process	GO:0031058	positive regulation of histone modification	0.002154	11
GO: Biological Process	GO:0048524	positive regulation of viral process	0.002809	11
GO: Biological Process	GO:1903844	regulation of cellular response to transforming growth factor beta stimulus	0.006585	11
GO: Biological Process	GO:0017015	regulation of transforming growth factor beta receptor signaling pathway	0.006585	11
GO: Biological Process	GO:0007062	sister chromatid cohesion	0.02446	11
GO: Biological Process	GO:0008637	apoptotic mitochondrial changes	0.02678	11
GO: Biological Process	GO:0031397	negative regulation of protein ubiquitination	0.03139	11
GO: Biological Process	GO:0016925	protein sumoylation	0.04241	11
GO: Biological Process	GO:1903321	negative regulation of protein modification by small protein conjugation or removal	0.04814	11
GO: Biological Process	GO:1904874	positive regulation of telomerase RNA localization to Cajal body	1.028E-10	10
GO: Biological Process	GO:1904872	regulation of telomerase RNA localization to Cajal body	1.028E-10	10
GO: Biological Process	GO:0090672	telomerase RNA localization	2.565E-10	10
GO: Biological Process	GO:0090670	RNA localization to Cajal body	2.565E-10	10
GO: Biological Process	GO:0090671	telomerase RNA localization to Cajal body	2.565E-10	10
GO: Biological Process	GO:0033120	positive regulation of RNA splicing	0.000001233	10
GO: Biological Process	GO:0042769	DNA damage response, detection of DNA damage	0.000002998	10
GO: Biological Process	GO:0000387	spliceosomal snRNP assembly	0.000006534	10
GO: Biological Process	GO:0006446	regulation of translational initiation	0.004567	10
GO: Biological Process	GO:1903902	positive regulation of viral life cycle	0.00585	10
GO: Biological Process	GO:0046129	purine ribonucleoside biosynthetic process	0.01679	10
GO: Biological Process	GO:0042451	purine nucleoside biosynthetic process	0.01679	10
GO: Biological Process	GO:0098781	ncRNA transcription	0.01781	10
GO: Biological Process	GO:0007006	mitochondrial membrane organization	0.01781	10
GO: Biological Process	GO:0071156	regulation of cell cycle arrest	0.02375	10
GO: Biological Process	GO:0051053	negative regulation of DNA metabolic process	0.03295	10
GO: Biological Process	GO:1903749	positive regulation of establishment of protein localization to mitochondrion	0.03457	10
GO: Biological Process	GO:0042770	signal transduction in response to DNA damage	0.04569	10
GO: Biological Process	GO:0034101	erythrocyte homeostasis	0.04757	10
GO: Biological Process	GO:0051340	regulation of ligase activity	0.04757	10

GO: Biological Process	GO:0070198	protein localization to chromosome, telomeric region	0.000002272	9
GO: Biological Process	GO:0006376	mRNA splice site selection	0.000006079	9
GO: Biological Process	GO:0000462	maturation of SSU-rRNA from tricistronic rRNA transcript (SSU-rRNA, 5.8S rRNA, LSU-rRNA)	0.00002349	9
GO: Biological Process	GO:1901998	toxin transport	0.0001637	9
GO: Biological Process	GO:0030490	maturation of SSU-rRNA	0.0001954	9
GO: Biological Process	GO:0071359	cellular response to dsRNA	0.0003807	9
GO: Biological Process	GO:0006418	tRNA aminoacylation for protein translation	0.0003807	9
GO: Biological Process	GO:0043039	tRNA aminoacylation	0.0006012	9
GO: Biological Process	GO:0043038	amino acid activation	0.0006901	9
GO: Biological Process	GO:0035195	gene silencing by miRNA	0.0008966	9
GO: Biological Process	GO:1903201	regulation of oxidative stress-induced cell death	0.0008966	9
GO: Biological Process	GO:0016441	posttranscriptional gene silencing	0.001477	9
GO: Biological Process	GO:0035194	posttranscriptional gene silencing by RNA	0.001477	9
GO: Biological Process	GO:1900407	regulation of cellular response to oxidative stress	0.004567	9
GO: Biological Process	GO:0036473	cell death in response to oxidative stress	0.006558	9
GO: Biological Process	GO:1902882	regulation of response to oxidative stress	0.007801	9
GO: Biological Process	GO:1901607	alpha-amino acid biosynthetic process	0.01347	9
GO: Biological Process	GO:0070301	cellular response to hydrogen peroxide	0.02326	9
GO: Biological Process	GO:0008652	cellular amino acid biosynthetic process	0.03364	9
GO: Biological Process	GO:1904869	regulation of protein localization to Cajal body	4.497E-11	8
GO: Biological Process	GO:1904871	positive regulation of protein localization to Cajal body	4.497E-11	8
GO: Biological Process	GO:1990173	protein localization to nucleoplasm	3.395E-10	8
GO: Biological Process	GO:1903405	protein localization to nuclear body	3.395E-10	8
GO: Biological Process	GO:1904867	protein localization to Cajal body	3.395E-10	8
GO: Biological Process	GO:1904816	positive regulation of protein localization to chromosome, telomeric region	1.431E-08	8
GO: Biological Process	GO:1904814	regulation of protein localization to chromosome, telomeric region	7.582E-08	8
GO: Biological Process	GO:0070199	establishment of protein localization to chromosome	0.000007835	8
GO: Biological Process	GO:0000460	maturation of 5.8S rRNA	0.0001098	8
GO: Biological Process	GO:0009303	rRNA transcription	0.000139	8

GO: Biological Process	GO:0000381	regulation of alternative mRNA splicing, via spliceosome	0.0002652	8
GO: Biological Process	GO:0007339	binding of sperm to zona pellucida	0.0003234	8
GO: Biological Process	GO:0009112	nucleobase metabolic process	0.0005634	8
GO: Biological Process	GO:0010501	RNA secondary structure unwinding	0.001232	8
GO: Biological Process	GO:0035036	sperm-egg recognition	0.002172	8
GO: Biological Process	GO:0051568	histone H3-K4 methylation	0.004179	8
GO: Biological Process	GO:0006360	transcription by RNA polymerase I	0.008485	8
GO: Biological Process	GO:0072401	signal transduction involved in DNA integrity checkpoint	0.01331	8
GO: Biological Process	GO:0072422	signal transduction involved in DNA damage checkpoint	0.01331	8
GO: Biological Process	GO:0009988	cell-cell recognition	0.01429	8
GO: Biological Process	GO:0072395	signal transduction involved in cell cycle checkpoint	0.01429	8
GO: Biological Process	GO:0032481	positive regulation of type I interferon production	0.02112	8
GO: Biological Process	GO:0031571	mitotic G1 DNA damage checkpoint	0.02292	8
GO: Biological Process	GO:0044819	mitotic G1/S transition checkpoint	0.02292	8
GO: Biological Process	GO:0044783	G1 DNA damage checkpoint	0.02446	8
GO: Biological Process	GO:0072527	pyrimidine-containing compound metabolic process	0.03465	8
GO: Biological Process	GO:0051444	negative regulation of ubiquitin-protein transferase activity	0.03639	8
GO: Biological Process	GO:0045815	positive regulation of gene expression, epigenetic	0.03639	8
GO: Biological Process	GO:0051352	negative regulation of ligase activity	0.04114	8
GO: Biological Process	GO:1903363	negative regulation of cellular protein catabolic process	0.04367	8
GO: Biological Process	GO:0071158	positive regulation of cell cycle arrest	0.0463	8
GO: Biological Process	GO:1904851	positive regulation of establishment of protein localization to telomere	3.38E-08	7
GO: Biological Process	GO:0070203	regulation of establishment of protein localization to telomere	1.051E-07	7
GO: Biological Process	GO:0070202	regulation of establishment of protein localization to chromosome	2.755E-07	7
GO: Biological Process	GO:0070200	establishment of protein localization to telomere	0.000004267	7
GO: Biological Process	GO:0046112	nucleobase biosynthetic process	0.00001745	7
GO: Biological Process	GO:0042026	protein refolding	0.0001049	7
GO: Biological Process	GO:0008334	histone mRNA metabolic process	0.0003947	7
GO: Biological Process	GO:0071353	cellular response to interleukin-4	0.0004977	7
GO: Biological Process	GO:0045070	positive regulation of viral genome replication	0.0006087	7

GO: Biological Process	GO:0035196	production of miRNAs involved in gene silencing by miRNA	0.000759	7
GO: Biological Process	GO:0031050	dsRNA fragmentation	0.0008984	7
GO: Biological Process	GO:0070918	production of small RNA involved in gene silencing by RNA	0.0008984	7
GO: Biological Process	GO:0070670	response to interleukin-4	0.001305	7
GO: Biological Process	GO:0050685	positive regulation of mRNA processing	0.00219	7
GO: Biological Process	GO:1903146	regulation of autophagy of mitochondrion	0.006717	7
GO: Biological Process	GO:0061077	chaperone-mediated protein folding	0.01428	7
GO: Biological Process	GO:2001244	positive regulation of intrinsic apoptotic signaling pathway	0.02984	7
GO: Biological Process	GO:0006977	DNA damage response, signal transduction by p53 class mediator resulting in cell cycle arrest	0.03172	7
GO: Biological Process	GO:1902400	intracellular signal transduction involved in G1 DNA damage checkpoint	0.03416	7
GO: Biological Process	GO:0072431	signal transduction involved in mitotic G1 DNA damage checkpoint	0.03416	7
GO: Biological Process	GO:1902403	signal transduction involved in mitotic DNA integrity checkpoint	0.03618	7
GO: Biological Process	GO:0072413	signal transduction involved in mitotic cell cycle checkpoint	0.03618	7
GO: Biological Process	GO:0006611	protein export from nucleus	0.03618	7
GO: Biological Process	GO:1902402	signal transduction involved in mitotic DNA damage checkpoint	0.03618	7
GO: Biological Process	GO:0009201	ribonucleoside triphosphate biosynthetic process	0.03897	7
GO: Biological Process	GO:0043462	regulation of ATPase activity	0.04152	7
GO: Biological Process	GO:1901983	regulation of protein acetylation	0.04754	7
GO: Biological Process	GO:1903845	negative regulation of cellular response to transforming growth factor beta stimulus	0.0495	7
GO: Biological Process	GO:0030512	negative regulation of transforming growth factor beta receptor signaling pathway	0.0495	7
GO: Biological Process	GO:0009113	purine nucleobase biosynthetic process	0.00001871	6
GO: Biological Process	GO:0070935	3'-UTR-mediated mRNA stabilization	0.00005361	6
GO: Biological Process	GO:0048026	positive regulation of mRNA splicing, via spliceosome	0.000266	6
GO: Biological Process	GO:0006144	purine nucleobase metabolic process	0.0008577	6
GO: Biological Process	GO:0000470	maturation of LSU-rRNA	0.0008577	6
GO: Biological Process	GO:0032205	negative regulation of telomere maintenance	0.002118	6
GO: Biological Process	GO:1903205	regulation of hydrogen peroxide-induced cell death	0.002597	6
GO: Biological Process	GO:0036474	cell death in response to hydrogen peroxide	0.003858	6
GO: Biological Process	GO:1900740	positive regulation of protein insertion into mitochondrial membrane involved in apoptotic signaling pathway	0.004567	6

GO: Biological Process	GO:1902175	regulation of oxidative stress-induced intrinsic apoptotic signaling pathway	0.004567	6
GO: Biological Process	GO:1900739	regulation of protein insertion into mitochondrial membrane involved in apoptotic signaling pathway	0.004567	6
GO: Biological Process	GO:0001844	protein insertion into mitochondrial membrane involved in apoptotic signaling pathway	0.006183	6
GO: Biological Process	GO:0051204	protein insertion into mitochondrial membrane	0.007207	6
GO: Biological Process	GO:1901030	positive regulation of mitochondrial outer membrane permeabilization involved in apoptotic signaling pathway	0.01253	6
GO: Biological Process	GO:1903202	negative regulation of oxidative stress-induced cell death	0.01387	6
GO: Biological Process	GO:1902883	negative regulation of response to oxidative stress	0.01927	6
GO: Biological Process	GO:1900408	negative regulation of cellular response to oxidative stress	0.01927	6
GO: Biological Process	GO:1901031	regulation of response to reactive oxygen species	0.02375	6
GO: Biological Process	GO:0051972	regulation of telomerase activity	0.02375	6
GO: Biological Process	GO:0032781	positive regulation of ATPase activity	0.02596	6
GO: Biological Process	GO:1901028	regulation of mitochondrial outer membrane permeabilization involved in apoptotic signaling pathway	0.02825	6
GO: Biological Process	GO:0008631	intrinsic apoptotic signaling pathway in response to oxidative stress	0.03585	6
GO: Biological Process	GO:0043330	response to exogenous dsRNA	0.03585	6
GO: Biological Process	GO:0051205	protein insertion into membrane	0.03585	6
GO: Biological Process	GO:0045071	negative regulation of viral genome replication	0.04876	6
GO: Biological Process	GO:0006188	IMP biosynthetic process	0.000256	5
GO: Biological Process	GO:0071428	rRNA-containing ribonucleoprotein complex export from nucleus	0.0004022	5
GO: Biological Process	GO:0031125	rRNA 3'-end processing	0.0006087	5
GO: Biological Process	GO:0007183	SMAD protein complex assembly	0.0008858	5
GO: Biological Process	GO:0046040	IMP metabolic process	0.0008858	5
GO: Biological Process	GO:0071025	RNA surveillance	0.0008858	5
GO: Biological Process	GO:0002190	cap-independent translational initiation	0.001232	5
GO: Biological Process	GO:0002183	cytoplasmic translational initiation	0.002259	5
GO: Biological Process	GO:0000466	maturation of 5.8S rRNA from tricistronic rRNA transcript (SSU-rRNA, 5.8S rRNA, LSU-rRNA)	0.005949	5
GO: Biological Process	GO:1903206	negative regulation of hydrogen peroxide-induced cell death	0.005949	5
GO: Biological Process	GO:1901032	negative regulation of response to reactive oxygen species	0.008695	5
GO: Biological Process	GO:0061014	positive regulation of mRNA catabolic process	0.008695	5
GO: Biological Process	GO:0043628	ncRNA 3'-end processing	0.008695	5

GO: Biological Process	GO:0002227	innate immune response in mucosa	0.01049	5
GO: Biological Process	GO:0034661	ncRNA catabolic process	0.01253	5
GO: Biological Process	GO:0006541	glutamine metabolic process	0.01253	5
GO: Biological Process	GO:0061013	regulation of mRNA catabolic process	0.01429	5
GO: Biological Process	GO:0051973	positive regulation of telomerase activity	0.01908	5
GO: Biological Process	GO:0009651	response to salt stress	0.01908	5
GO: Biological Process	GO:0006221	pyrimidine nucleotide biosynthetic process	0.02749	5
GO: Biological Process	GO:0002832	negative regulation of response to biotic stimulus	0.03385	5
GO: Biological Process	GO:0000002	mitochondrial genome maintenance	0.04111	5
GO: Biological Process	GO:0002385	mucosal immune response	0.04557	5
GO: Biological Process	GO:0006296	nucleotide-excision repair, DNA incision, 5'-to lesion	0.0488	5
GO: Biological Process	GO:0046825	regulation of protein export from nucleus	0.0488	5
GO: Biological Process	GO:0070934	CRD-mediated mRNA stabilization	0.0001075	4
GO: Biological Process	GO:0006189	'de novo' IMP biosynthetic process	0.0002908	4
GO: Biological Process	GO:1903608	protein localization to cytoplasmic stress granule	0.0006087	4
GO: Biological Process	GO:0051029	rRNA transport	0.0006087	4
GO: Biological Process	GO:0071027	nuclear RNA surveillance	0.00585	4
GO: Biological Process	GO:0071028	nuclear mRNA surveillance	0.00585	4
GO: Biological Process	GO:0002192	IRES-dependent translational initiation of linear mRNA	0.00585	4
GO: Biological Process	GO:0000244	spliceosomal tri-snRNP complex assembly	0.007801	4
GO: Biological Process	GO:1903203	regulation of oxidative stress-induced neuron death	0.01019	4
GO: Biological Process	GO:0031054	pre-miRNA processing	0.01019	4
GO: Biological Process	GO:0036475	neuron death in response to oxidative stress	0.01301	4
GO: Biological Process	GO:0051131	chaperone-mediated protein complex assembly	0.01301	4
GO: Biological Process	GO:0045793	positive regulation of cell size	0.01927	4
GO: Biological Process	GO:1904357	negative regulation of telomere maintenance via telomere lengthening	0.01927	4
GO: Biological Process	GO:0042776	mitochondrial ATP synthesis coupled proton transport	0.01927	4
GO: Biological Process	GO:0051084	'de novo' posttranslational protein folding	0.02354	4
GO: Biological Process	GO:0016075	rRNA catabolic process	0.02354	4
GO: Biological Process	GO:0042276	error-prone translesion synthesis	0.02728	4

GO: Biological Process	GO:0043153	entrainment of circadian clock by photoperiod	0.02728	4
GO: Biological Process	GO:0050687	negative regulation of defense response to virus	0.02728	4
GO: Biological Process	GO:0070987	error-free translesion synthesis	0.02728	4
GO: Biological Process	GO:1902176	negative regulation of oxidative stress-induced intrinsic apoptotic signaling pathway	0.036	4
GO: Biological Process	GO:0010661	positive regulation of muscle cell apoptotic process	0.036	4
GO: Biological Process	GO:0033145	positive regulation of intracellular steroid hormone receptor signaling pathway	0.036	4
GO: Biological Process	GO:0006458	'de novo' protein folding	0.0412	4
GO: Biological Process	GO:1903427	negative regulation of reactive oxygen species biosynthetic process	0.04721	4
GO: Biological Process	GO:0046653	tetrahydrofolate metabolic process	0.04721	4
GO: Biological Process	GO:0006407	rRNA export from nucleus	0.00205	3
GO: Biological Process	GO:0090666	scaRNA localization to Cajal body	0.00205	3
GO: Biological Process	GO:1903751	negative regulation of intrinsic apoptotic signaling pathway in response to hydrogen peroxide	0.00205	3
GO: Biological Process	GO:1903750	regulation of intrinsic apoptotic signaling pathway in response to hydrogen peroxide	0.00205	3
GO: Biological Process	GO:0006177	GMP biosynthetic process	0.004567	3
GO: Biological Process	GO:2000767	positive regulation of cytoplasmic translation	0.004567	3
GO: Biological Process	GO:0006432	phenylalanyl-tRNA aminoacylation	0.004567	3
GO: Biological Process	GO:0042791	5S class rRNA transcription by RNA polymerase III	0.00806	3
GO: Biological Process	GO:0036481	intrinsic apoptotic signaling pathway in response to hydrogen peroxide	0.00806	3
GO: Biological Process	GO:0042797	tRNA transcription by RNA polymerase III	0.00806	3
GO: Biological Process	GO:0075522	IRES-dependent viral translational initiation	0.01291	3
GO: Biological Process	GO:0009304	tRNA transcription	0.01291	3
GO: Biological Process	GO:0016584	nucleosome positioning	0.01823	3
GO: Biological Process	GO:0034475	U4 snRNA 3'-end processing	0.01823	3
GO: Biological Process	GO:1903376	regulation of oxidative stress-induced neuron intrinsic apoptotic signaling pathway	0.01823	3
GO: Biological Process	GO:0032070	regulation of deoxyribonuclease activity	0.01823	3
GO: Biological Process	GO:0000447	endonucleolytic cleavage in ITS1 to separate SSU-rRNA from 5.8S rRNA and LSU-rRNA from tricistronic rRNA transcript (SSU-rRNA, 5.8S rRNA, LSU-rRNA)	0.01823	3
GO: Biological Process	GO:0046826	negative regulation of protein export from nucleus	0.01823	3
GO: Biological Process	GO:0075713	establishment of integrated proviral latency	0.01823	3
GO: Biological Process	GO:0036480	neuron intrinsic apoptotic signaling pathway in response to oxidative stress	0.01823	3

GO: Biological Process	GO:1901299	negative regulation of hydrogen peroxide-mediated programmed cell death	0.01823	3
GO: Biological Process	GO:0033750	ribosome localization	0.02475	3
GO: Biological Process	GO:0031053	primary miRNA processing	0.02475	3
GO: Biological Process	GO:0034427	nuclear-transcribed mRNA catabolic process, exonucleolytic, 3'-5'	0.02475	3
GO: Biological Process	GO:0009048	dosage compensation by inactivation of X chromosome	0.02475	3
GO: Biological Process	GO:0000054	ribosomal subunit export from nucleus	0.02475	3
GO: Biological Process	GO:0034063	stress granule assembly	0.03139	3
GO: Biological Process	GO:0019081	viral translation	0.03139	3
GO: Biological Process	GO:0019043	establishment of viral latency	0.03139	3
GO: Biological Process	GO:0034472	snRNA 3'-end processing	0.03139	3
GO: Biological Process	GO:0000463	maturation of LSU-rRNA from tricistronic rRNA transcript (SSU-rRNA, 5.8S rRNA, LSU-rRNA)	0.03139	3
GO: Biological Process	GO:1903204	negative regulation of oxidative stress-induced neuron death	0.03139	3
GO: Biological Process	GO:0071474	cellular hyperosmotic response	0.03139	3
GO: Biological Process	GO:0061684	chaperone-mediated autophagy	0.03139	3
GO: Biological Process	GO:0042473	outer ear morphogenesis	0.03139	3
GO: Biological Process	GO:0008298	intracellular mRNA localization	0.03897	3
GO: Biological Process	GO:0010826	negative regulation of centrosome duplication	0.03897	3
GO: Biological Process	GO:0046606	negative regulation of centrosome cycle	0.03897	3
GO: Biological Process	GO:0019042	viral latency	0.03897	3
GO: Biological Process	GO:0045472	response to ether	0.03897	3
GO: Biological Process	GO:0007549	dosage compensation	0.03897	3
GO: Biological Process	GO:0032211	negative regulation of telomere maintenance via telomerase	0.04825	3
GO: Biological Process	GO:1901298	regulation of hydrogen peroxide-mediated programmed cell death	0.04825	3
GO: Biological Process	GO:0033148	positive regulation of intracellular estrogen receptor signaling pathway	0.04825	3
GO: Biological Process	GO:0051085	chaperone cofactor-dependent protein refolding	0.04825	3
GO: Biological Process	GO:1901355	response to rapamycin	0.01324	2
GO: Biological Process	GO:0000461	endonucleolytic cleavage to generate mature 3'-end of SSU-rRNA from (SSU-rRNA, 5.8S rRNA, LSU-rRNA)	0.01324	2
GO: Biological Process	GO:0003365	establishment of cell polarity involved in ameboidal cell migration	0.01324	2
GO: Biological Process	GO:0071475	cellular hyperosmotic salinity response	0.02984	2

GO: Biological Process	GO:0009177	pyrimidine deoxyribonucleoside monophosphate biosynthetic process	0.02984	2
GO: Biological Process	GO:0090367	negative regulation of mRNA modification	0.02984	2
GO: Biological Process	GO:0044413	avoidance of host defenses	0.02984	2
GO: Biological Process	GO:0014076	response to fluoxetine	0.02984	2
GO: Biological Process	GO:0051834	evasion or tolerance of defenses of other organism involved in symbiotic interaction	0.02984	2
GO: Biological Process	GO:0019049	evasion or tolerance of host defenses by virus	0.02984	2
GO: Biological Process	GO:0006433	prolyl-tRNA aminoacylation	0.02984	2
GO: Biological Process	GO:0044415	evasion or tolerance of host defenses	0.02984	2
GO: Biological Process	GO:0051832	avoidance of defenses of other organism involved in symbiotic interaction	0.02984	2
GO: Biological Process	GO:0090170	regulation of Golgi inheritance	0.0488	2
GO: Biological Process	GO:0006015	5-phosphoribose 1-diphosphate biosynthetic process	0.0488	2
GO: Biological Process	GO:0046391	5-phosphoribose 1-diphosphate metabolic process	0.0488	2
GO: Biological Process	GO:0070966	nuclear-transcribed mRNA catabolic process, no-go decay	0.0488	2
GO: Biological Process	GO:0032077	positive regulation of deoxyribonuclease activity	0.0488	2
GO: Biological Process	GO:0046083	adenine metabolic process	0.0488	2
GO: Biological Process	GO:0090365	regulation of mRNA modification	0.0488	2
GO: Biological Process	GO:0046084	adenine biosynthetic process	0.0488	2
GO: Biological Process	GO:0009176	pyrimidine deoxyribonucleoside monophosphate metabolic process	0.0488	2
GO: Biological Process	GO:0097167	circadian regulation of translation	0.0488	2
**Cellular Component**				
GO: Cellular Component	GO:1990904	ribonucleoprotein complex	4.289E-161	221
GO: Cellular Component	GO:0005730	nucleolus	4.492E-48	127
GO: Cellular Component	GO:0005840	ribosome	1.604E-118	121
GO: Cellular Component	GO:0044391	ribosomal subunit	1.087E-120	110
GO: Cellular Component	GO:0005739	mitochondrion	3.032E-10	106
GO: Cellular Component	GO:0044445	cytosolic part	2.792E-82	97
GO: Cellular Component	GO:0022626	cytosolic ribosome	4E-100	85
GO: Cellular Component	GO:0030054	cell junction	6.882E-11	83
GO: Cellular Component	GO:0044430	cytoskeletal part	0.0004784	74
GO: Cellular Component	GO:0031975	envelope	2.474E-07	72

GO: Cellular Component	GO:0031967	organelle envelope	4.238E-07	71
GO: Cellular Component	GO:0005694	chromosome	2.444E-09	67
GO: Cellular Component	GO:0044427	chromosomal part	5.968E-11	66
GO: Cellular Component	GO:0044429	mitochondrial part	3.964E-08	66
GO: Cellular Component	GO:0070161	anchoring junction	2.503E-21	65
GO: Cellular Component	GO:0005912	adherens junction	1.771E-21	64
GO: Cellular Component	GO:0005925	focal adhesion	1.161E-25	63
GO: Cellular Component	GO:0005924	cell-substrate adherens junction	2.199E-25	63
GO: Cellular Component	GO:0030055	cell-substrate junction	4.147E-25	63
GO: Cellular Component	GO:0015934	large ribosomal subunit	4.093E-61	60
GO: Cellular Component	GO:0015630	microtubule cytoskeleton	0.0003956	59
GO: Cellular Component	GO:0044451	nucleoplasm part	8.527E-10	58
GO: Cellular Component	GO:0043005	neuron projection	0.01045	55
GO: Cellular Component	GO:0019866	organelle inner membrane	1.253E-11	53
GO: Cellular Component	GO:0005740	mitochondrial envelope	1.358E-07	53
GO: Cellular Component	GO:0015935	small ribosomal subunit	5.203E-61	52
GO: Cellular Component	GO:0031966	mitochondrial membrane	5.08E-08	52
GO: Cellular Component	GO:1902494	catalytic complex	0.008851	51
GO: Cellular Component	GO:0022625	cytosolic large ribosomal subunit	6.66E-60	50
GO: Cellular Component	GO:0005759	mitochondrial matrix	2.128E-13	48
GO: Cellular Component	GO:0005743	mitochondrial inner membrane	1.145E-10	48
GO: Cellular Component	GO:0000785	chromatin	1.043E-08	43
GO: Cellular Component	GO:0000228	nuclear chromosome	7.835E-07	43
GO: Cellular Component	GO:0099513	polymeric cytoskeletal fiber	0.0002007	43
GO: Cellular Component	GO:0099512	supramolecular fiber	0.0002562	43
GO: Cellular Component	GO:0016604	nuclear body	4.516E-12	42
GO: Cellular Component	GO:0044454	nuclear chromosome part	3.365E-07	42
GO: Cellular Component	GO:0005681	spliceosomal complex	3.327E-22	40
GO: Cellular Component	GO:0036477	somatodendritic compartment	0.03335	40
GO: Cellular Component	GO:0035770	ribonucleoprotein granule	2.126E-21	37

GO: Cellular Component	GO:0044297	cell body	0.005395	37
GO: Cellular Component	GO:0048471	perinuclear region of cytoplasm	0.01045	37
GO: Cellular Component	GO:0022627	cytosolic small ribosomal subunit	1.375E-45	36
GO: Cellular Component	GO:1990234	transferase complex	0.04191	34
GO: Cellular Component	GO:0036464	cytoplasmic ribonucleoprotein granule	2.841E-17	32
GO: Cellular Component	GO:0005874	microtubule	0.00003644	32
GO: Cellular Component	GO:0016607	nuclear speck	3.461E-12	31
GO: Cellular Component	GO:0043209	myelin sheath	3.825E-12	31
GO: Cellular Component	GO:0030425	dendrite	0.01483	31
GO: Cellular Component	GO:0000790	nuclear chromatin	0.00003171	27
GO: Cellular Component	GO:0000313	organellar ribosome	1.216E-18	26
GO: Cellular Component	GO:0005761	mitochondrial ribosome	1.216E-18	26
GO: Cellular Component	GO:0071013	catalytic step 2 spliceosome	4.477E-17	26
GO: Cellular Component	GO:0098687	chromosomal region	0.0002329	26
GO: Cellular Component	GO:0005667	transcription factor complex	0.006731	23
GO: Cellular Component	GO:0032993	protein-DNA complex	0.00001086	20
GO: Cellular Component	GO:0034399	nuclear periphery	4.238E-07	19
GO: Cellular Component	GO:0016363	nuclear matrix	2.856E-07	17
GO: Cellular Component	GO:0000781	chromosome, telomeric region	0.0001044	17
GO: Cellular Component	GO:0005763	mitochondrial small ribosomal subunit	1.361E-14	15
GO: Cellular Component	GO:0000314	organellar small ribosomal subunit	1.361E-14	15
GO: Cellular Component	GO:0010494	cytoplasmic stress granule	5.229E-13	15
GO: Cellular Component	GO:0005844	polysome	2.125E-10	15
GO: Cellular Component	GO:0097525	spliceosomal snRNP complex	9.052E-10	15
GO: Cellular Component	GO:0030532	small nuclear ribonucleoprotein complex	5.599E-09	15
GO: Cellular Component	GO:0000784	nuclear chromosome, telomeric region	0.0001394	15
GO: Cellular Component	GO:0034708	methyltransferase complex	0.00004648	13
GO: Cellular Component	GO:0000786	nucleosome	0.0007171	12
GO: Cellular Component	GO:0044815	DNA packaging complex	0.001172	12
GO: Cellular Component	GO:0042470	melanosome	0.002681	11

GO: Cellular Component	GO:0048770	pigment granule	0.002681	11
GO: Cellular Component	GO:0044798	nuclear transcription factor complex	0.01483	11
GO: Cellular Component	GO:0005685	U1 snRNP	2.174E-10	10
GO: Cellular Component	GO:0005689	U12-type spliceosomal complex	3.673E-08	10
GO: Cellular Component	GO:0005684	U2-type spliceosomal complex	2.445E-07	10
GO: Cellular Component	GO:0030684	preribosome	0.0004332	10
GO: Cellular Component	GO:0005762	mitochondrial large ribosomal subunit	0.000014	9
GO: Cellular Component	GO:0000315	organellar large ribosomal subunit	0.000014	9
GO: Cellular Component	GO:0005884	actin filament	0.0129	9
GO: Cellular Component	GO:0030863	cortical cytoskeleton	0.01525	9
GO: Cellular Component	GO:0005832	chaperonin-containing T-complex	1.361E-10	8
GO: Cellular Component	GO:0002199	zona pellucida receptor complex	1.998E-09	8
GO: Cellular Component	GO:0005686	U2 snRNP	0.000000521	8
GO: Cellular Component	GO:0071011	precatalytic spliceosome	0.000002873	8
GO: Cellular Component	GO:0030687	preribosome, large subunit precursor	0.0000058	8
GO: Cellular Component	GO:0042645	mitochondrial nucleoid	0.000551	8
GO: Cellular Component	GO:0009295	nucleoid	0.0007376	8
GO: Cellular Component	GO:0072686	mitotic spindle	0.004751	8
GO: Cellular Component	GO:0044452	nucleolar part	0.007866	8
GO: Cellular Component	GO:0035097	histone methyltransferase complex	0.009817	8
GO: Cellular Component	GO:0034719	SMN-Sm protein complex	0.000004222	7
GO: Cellular Component	GO:0071010	prespliceosome	0.00002057	7
GO: Cellular Component	GO:0008180	COP9 signalosome	0.0005133	7
GO: Cellular Component	GO:0000791	euchromatin	0.0014	7
GO: Cellular Component	GO:0000932	P-body	0.02965	7
GO: Cellular Component	GO:0042382	paraspeckles	1.318E-08	6
GO: Cellular Component	GO:0071141	SMAD protein complex	2.856E-07	6
GO: Cellular Component	GO:0071004	U2-type prespliceosome	0.00004885	6
GO: Cellular Component	GO:0000178	exosome (RNase complex)	0.0002562	6
GO: Cellular Component	GO:0035145	exon-exon junction complex	0.0004332	6

GO: Cellular Component	GO:0005719	nuclear euchromatin	0.001839	6
GO: Cellular Component	GO:0031672	A band	0.01044	6
GO: Cellular Component	GO:1903293	phosphatase complex	0.0202	6
GO: Cellular Component	GO:0008287	protein serine/threonine phosphatase complex	0.0202	6
GO: Cellular Component	GO:0005871	kinesin complex	0.02975	6
GO: Cellular Component	GO:0070937	CRD-mediated mRNA stability complex	0.000001837	5
GO: Cellular Component	GO:0005687	U4 snRNP	0.00005664	5
GO: Cellular Component	GO:0034709	methylosome	0.0001599	5
GO: Cellular Component	GO:0000176	nuclear exosome (RNase complex)	0.0004945	5
GO: Cellular Component	GO:0005697	telomerase holoenzyme complex	0.001561	5
GO: Cellular Component	GO:0002102	podosome	0.006187	5
GO: Cellular Component	GO:0097526	spliceosomal tri-snRNP complex	0.008335	5
GO: Cellular Component	GO:0000788	nuclear nucleosome	0.03847	5
GO: Cellular Component	GO:0097452	GAIT complex	0.000009873	4
GO: Cellular Component	GO:0034715	pICln-Sm protein complex	0.0001156	4
GO: Cellular Component	GO:1990124	messenger ribonucleoprotein complex	0.001172	4
GO: Cellular Component	GO:0000177	cytoplasmic exosome (RNase complex)	0.005564	4
GO: Cellular Component	GO:0005682	U5 snRNP	0.006982	4
GO: Cellular Component	GO:0071682	endocytic vesicle lumen	0.008531	4
GO: Cellular Component	GO:0035327	transcriptionally active chromatin	0.01726	4
GO: Cellular Component	GO:0005753	mitochondrial proton-transporting ATP synthase complex	0.01726	4
GO: Cellular Component	GO:0045259	proton-transporting ATP synthase complex	0.02344	4
GO: Cellular Component	GO:0046540	U4/U6 x U5 tri-snRNP complex	0.02344	4
GO: Cellular Component	GO:0009328	phenylalanine-tRNA ligase complex	0.0002544	3
GO: Cellular Component	GO:0005853	eukaryotic translation elongation factor 1 complex	0.000833	3
GO: Cellular Component	GO:0070552	BRISC complex	0.001906	3
GO: Cellular Component	GO:0005786	signal recognition particle, endoplasmic reticulum targeting	0.003434	3
GO: Cellular Component	GO:0048500	signal recognition particle	0.003434	3
GO: Cellular Component	GO:0017101	aminoacyl-tRNA synthetase multienzyme complex	0.003434	3
GO: Cellular Component	GO:0000127	transcription factor TFIIIC complex	0.003434	3

GO: Cellular Component	GO:0000275	mitochondrial proton-transporting ATP synthase complex, catalytic core F(1)	0.003434	3
GO: Cellular Component	GO:0045261	proton-transporting ATP synthase complex, catalytic core F(1)	0.003434	3
GO: Cellular Component	GO:0072357	PTW/PP1 phosphatase complex	0.005564	3
GO: Cellular Component	GO:0000243	commitment complex	0.005564	3
GO: Cellular Component	GO:0005683	U7 snRNP	0.005564	3
GO: Cellular Component	GO:0090576	RNA polymerase III transcription factor complex	0.008151	3
GO: Cellular Component	GO:0072669	tRNA-splicing ligase complex	0.008151	3
GO: Cellular Component	GO:0001650	fibrillar center	0.01118	3
GO: Cellular Component	GO:0048188	Set1C/COMPASS complex	0.0194	3
GO: Cellular Component	GO:0000782	telomere cap complex	0.02947	3
GO: Cellular Component	GO:0031616	spindle pole centrosome	0.02947	3
GO: Cellular Component	GO:0032797	SMN complex	0.02947	3
GO: Cellular Component	GO:0000783	nuclear telomere cap complex	0.02947	3
GO: Cellular Component	GO:0031011	Ino80 complex	0.03568	3
GO: Cellular Component	GO:0033202	DNA helicase complex	0.04191	3
GO: Cellular Component	GO:0071144	heteromeric SMAD protein complex	0.005782	2
GO: Cellular Component	GO:0043564	Ku70:Ku80 complex	0.01414	2
GO: Cellular Component	GO:0032444	activin responsive factor complex	0.01414	2
GO: Cellular Component	GO:0044530	supraspliceosomal complex	0.01414	2
GO: Cellular Component	GO:0097524	sperm plasma membrane	0.02496	2
GO: Cellular Component	GO:0035061	interchromatin granule	0.02496	2
GO: Cellular Component	GO:0035985	senescence-associated heterochromatin focus	0.02496	2
GO: Cellular Component	GO:0002189	ribose phosphate diphosphokinase complex	0.02496	2
GO: Cellular Component	GO:0001651	dense fibrillar component	0.03783	2
GO: Cellular Component	GO:0045298	tubulin complex	0.03783	2
GO: Cellular Component	GO:0097255	R2TP complex	0.03783	2

Decreased in the presence of SMURF2 (wild-type and mutant Cys716Gly form)				
**Molecular Functions**				
GO: Molecular Function	GO:0003723	RNA binding	0.004098	14
GO: Molecular Function	GO:0042802	identical protein binding	0.0182	11
GO: Molecular Function	GO:0016491	oxidoreductase activity	0.04969	7
GO: Molecular Function	GO:0042623	ATPase activity, coupled	0.0382	5
GO: Molecular Function	GO:0000146	microfilament motor activity	0.0001103	4
GO: Molecular Function	GO:0051015	actin filament binding	0.0122	4
GO: Molecular Function	GO:0003774	motor activity	0.0122	4
GO: Molecular Function	GO:0005516	calmodulin binding	0.03655	4
GO: Molecular Function	GO:0030898	actin-dependent ATPase activity	0.0007356	3
GO: Molecular Function	GO:0034987	immunoglobulin receptor binding	0.004098	3
GO: Molecular Function	GO:0043531	ADP binding	0.006509	3
GO: Molecular Function	GO:0051087	chaperone binding	0.03343	3
GO: Molecular Function	GO:0003697	single-stranded DNA binding	0.04268	3
GO: Molecular Function	GO:0004738	pyruvate dehydrogenase activity	0.006189	2
GO: Molecular Function	GO:0034604	pyruvate dehydrogenase (NAD+) activity	0.006189	2
GO: Molecular Function	GO:0034603	pyruvate dehydrogenase [NAD(P)+] activity	0.006189	2
GO: Molecular Function	GO:0015130	mevalonate transmembrane transporter activity	0.04268	1
GO: Molecular Function	GO:0004148	dihydrolipoyl dehydrogenase activity	0.04268	1
GO: Molecular Function	GO:0043544	lipoamide binding	0.04268	1
GO: Molecular Function	GO:0004149	dihydrolipoyllysine-residue succinyltransferase activity	0.04268	1
**Biological processes**				
GO: Biological Process	GO:0044248	cellular catabolic process	0.02007	14
GO: Biological Process	GO:1901575	organic substance catabolic process	0.04769	13
GO: Biological Process	GO:0006508	proteolysis	0.04923	12
GO: Biological Process	GO:0006897	endocytosis	0.0124	9
GO: Biological Process	GO:0055114	oxidation-reduction process	0.04667	9

GO: Biological Process	GO:0006091	generation of precursor metabolites and energy	0.01262	7
GO: Biological Process	GO:0030048	actin filament-based movement	0.001584	6
GO: Biological Process	GO:0006909	phagocytosis	0.0124	6
GO: Biological Process	GO:0015980	energy derivation by oxidation of organic compounds	0.01262	6
GO: Biological Process	GO:0045333	cellular respiration	0.01262	5
GO: Biological Process	GO:0006898	receptor-mediated endocytosis	0.04923	5
GO: Biological Process	GO:0006958	complement activation, classical pathway	0.0124	4
GO: Biological Process	GO:0009060	aerobic respiration	0.0124	4
GO: Biological Process	GO:0002455	humoral immune response mediated by circulating immunoglobulin	0.0124	4
GO: Biological Process	GO:0006956	complement activation	0.01262	4
GO: Biological Process	GO:0072376	protein activation cascade	0.02007	4
GO: Biological Process	GO:0016064	immunoglobulin mediated immune response	0.03264	4
GO: Biological Process	GO:0019724	B cell mediated immunity	0.0331	4
GO: Biological Process	GO:0008360	regulation of cell shape	0.0331	4
GO: Biological Process	GO:0031032	actomyosin structure organization	0.03963	4
GO: Biological Process	GO:0046487	glyoxylate metabolic process	0.0124	3
GO: Biological Process	GO:0006099	tricarboxylic acid cycle	0.0124	3
GO: Biological Process	GO:0006101	citrate metabolic process	0.01262	3
GO: Biological Process	GO:0006084	acetyl-CoA metabolic process	0.01262	3
GO: Biological Process	GO:0072350	tricarboxylic acid metabolic process	0.0144	3
GO: Biological Process	GO:0006911	phagocytosis, engulfment	0.03121	3
GO: Biological Process	GO:0016441	posttranscriptional gene silencing	0.03136	3
GO: Biological Process	GO:0035194	posttranscriptional gene silencing by RNA	0.03136	3
GO: Biological Process	GO:0010324	membrane invagination	0.0331	3
GO: Biological Process	GO:0061732	mitochondrial acetyl-CoA biosynthetic process from pyruvate	0.0144	2
GO: Biological Process	GO:0060211	regulation of nuclear-transcribed mRNA poly(A) tail shortening	0.02996	2
GO: Biological Process	GO:0060213	positive regulation of nuclear-transcribed mRNA poly(A) tail shortening	0.02996	2
GO: Biological Process	GO:0010510	regulation of acetyl-CoA biosynthetic process from pyruvate	0.03121	2
GO: Biological Process	GO:0006554	lysine catabolic process	0.03121	2
GO: Biological Process	GO:0050812	regulation of acyl-CoA biosynthetic process	0.03136	2

GO: Biological Process	GO:0006553	lysine metabolic process	0.03136	2
GO: Biological Process	GO:0006086	acetyl-CoA biosynthetic process from pyruvate	0.0331	2
GO: Biological Process	GO:1900151	regulation of nuclear-transcribed mRNA catabolic process, deadenylation-dependent decay	0.0331	2
GO: Biological Process	GO:1900153	positive regulation of nuclear-transcribed mRNA catabolic process, deadenylation-dependent decay	0.0331	2
GO: Biological Process	GO:0033197	response to vitamin E	0.0331	2
GO: Biological Process	GO:0031442	positive regulation of mRNA 3'-end processing	0.03963	2
GO: Biological Process	GO:0006085	acetyl-CoA biosynthetic process	0.04667	2
GO: Biological Process	GO:0009068	aspartate family amino acid catabolic process	0.04667	2
GO: Biological Process	GO:0070584	mitochondrion morphogenesis	0.04852	2
GO: Biological Process	GO:0006103	2-oxoglutarate metabolic process	0.04923	2
GO: Biological Process	GO:0042762	regulation of sulfur metabolic process	0.04923	2
**Cellular Component**				
GO: Cellular Component	GO:0005739	mitochondrion	0.0221	12
GO: Cellular Component	GO:0044430	cytoskeletal part	0.04738	10
GO: Cellular Component	GO:0044429	mitochondrial part	0.0406	8
GO: Cellular Component	GO:1902494	catalytic complex	0.04878	8
GO: Cellular Component	GO:0015629	actin cytoskeleton	0.02023	6
GO: Cellular Component	GO:0070161	anchoring junction	0.02389	6
GO: Cellular Component	GO:0016460	myosin II complex	0.00000204	5
GO: Cellular Component	GO:0016459	myosin complex	0.00006074	5
GO: Cellular Component	GO:0043209	myelin sheath	0.003753	5
GO: Cellular Component	GO:0005911	cell-cell junction	0.04683	5
GO: Cellular Component	GO:0005759	mitochondrial matrix	0.04716	5
GO: Cellular Component	GO:0032982	myosin filament	0.0000311	4
GO: Cellular Component	GO:0001725	stress fiber	0.001081	4
GO: Cellular Component	GO:0097517	contractile actin filament bundle	0.001081	4
GO: Cellular Component	GO:0032432	actin filament bundle	0.001285	4
GO: Cellular Component	GO:0042641	actomyosin	0.001285	4
GO: Cellular Component	GO:0005903	brush border	0.006305	4

GO: Cellular Component	GO:0072562	blood microparticle	0.008216	4
GO: Cellular Component	GO:0098862	cluster of actin-based cell projections	0.01649	4
GO: Cellular Component	GO:0000151	ubiquitin ligase complex	0.04822	4
GO: Cellular Component	GO:0097513	myosin II filament	0.000002222	3
GO: Cellular Component	GO:0042571	immunoglobulin complex, circulating	0.001081	3
GO: Cellular Component	GO:0019814	immunoglobulin complex	0.001285	3
GO: Cellular Component	GO:1990204	oxidoreductase complex	0.02389	3
GO: Cellular Component	GO:0042579	microbody	0.04859	3
GO: Cellular Component	GO:0005777	peroxisome	0.04859	3
GO: Cellular Component	GO:0036464	cytoplasmic ribonucleoprotein granule	0.04878	3
GO: Cellular Component	GO:0035770	ribonucleoprotein granule	0.04878	3
GO: Cellular Component	GO:0045252	oxoglutarate dehydrogenase complex	0.002384	2
GO: Cellular Component	GO:0045254	pyruvate dehydrogenase complex	0.003753	2
GO: Cellular Component	GO:0045240	dihydrolipoyl dehydrogenase complex	0.005582	2
GO: Cellular Component	GO:0045239	tricarboxylic acid cycle enzyme complex	0.008216	2
GO: Cellular Component	GO:0030057	desmosome	0.02389	2
GO: Cellular Component	GO:0008180	COP9 signalosome	0.03899	2
GO: Cellular Component	GO:0097610	cell surface furrow	0.04878	2
GO: Cellular Component	GO:0032154	cleavage furrow	0.04878	2
GO: Cellular Component	GO:0005584	collagen type I trimer	0.04716	1
GO: Cellular Component	GO:0070436	Grb2-EGFR complex	0.04716	1
GO: Cellular Component	GO:0071756	pentameric IgM immunoglobulin complex	0.04878	1
GO: Cellular Component	GO:0071754	IgM immunoglobulin complex, circulating	0.04878	1
GO: Cellular Component	GO:0043159	acrosomal matrix	0.04878	1
GO: Cellular Component	GO:0071753	IgM immunoglobulin complex	0.04878	1

Table S2. Details of GO terms for SMURF2WT and SMURF2CG

Increased in the presence of SMURF2WT				
**Category**	**ID**	**Name**	**q-value FDR B&H**	**Hit Count in Query List**
**Molecular Functions**				
GO: Molecular Function	GO:0003723	RNA binding	9.158E-33	62
GO: Molecular Function	GO:0005198	structural molecule activity	0.0006967	17
GO: Molecular Function	GO:0003735	structural constituent of ribosome	0.000002297	12
GO: Molecular Function	GO:0051082	unfolded protein binding	0.003506	6
GO: Molecular Function	GO:0003729	mRNA binding	0.03022	6
GO: Molecular Function	GO:0003725	double-stranded RNA binding	0.03022	4
GO: Molecular Function	GO:0032552	deoxyribonucleotide binding	0.001554	3
GO: Molecular Function	GO:0017091	AU-rich element binding	0.02297	3
GO: Molecular Function	GO:0004532	exoribonuclease activity	0.0466	3
GO: Molecular Function	GO:0032556	pyrimidine deoxyribonucleotide binding	0.003506	2
GO: Molecular Function	GO:0004827	proline-tRNA ligase activity	0.008003	2
GO: Molecular Function	GO:0034604	pyruvate dehydrogenase (NAD+) activity	0.03022	2
GO: Molecular Function	GO:0034603	pyruvate dehydrogenase [NAD(P)+] activity	0.03022	2
GO: Molecular Function	GO:1990247	N6-methyladenosine-containing RNA binding	0.03022	2
GO: Molecular Function	GO:0004738	pyruvate dehydrogenase activity	0.03022	2
**Biological Processes**				
GO: Biological Process	GO:0006396	RNA processing	3.698E-08	26
GO: Biological Process	GO:1901575	organic substance catabolic process	0.003539	26
GO: Biological Process	GO:1901566	organonitrogen compound biosynthetic process	0.0001414	25
GO: Biological Process	GO:0016071	mRNA metabolic process	3.412E-09	24
GO: Biological Process	GO:0065003	protein-containing complex assembly	0.009019	24
GO: Biological Process	GO:0044248	cellular catabolic process	0.009019	24
GO: Biological Process	GO:0043603	cellular amide metabolic process	0.00008153	21

GO: Biological Process	GO:0006412	translation	0.000001342	20
GO: Biological Process	GO:0043043	peptide biosynthetic process	0.000002046	20
GO: Biological Process	GO:0043604	amide biosynthetic process	0.000007414	20
GO: Biological Process	GO:0006518	peptide metabolic process	0.0000217	20
GO: Biological Process	GO:0051276	chromosome organization	0.01109	18
GO: Biological Process	GO:0022613	ribonucleoprotein complex biogenesis	0.000001342	17
GO: Biological Process	GO:0034655	nucleobase-containing compound catabolic process	0.000001648	16
GO: Biological Process	GO:0044270	cellular nitrogen compound catabolic process	0.000004172	16
GO: Biological Process	GO:0046700	heterocycle catabolic process	0.000004172	16
GO: Biological Process	GO:0019439	aromatic compound catabolic process	0.00000518	16
GO: Biological Process	GO:1901361	organic cyclic compound catabolic process	0.00001104	16
GO: Biological Process	GO:0044265	cellular macromolecule catabolic process	0.01061	16
GO: Biological Process	GO:0006397	mRNA processing	0.00002479	15
GO: Biological Process	GO:0034660	ncRNA metabolic process	0.0001454	15
GO: Biological Process	GO:0008380	RNA splicing	0.00001993	14
GO: Biological Process	GO:0007005	mitochondrion organization	0.009019	14
GO: Biological Process	GO:0042254	ribosome biogenesis	0.00001104	13
GO: Biological Process	GO:0022411	cellular component disassembly	0.008144	13
GO: Biological Process	GO:0045087	innate immune response	0.0373	13
GO: Biological Process	GO:0000398	mRNA splicing, via spliceosome	0.00003633	12
GO: Biological Process	GO:0000377	RNA splicing, via transesterification reactions with bulged adenosine as nucleophile	0.00003633	12
GO: Biological Process	GO:0000375	RNA splicing, via transesterification reactions	0.00003993	12
GO: Biological Process	GO:0051259	protein complex oligomerization	0.004668	12
GO: Biological Process	GO:0006325	chromatin organization	0.04562	12
GO: Biological Process	GO:0034470	ncRNA processing	0.002075	11
GO: Biological Process	GO:0006414	translational elongation	0.000002049	10
GO: Biological Process	GO:0006401	RNA catabolic process	0.0002263	10
GO: Biological Process	GO:0006364	rRNA processing	0.0003325	10
GO: Biological Process	GO:0016072	rRNA metabolic process	0.0004064	10
GO: Biological Process	GO:0032984	protein-containing complex disassembly	0.0005266	10

GO: Biological Process	GO:0016570	histone modification	0.0139	10
GO: Biological Process	GO:0016569	covalent chromatin modification	0.01607	10
GO: Biological Process	GO:0010608	posttranscriptional regulation of gene expression	0.01905	10
GO: Biological Process	GO:0070125	mitochondrial translational elongation	0.000001342	9
GO: Biological Process	GO:0070126	mitochondrial translational termination	0.000001342	9
GO: Biological Process	GO:0006415	translational termination	0.000002049	9
GO: Biological Process	GO:0032543	mitochondrial translation	0.000007414	9
GO: Biological Process	GO:0000956	nuclear-transcribed mRNA catabolic process	0.0003224	9
GO: Biological Process	GO:0043624	cellular protein complex disassembly	0.000448	9
GO: Biological Process	GO:0006402	mRNA catabolic process	0.0005235	9
GO: Biological Process	GO:0033044	regulation of chromosome organization	0.004366	9
GO: Biological Process	GO:0006403	RNA localization	0.002698	8
GO: Biological Process	GO:0009266	response to temperature stimulus	0.004386	8
GO: Biological Process	GO:0051262	protein tetramerization	0.002362	7
GO: Biological Process	GO:0009408	response to heat	0.004082	7
GO: Biological Process	GO:0000184	nuclear-transcribed mRNA catabolic process, nonsense-mediated decay	0.004366	6
GO: Biological Process	GO:2001252	positive regulation of chromosome organization	0.0135	6
GO: Biological Process	GO:0006399	tRNA metabolic process	0.02125	6
GO: Biological Process	GO:0006413	translational initiation	0.02809	6
GO: Biological Process	GO:0071229	cellular response to acid chemical	0.03342	6
GO: Biological Process	GO:0006369	termination of RNA polymerase II transcription	0.002291	5
GO: Biological Process	GO:0051289	protein homotetramerization	0.003775	5
GO: Biological Process	GO:0006353	DNA-templated transcription, termination	0.01366	5
GO: Biological Process	GO:0031123	RNA 3'-end processing	0.02132	5
GO: Biological Process	GO:0071346	cellular response to interferon-gamma	0.03342	5
GO: Biological Process	GO:0000723	telomere maintenance	0.03342	5
GO: Biological Process	GO:0031056	regulation of histone modification	0.03727	5
GO: Biological Process	GO:0032200	telomere organization	0.04436	5
GO: Biological Process	GO:0008334	histone mRNA metabolic process	0.001532	4
GO: Biological Process	GO:0000460	maturation of 5.8S rRNA	0.002362	4

GO: Biological Process	GO:0000387	spliceosomal snRNP assembly	0.004226	4
GO: Biological Process	GO:0007004	telomere maintenance via telomerase	0.0132	4
GO: Biological Process	GO:0006278	RNA-dependent DNA biosynthetic process	0.01905	4
GO: Biological Process	GO:0010833	telomere maintenance via telomere lengthening	0.0197	4
GO: Biological Process	GO:0032204	regulation of telomere maintenance	0.02043	4
GO: Biological Process	GO:1900034	regulation of cellular response to heat	0.02809	4
GO: Biological Process	GO:0060333	interferon-gamma-mediated signaling pathway	0.03549	4
GO: Biological Process	GO:0031058	positive regulation of histone modification	0.04214	4
GO: Biological Process	GO:0009124	nucleoside monophosphate biosynthetic process	0.04562	4
GO: Biological Process	GO:0006614	SRP-dependent cotranslational protein targeting to membrane	0.04562	4
GO: Biological Process	GO:0071028	nuclear mRNA surveillance	0.002525	3
GO: Biological Process	GO:0071027	nuclear RNA surveillance	0.002525	3
GO: Biological Process	GO:0009143	nucleoside triphosphate catabolic process	0.003112	3
GO: Biological Process	GO:0071025	RNA surveillance	0.003764	3
GO: Biological Process	GO:0016075	rRNA catabolic process	0.006753	3
GO: Biological Process	GO:0034661	ncRNA catabolic process	0.01509	3
GO: Biological Process	GO:0070198	protein localization to chromosome, telomeric region	0.01697	3
GO: Biological Process	GO:0009394	2'-deoxyribonucleotide metabolic process	0.02736	3
GO: Biological Process	GO:0090503	RNA phosphodiester bond hydrolysis, exonucleolytic	0.02809	3
GO: Biological Process	GO:0019692	deoxyribose phosphate metabolic process	0.02916	3
GO: Biological Process	GO:0042769	DNA damage response, detection of DNA damage	0.02916	3
GO: Biological Process	GO:0009262	deoxyribonucleotide metabolic process	0.03381	3
GO: Biological Process	GO:0032210	regulation of telomere maintenance via telomerase	0.04562	3
GO: Biological Process	GO:0009177	pyrimidine deoxyribonucleoside monophosphate biosynthetic process	0.004566	2
GO: Biological Process	GO:0006433	prolyl-tRNA aminoacylation	0.004566	2
GO: Biological Process	GO:0009176	pyrimidine deoxyribonucleoside monophosphate metabolic process	0.008445	2
GO: Biological Process	GO:0071029	nuclear ncRNA surveillance	0.01607	2
GO: Biological Process	GO:0043634	polyadenylation-dependent ncRNA catabolic process	0.01607	2
GO: Biological Process	GO:0042791	5S class rRNA transcription by RNA polymerase III	0.01607	2
GO: Biological Process	GO:0071035	nuclear polyadenylation-dependent rRNA catabolic process	0.01607	2

GO: Biological Process	GO:0042797	tRNA transcription by RNA polymerase III	0.01607	2
GO: Biological Process	GO:0071046	nuclear polyadenylation-dependent ncRNA catabolic process	0.01607	2
GO: Biological Process	GO:0009221	pyrimidine deoxyribonucleotide biosynthetic process	0.0197	2
GO: Biological Process	GO:0009204	deoxyribonucleoside triphosphate catabolic process	0.0197	2
GO: Biological Process	GO:0043633	polyadenylation-dependent RNA catabolic process	0.0197	2
GO: Biological Process	GO:0009157	deoxyribonucleoside monophosphate biosynthetic process	0.0197	2
GO: Biological Process	GO:0061732	mitochondrial acetyl-CoA biosynthetic process from pyruvate	0.0197	2
GO: Biological Process	GO:0009304	tRNA transcription	0.0197	2
GO: Biological Process	GO:0034475	U4 snRNA 3'-end processing	0.02413	2
GO: Biological Process	GO:1904869	regulation of protein localization to Cajal body	0.02413	2
GO: Biological Process	GO:1904871	positive regulation of protein localization to Cajal body	0.02413	2
GO: Biological Process	GO:1901897	regulation of relaxation of cardiac muscle	0.02413	2
GO: Biological Process	GO:1904867	protein localization to Cajal body	0.02809	2
GO: Biological Process	GO:1990173	protein localization to nucleoplasm	0.02809	2
GO: Biological Process	GO:1904851	positive regulation of establishment of protein localization to telomere	0.02809	2
GO: Biological Process	GO:0034427	nuclear-transcribed mRNA catabolic process, exonucleolytic, 3'-5'	0.02809	2
GO: Biological Process	GO:1903405	protein localization to nuclear body	0.02809	2
GO: Biological Process	GO:0009162	deoxyribonucleoside monophosphate metabolic process	0.03267	2
GO: Biological Process	GO:0070203	regulation of establishment of protein localization to telomere	0.03267	2
GO: Biological Process	GO:0009265	2'-deoxyribonucleotide biosynthetic process	0.03267	2
GO: Biological Process	GO:0034472	snRNA 3'-end processing	0.03267	2
GO: Biological Process	GO:0046385	deoxyribose phosphate biosynthetic process	0.03267	2
GO: Biological Process	GO:1901077	regulation of relaxation of muscle	0.03727	2
GO: Biological Process	GO:0070202	regulation of establishment of protein localization to chromosome	0.03727	2
GO: Biological Process	GO:0010510	regulation of acetyl-CoA biosynthetic process from pyruvate	0.04214	2
GO: Biological Process	GO:0009113	purine nucleobase biosynthetic process	0.04214	2
GO: Biological Process	GO:1904816	positive regulation of protein localization to chromosome, telomeric region	0.04214	2
GO: Biological Process	GO:0051085	chaperone cofactor-dependent protein refolding	0.04214	2
GO: Biological Process	GO:0009263	deoxyribonucleotide biosynthetic process	0.04562	2
GO: Biological Process	GO:0031125	rRNA 3'-end processing	0.04562	2

GO: Biological Process	GO:0050812	regulation of acyl-CoA biosynthetic process	0.04562	2
GO: Biological Process	GO:0000244	spliceosomal tri-snRNP complex assembly	0.04562	2
GO: Biological Process	GO:0009130	pyrimidine nucleoside monophosphate biosynthetic process	0.04562	2
**Cellular Components**				
GO: Cellular Component	GO:1990904	ribonucleoprotein complex	1.748E-18	34
GO: Cellular Component	GO:0005730	nucleolus	5.593E-08	24
GO: Cellular Component	GO:0005840	ribosome	1.889E-09	15
GO: Cellular Component	GO:0044429	mitochondrial part	0.01025	15
GO: Cellular Component	GO:0005759	mitochondrial matrix	0.00006934	13
GO: Cellular Component	GO:0044451	nucleoplasm part	0.007366	13
GO: Cellular Component	GO:0044391	ribosomal subunit	7.057E-07	11
GO: Cellular Component	GO:0005743	mitochondrial inner membrane	0.005224	11
GO: Cellular Component	GO:0019866	organelle inner membrane	0.007771	11
GO: Cellular Component	GO:0031966	mitochondrial membrane	0.02521	11
GO: Cellular Component	GO:0005740	mitochondrial envelope	0.0348	11
GO: Cellular Component	GO:0000313	organellar ribosome	0.000001329	8
GO: Cellular Component	GO:0005761	mitochondrial ribosome	0.000001329	8
GO: Cellular Component	GO:0015935	small ribosomal subunit	0.00001172	7
GO: Cellular Component	GO:0005681	spliceosomal complex	0.002258	7
GO: Cellular Component	GO:0044445	cytosolic part	0.007438	7
GO: Cellular Component	GO:0005667	transcription factor complex	0.0414	7
GO: Cellular Component	GO:0016607	nuclear speck	0.01339	6
GO: Cellular Component	GO:0071013	catalytic step 2 spliceosome	0.004861	5
GO: Cellular Component	GO:0016363	nuclear matrix	0.007366	5
GO: Cellular Component	GO:0034399	nuclear periphery	0.01339	5
GO: Cellular Component	GO:0005763	mitochondrial small ribosomal subunit	0.000732	4
GO: Cellular Component	GO:0000314	organellar small ribosomal subunit	0.000732	4
GO: Cellular Component	GO:0097525	spliceosomal snRNP complex	0.005894	4
GO: Cellular Component	GO:0030532	small nuclear ribonucleoprotein complex	0.007366	4
GO: Cellular Component	GO:0034708	methyltransferase complex	0.0198	4

GO: Cellular Component	GO:0015934	large ribosomal subunit	0.0285	4
GO: Cellular Component	GO:0022626	cytosolic ribosome	0.03785	4
GO: Cellular Component	GO:0034709	methylosome	0.001308	3
GO: Cellular Component	GO:0000176	nuclear exosome (RNase complex)	0.002258	3
GO: Cellular Component	GO:0000178	exosome (RNase complex)	0.005224	3
GO: Cellular Component	GO:0005689	U12-type spliceosomal complex	0.007366	3
GO: Cellular Component	GO:0005762	mitochondrial large ribosomal subunit	0.01339	3
GO: Cellular Component	GO:0000315	organellar large ribosomal subunit	0.01339	3
GO: Cellular Component	GO:0022627	cytosolic small ribosomal subunit	0.0198	3
GO: Cellular Component	GO:0034715	pICln-Sm protein complex	0.007366	2
GO: Cellular Component	GO:0000127	transcription factor TFIIIC complex	0.007366	2
GO: Cellular Component	GO:0005683	U7 snRNP	0.009087	2
GO: Cellular Component	GO:0090576	RNA polymerase III transcription factor complex	0.01082	2
GO: Cellular Component	GO:0045254	pyruvate dehydrogenase complex	0.01082	2
GO: Cellular Component	GO:0005832	chaperonin-containing T-complex	0.01339	2
GO: Cellular Component	GO:0005687	U4 snRNP	0.01429	2
GO: Cellular Component	GO:0002199	zona pellucida receptor complex	0.01692	2
GO: Cellular Component	GO:0000346	transcription export complex	0.02197	2
GO: Cellular Component	GO:0000177	cytoplasmic exosome (RNase complex)	0.02792	2
GO: Cellular Component	GO:0034719	SMN-Sm protein complex	0.03343	2
GO: Cellular Component	GO:0005685	U1 snRNP	0.03343	2
GO: Cellular Component	GO:0005686	U2 snRNP	0.03785	2
GO: Cellular Component	GO:0005697	telomerase holoenzyme complex	0.03785	2
GO: Cellular Component	GO:0035327	transcriptionally active chromatin	0.0414	2
GO: Cellular Component	GO:0045120	pronucleus	0.0414	2
GO: Cellular Component	GO:0046540	U4/U6 x U5 tri-snRNP complex	0.04772	2
GO: Cellular Component	GO:0071011	precatalytic spliceosome	0.04772	2
GO: Cellular Component	GO:0005691	U6atac snRNP	0.03785	1
GO: Cellular Component	GO:0071002	U4atac/U6atac snRNP	0.03785	1
GO: Cellular Component	GO:1990826	nucleoplasmic periphery of the nuclear pore complex	0.03785	1

Decreased in the presence of SMURF2WT				
Category	ID	Name	q-value FDR B&H	Hit Count in Query List
Molecular Functions				
GO: Molecular Function	GO:0015183	L-aspartate transmembrane transporter activity	0.01054	1
GO: Molecular Function	GO:0070191	methionine-R-sulfoxide reductase activity	0.01054	1
GO: Molecular Function	GO:0033743	peptide-methionine (R)-S-oxide reductase activity	0.01054	1
GO: Molecular Function	GO:0015556	C4-dicarboxylate transmembrane transporter activity	0.01054	1
GO: Molecular Function	GO:0008113	peptide-methionine (S)-S-oxide reductase activity	0.01054	1
GO: Molecular Function	GO:0005313	L-glutamate transmembrane transporter activity	0.01098	1
GO: Molecular Function	GO:0051787	misfolded protein binding	0.01098	1
GO: Molecular Function	GO:0015172	acidic amino acid transmembrane transporter activity	0.01098	1
GO: Molecular Function	GO:0016671	oxidoreductase activity, acting on a sulfur group of donors, disulfide as acceptor	0.01098	1
GO: Molecular Function	GO:0001671	ATPase activator activity	0.01185	1
GO: Molecular Function	GO:0015035	protein disulfide oxidoreductase activity	0.01436	1
GO: Molecular Function	GO:0005310	dicarboxylic acid transmembrane transporter activity	0.01491	1
GO: Molecular Function	GO:0060590	ATPase regulator activity	0.01491	1
GO: Molecular Function	GO:0015036	disulfide oxidoreductase activity	0.01491	1
GO: Molecular Function	GO:0030544	Hsp70 protein binding	0.01491	1
GO: Molecular Function	GO:0016667	oxidoreductase activity, acting on a sulfur group of donors	0.02126	1
GO: Molecular Function	GO:0015179	L-amino acid transmembrane transporter activity	0.02126	1
GO: Molecular Function	GO:0015171	amino acid transmembrane transporter activity	0.02832	1
GO: Molecular Function	GO:0051087	chaperone binding	0.02832	1
GO: Molecular Function	GO:0051117	ATPase binding	0.02854	1
GO: Molecular Function	GO:0003697	single-stranded DNA binding	0.03158	1
GO: Molecular Function	GO:0031072	heat shock protein binding	0.03158	1
GO: Molecular Function	GO:0046943	carboxylic acid transmembrane transporter activity	0.03925	1

GO: Molecular Function	GO:0005342	organic acid transmembrane transporter activity	0.03925	1
GO: Molecular Function	GO:0008514	organic anion transmembrane transporter activity	0.04778	1
**Biological Processes**				
GO: Biological Process	GO:0043490	malate-aspartate shuttle	0.04339	1
GO: Biological Process	GO:0070778	L-aspartate transmembrane transport	0.04339	1
GO: Biological Process	GO:0051096	positive regulation of helicase activity	0.04339	1
GO: Biological Process	GO:0051095	regulation of helicase activity	0.04339	1
GO: Biological Process	GO:0034975	protein folding in endoplasmic reticulum	0.04339	1
GO: Biological Process	GO:0015810	aspartate transmembrane transport	0.04339	1
GO: Biological Process	GO:0015740	C4-dicarboxylate transport	0.04395	1
**Cellular Components**				
GO: Cellular Component	GO:0044429	mitochondrial part	0.03298	2
GO: Cellular Component	GO:0034663	endoplasmic reticulum chaperone complex	0.03209	1
GO: Cellular Component	GO:0042645	mitochondrial nucleoid	0.03298	1
GO: Cellular Component	GO:0009295	nucleoid	0.03298	1

**Increased in the presence of SMURF2CG**				
**Category**	**ID**	**Name**	**q-value FDR B&H**	**Hit Count in Query List**
**Molecular Functions**				
GO: Molecular Function	GO:0003723	RNA binding	3.11E-28	58
GO: Molecular Function	GO:0042802	identical protein binding	0.001063	23
GO: Molecular Function	GO:0030554	adenyl nucleotide binding	0.01139	22
GO: Molecular Function	GO:0044877	protein-containing complex binding	0.03523	21
GO: Molecular Function	GO:0046983	protein dimerization activity	0.01139	20
GO: Molecular Function	GO:0005524	ATP binding	0.03421	20
GO: Molecular Function	GO:0032559	adenyl ribonucleotide binding	0.03523	20
GO: Molecular Function	GO:0005198	structural molecule activity	0.0007496	17
GO: Molecular Function	GO:0008092	cytoskeletal protein binding	0.03558	14

GO: Molecular Function	GO:0003735	structural constituent of ribosome	2.135E-07	13
GO: Molecular Function	GO:0017111	nucleoside-triphosphatase activity	0.03558	13
GO: Molecular Function	GO:0016462	pyrophosphatase activity	0.04873	13
GO: Molecular Function	GO:0016818	hydrolase activity, acting on acid anhydrides, in phosphorus-containing anhydrides	0.04873	13
GO: Molecular Function	GO:0016817	hydrolase activity, acting on acid anhydrides	0.04873	13
GO: Molecular Function	GO:0016887	ATPase activity	0.002074	12
GO: Molecular Function	GO:0046982	protein heterodimerization activity	0.03421	11
GO: Molecular Function	GO:0019843	rRNA binding	0.03421	4
GO: Molecular Function	GO:0004045	aminoacyl-tRNA hydrolase activity	0.02247	2
GO: Molecular Function	GO:0036402	proteasome-activating ATPase activity	0.02984	2
**Biological Processes**				
GO: Biological Process	GO:0065003	protein-containing complex assembly	0.000000395	34
GO: Biological Process	GO:1901566	organonitrogen compound biosynthetic process	3.448E-08	32
GO: Biological Process	GO:0043603	cellular amide metabolic process	7.921E-09	28
GO: Biological Process	GO:0043604	amide biosynthetic process	3.346E-10	27
GO: Biological Process	GO:0006518	peptide metabolic process	4.85E-09	26
GO: Biological Process	GO:0006412	translation	3.346E-10	25
GO: Biological Process	GO:0043043	peptide biosynthetic process	5.733E-10	25
GO: Biological Process	GO:0006396	RNA processing	5.384E-08	25
GO: Biological Process	GO:0034622	cellular protein-containing complex assembly	2.486E-07	25
GO: Biological Process	GO:0051276	chromosome organization	0.0002516	22
GO: Biological Process	GO:0009890	negative regulation of biosynthetic process	0.03419	21
GO: Biological Process	GO:0010558	negative regulation of macromolecule biosynthetic process	0.0372	20
GO: Biological Process	GO:0016071	mRNA metabolic process	0.00001725	18
GO: Biological Process	GO:0006325	chromatin organization	0.0001171	18
GO: Biological Process	GO:0022613	ribonucleoprotein complex biogenesis	6.904E-07	17
GO: Biological Process	GO:0022411	cellular component disassembly	0.0002516	16
GO: Biological Process	GO:0007005	mitochondrion organization	0.00123	16
GO: Biological Process	GO:0034660	ncRNA metabolic process	0.0001824	15
GO: Biological Process	GO:0032984	protein-containing complex disassembly	0.000002452	13

GO: Biological Process	GO:0008380	RNA splicing	0.0001214	13
GO: Biological Process	GO:0006397	mRNA processing	0.0005605	13
GO: Biological Process	GO:0006414	translational elongation	1.535E-08	12
GO: Biological Process	GO:0043624	cellular protein complex disassembly	0.000001209	12
GO: Biological Process	GO:0000398	mRNA splicing, via spliceosome	0.00004932	12
GO: Biological Process	GO:0000377	RNA splicing, via transesterification reactions with bulged adenosine as nucleophile	0.00004932	12
GO: Biological Process	GO:0000375	RNA splicing, via transesterification reactions	0.00005396	12
GO: Biological Process	GO:0032543	mitochondrial translation	4.848E-08	11
GO: Biological Process	GO:0042254	ribosome biogenesis	0.0003754	11
GO: Biological Process	GO:0034470	ncRNA processing	0.002104	11
GO: Biological Process	GO:0010608	posttranscriptional regulation of gene expression	0.008489	11
GO: Biological Process	GO:0070125	mitochondrial translational elongation	3.448E-08	10
GO: Biological Process	GO:0070126	mitochondrial translational termination	3.45E-08	10
GO: Biological Process	GO:0006415	translational termination	1.065E-07	10
GO: Biological Process	GO:0065004	protein-DNA complex assembly	0.0001284	10
GO: Biological Process	GO:0071824	protein-DNA complex subunit organization	0.0002861	10
GO: Biological Process	GO:0031497	chromatin assembly	0.00007965	9
GO: Biological Process	GO:0006333	chromatin assembly or disassembly	0.0002238	9
GO: Biological Process	GO:0006323	DNA packaging	0.0003868	9
GO: Biological Process	GO:0022618	ribonucleoprotein complex assembly	0.0004061	9
GO: Biological Process	GO:0071826	ribonucleoprotein complex subunit organization	0.0005605	9
GO: Biological Process	GO:0006364	rRNA processing	0.002041	9
GO: Biological Process	GO:0016072	rRNA metabolic process	0.002335	9
GO: Biological Process	GO:0071103	DNA conformation change	0.003776	9
GO: Biological Process	GO:0034655	nucleobase-containing compound catabolic process	0.0372	9
GO: Biological Process	GO:0006334	nucleosome assembly	0.0002516	8
GO: Biological Process	GO:0034728	nucleosome organization	0.0007141	8
GO: Biological Process	GO:0009167	purine ribonucleoside monophosphate metabolic process	0.01541	8
GO: Biological Process	GO:0009126	purine nucleoside monophosphate metabolic process	0.01549	8
GO: Biological Process	GO:0009161	ribonucleoside monophosphate metabolic process	0.0193	8

GO: Biological Process	GO:0009123	nucleoside monophosphate metabolic process	0.02362	8
GO: Biological Process	GO:0006417	regulation of translation	0.04234	8
GO: Biological Process	GO:1900182	positive regulation of protein localization to nucleus	0.001458	7
GO: Biological Process	GO:1900180	regulation of protein localization to nucleus	0.02489	7
GO: Biological Process	GO:0050821	protein stabilization	0.01184	6
GO: Biological Process	GO:0000956	nuclear-transcribed mRNA catabolic process	0.04666	6
GO: Biological Process	GO:0009168	purine ribonucleoside monophosphate biosynthetic process	0.004817	5
GO: Biological Process	GO:0009127	purine nucleoside monophosphate biosynthetic process	0.004817	5
GO: Biological Process	GO:0009156	ribonucleoside monophosphate biosynthetic process	0.009231	5
GO: Biological Process	GO:0009124	nucleoside monophosphate biosynthetic process	0.01345	5
GO: Biological Process	GO:0000184	nuclear-transcribed mRNA catabolic process, nonsense-mediated decay	0.03314	5
GO: Biological Process	GO:0000183	chromatin silencing at rDNA	0.005855	4
GO: Biological Process	GO:0042274	ribosomal small subunit biogenesis	0.02661	4
GO: Biological Process	GO:0045815	positive regulation of gene expression, epigenetic	0.04666	4
GO: Biological Process	GO:0006188	IMP biosynthetic process	0.002104	3
GO: Biological Process	GO:0046040	IMP metabolic process	0.004236	3
GO: Biological Process	GO:0002227	innate immune response in mucosa	0.01842	3
GO: Biological Process	GO:0006735	NADH regeneration	0.02186	3
GO: Biological Process	GO:0061718	glucose catabolic process to pyruvate	0.02186	3
GO: Biological Process	GO:0061621	canonical glycolysis	0.02186	3
GO: Biological Process	GO:0061620	glycolytic process through glucose-6-phosphate	0.02795	3
GO: Biological Process	GO:0061615	glycolytic process through fructose-6-phosphate	0.02998	3
GO: Biological Process	GO:0009303	rRNA transcription	0.03643	3
GO: Biological Process	GO:0006007	glucose catabolic process	0.03643	3
GO: Biological Process	GO:0002385	mucosal immune response	0.04234	3
GO: Biological Process	GO:0002251	organ or tissue specific immune response	0.04666	3
GO: Biological Process	GO:0006189	'de novo' IMP biosynthetic process	0.02231	2
GO: Biological Process	GO:0003360	brainstem development	0.02871	2
GO: Biological Process	GO:1904869	regulation of protein localization to Cajal body	0.03541	2
GO: Biological Process	GO:1904871	positive regulation of protein localization to Cajal body	0.03541	2

GO: Biological Process	GO:1904867	protein localization to Cajal body	0.04069	2
GO: Biological Process	GO:1990173	protein localization to nucleoplasm	0.04069	2
GO: Biological Process	GO:1904851	positive regulation of establishment of protein localization to telomere	0.04069	2
GO: Biological Process	GO:1903405	protein localization to nuclear body	0.04069	2
GO: Biological Process	GO:0070203	regulation of establishment of protein localization to telomere	0.04666	2
GO: Biological Process	GO:2000109	regulation of macrophage apoptotic process	0.04666	2
**Cellular Components**				
GO: Cellular Component	GO:1990904	ribonucleoprotein complex	3.911E-16	32
GO: Cellular Component	GO:0005739	mitochondrion	0.0001714	27
GO: Cellular Component	GO:0044430	cytoskeletal part	0.003519	21
GO: Cellular Component	GO:0005730	nucleolus	0.0001863	18
GO: Cellular Component	GO:0044427	chromosomal part	0.0002774	17
GO: Cellular Component	GO:0005694	chromosome	0.0009335	17
GO: Cellular Component	GO:0044429	mitochondrial part	0.001384	17
GO: Cellular Component	GO:0005840	ribosome	2.109E-10	16
GO: Cellular Component	GO:0031967	organelle envelope	0.01457	16
GO: Cellular Component	GO:0031975	envelope	0.01465	16
GO: Cellular Component	GO:0000785	chromatin	0.00001594	15
GO: Cellular Component	GO:0043005	neuron projection	0.03225	15
GO: Cellular Component	GO:0005759	mitochondrial matrix	0.00001598	14
GO: Cellular Component	GO:0019866	organelle inner membrane	0.0002047	14
GO: Cellular Component	GO:0015630	microtubule cytoskeleton	0.04078	14
GO: Cellular Component	GO:0044391	ribosomal subunit	7.008E-09	13
GO: Cellular Component	GO:0005743	mitochondrial inner membrane	0.0002774	13
GO: Cellular Component	GO:0031966	mitochondrial membrane	0.003519	13
GO: Cellular Component	GO:0005740	mitochondrial envelope	0.005569	13
GO: Cellular Component	GO:0044454	nuclear chromosome part	0.001374	12
GO: Cellular Component	GO:0000228	nuclear chromosome	0.002361	12
GO: Cellular Component	GO:0099513	polymeric cytoskeletal fiber	0.02534	11
GO: Cellular Component	GO:0099512	supramolecular fiber	0.02701	11

GO: Cellular Component	GO:0044445	cytosolic part	0.00007139	10
GO: Cellular Component	GO:0044297	cell body	0.04252	10
GO: Cellular Component	GO:0000313	organellar ribosome	9.626E-08	9
GO: Cellular Component	GO:0005761	mitochondrial ribosome	9.626E-08	9
GO: Cellular Component	GO:0032993	protein-DNA complex	0.00007139	9
GO: Cellular Component	GO:0043209	myelin sheath	0.0001339	9
GO: Cellular Component	GO:0000790	nuclear chromatin	0.002163	9
GO: Cellular Component	GO:0005925	focal adhesion	0.00662	9
GO: Cellular Component	GO:0005924	cell-substrate adherens junction	0.007063	9
GO: Cellular Component	GO:0030055	cell-substrate junction	0.007355	9
GO: Cellular Component	GO:0015629	actin cytoskeleton	0.0165	9
GO: Cellular Component	GO:0005912	adherens junction	0.01813	9
GO: Cellular Component	GO:0070161	anchoring junction	0.02262	9
GO: Cellular Component	GO:0015935	small ribosomal subunit	0.00001594	7
GO: Cellular Component	GO:0000786	nucleosome	0.0001277	7
GO: Cellular Component	GO:0044815	DNA packaging complex	0.0001499	7
GO: Cellular Component	GO:0005681	spliceosomal complex	0.001321	7
GO: Cellular Component	GO:0016604	nuclear body	0.03586	7
GO: Cellular Component	GO:0005884	actin filament	0.0004147	6
GO: Cellular Component	GO:0015934	large ribosomal subunit	0.0007423	6
GO: Cellular Component	GO:0035770	ribonucleoprotein granule	0.003519	6
GO: Cellular Component	GO:0030017	sarcomere	0.01059	6
GO: Cellular Component	GO:0044449	contractile fiber part	0.01457	6
GO: Cellular Component	GO:0030016	myofibril	0.01521	6
GO: Cellular Component	GO:0043292	contractile fiber	0.01932	6
GO: Cellular Component	GO:0005938	cell cortex	0.03218	6
GO: Cellular Component	GO:0005819	spindle	0.04835	6
GO: Cellular Component	GO:0000788	nuclear nucleosome	0.0001499	5
GO: Cellular Component	GO:0071013	catalytic step 2 spliceosome	0.0026	5
GO: Cellular Component	GO:0030863	cortical cytoskeleton	0.003519	5

GO: Cellular Component	GO:0022626	cytosolic ribosome	0.007355	5
GO: Cellular Component	GO:0044448	cell cortex part	0.01199	5
GO: Cellular Component	GO:0036464	cytoplasmic ribonucleoprotein granule	0.0141	5
GO: Cellular Component	GO:0030027	lamellipodium	0.03218	5
GO: Cellular Component	GO:0005763	mitochondrial small ribosomal subunit	0.0004147	4
GO: Cellular Component	GO:0000314	organellar small ribosomal subunit	0.0004147	4
GO: Cellular Component	GO:0005762	mitochondrial large ribosomal subunit	0.001084	4
GO: Cellular Component	GO:0000315	organellar large ribosomal subunit	0.001084	4
GO: Cellular Component	GO:0032432	actin filament bundle	0.008518	4
GO: Cellular Component	GO:0042641	actomyosin	0.01008	4
GO: Cellular Component	GO:0005903	brush border	0.03861	4
GO: Cellular Component	GO:0044798	nuclear transcription factor complex	0.04109	4
GO: Cellular Component	GO:0031674	I band	0.04145	4
GO: Cellular Component	GO:0002102	podosome	0.005147	3
GO: Cellular Component	GO:0005684	U2-type spliceosomal complex	0.007516	3
GO: Cellular Component	GO:0022627	cytosolic small ribosomal subunit	0.01558	3
GO: Cellular Component	GO:0097525	spliceosomal snRNP complex	0.02534	3
GO: Cellular Component	GO:0030532	small nuclear ribonucleoprotein complex	0.03218	3
GO: Cellular Component	GO:0001725	stress fiber	0.03851	3
GO: Cellular Component	GO:0005811	lipid droplet	0.03851	3
GO: Cellular Component	GO:0097517	contractile actin filament bundle	0.03851	3
GO: Cellular Component	GO:0044452	nucleolar part	0.04081	3
GO: Cellular Component	GO:0000932	P-body	0.04204	3
GO: Cellular Component	GO:0035985	senescence-associated heterochromatin focus	0.002571	2
GO: Cellular Component	GO:0031595	nuclear proteasome complex	0.008395	2
GO: Cellular Component	GO:0005832	chaperonin-containing T-complex	0.01008	2
GO: Cellular Component	GO:0002199	zona pellucida receptor complex	0.0141	2
GO: Cellular Component	GO:0031597	cytosolic proteasome complex	0.01521	2
GO: Cellular Component	GO:0008540	proteasome regulatory particle, base subcomplex	0.01521	2
GO: Cellular Component	GO:0005686	U2 snRNP	0.03218	2

GO: Cellular Component	GO:0005838	proteasome regulatory particle	0.03586	2
GO: Cellular Component	GO:0071011	precatalytic spliceosome	0.03968	2
GO: Cellular Component	GO:0035145	exon-exon junction complex	0.03968	2
GO: Cellular Component	GO:0022624	proteasome accessory complex	0.04109	2
GO: Cellular Component	GO:0005689	U12-type spliceosomal complex	0.04584	2
GO: Cellular Component	GO:0031941	filamentous actin	0.04835	2
GO: Cellular Component	GO:0071665	gamma-catenin-TCF7L2 complex	0.03218	1
GO: Cellular Component	GO:0042643	actomyosin, actin portion	0.03218	1
GO: Cellular Component	GO:0097226	sperm mitochondrial sheath	0.04922	1

**Decreased in the presence of SMURF2CG**				
**Molecular Functions**				
GO: Molecular Function	GO:0070011	peptidase activity, acting on L-amino acid peptides	0.01614	4
GO: Molecular Function	GO:0008233	peptidase activity	0.01614	4
GO: Molecular Function	GO:0034987	immunoglobulin receptor binding	0.0001714	3
GO: Molecular Function	GO:0003823	antigen binding	0.007026	3
GO: Molecular Function	GO:0004252	serine-type endopeptidase activity	0.01614	3
GO: Molecular Function	GO:0008236	serine-type peptidase activity	0.01614	3
GO: Molecular Function	GO:0017171	serine hydrolase activity	0.01614	3
GO: Molecular Function	GO:0004175	endopeptidase activity	0.02829	3
GO: Molecular Function	GO:0050421	nitrite reductase (NO-forming) activity	0.01614	1
GO: Molecular Function	GO:0098809	nitrite reductase activity	0.01614	1
GO: Molecular Function	GO:0004124	cysteine synthase activity	0.01614	1
GO: Molecular Function	GO:0070025	carbon monoxide binding	0.01614	1
GO: Molecular Function	GO:0004122	cystathionine beta-synthase activity	0.01614	1
GO: Molecular Function	GO:1904047	S-adenosyl-L-methionine binding	0.01614	1
GO: Molecular Function	GO:0070026	nitric oxide binding	0.01898	1
GO: Molecular Function	GO:0004739	pyruvate dehydrogenase (acetyl-transferring) activity	0.01898	1
GO: Molecular Function	GO:0001069	regulatory region RNA binding	0.01898	1

GO: Molecular Function	GO:1990715	mRNA CDS binding	0.02248	1
GO: Molecular Function	GO:0017018	myosin phosphatase activity	0.02248	1
GO: Molecular Function	GO:1990254	keratin filament binding	0.02661	1
GO: Molecular Function	GO:1990247	N6-methyladenosine-containing RNA binding	0.02829	1
GO: Molecular Function	GO:0032050	clathrin heavy chain binding	0.02829	1
GO: Molecular Function	GO:0004738	pyruvate dehydrogenase activity	0.02829	1
GO: Molecular Function	GO:0034604	pyruvate dehydrogenase (NAD+) activity	0.02829	1
GO: Molecular Function	GO:0034603	pyruvate dehydrogenase [NAD(P)+] activity	0.02829	1
GO: Molecular Function	GO:0097157	pre-mRNA intronic binding	0.02993	1
GO: Molecular Function	GO:0001849	complement component C1q binding	0.02993	1
GO: Molecular Function	GO:0016662	oxidoreductase activity, acting on other nitrogenous compounds as donors, cytochrome as acceptor	0.03134	1
GO: Molecular Function	GO:0016624	oxidoreductase activity, acting on the aldehyde or oxo group of donors, disulfide as acceptor	0.03134	1
GO: Molecular Function	GO:0043047	single-stranded telomeric DNA binding	0.03365	1
GO: Molecular Function	GO:0016661	oxidoreductase activity, acting on other nitrogenous compounds as donors	0.03668	1
GO: Molecular Function	GO:0098847	sequence-specific single stranded DNA binding	0.03668	1
GO: Molecular Function	GO:0048407	platelet-derived growth factor binding	0.03668	1
GO: Molecular Function	GO:0001846	opsonin binding	0.0415	1
GO: Molecular Function	GO:0019215	intermediate filament binding	0.04604	1
GO: Molecular Function	GO:0070006	metalloaminopeptidase activity	0.04626	1
GO: Molecular Function	GO:0035198	miRNA binding	0.04626	1
**Biological Processes**				
GO: Biological Process	GO:0006897	endocytosis	0.008702	5
GO: Biological Process	GO:0050776	regulation of immune response	0.01856	5
GO: Biological Process	GO:0006958	complement activation, classical pathway	0.0001185	4
GO: Biological Process	GO:0002455	humoral immune response mediated by circulating immunoglobulin	0.000146	4
GO: Biological Process	GO:0006956	complement activation	0.000146	4
GO: Biological Process	GO:0072376	protein activation cascade	0.0003019	4
GO: Biological Process	GO:0016064	immunoglobulin mediated immune response	0.0005791	4
GO: Biological Process	GO:0019724	B cell mediated immunity	0.0005791	4

GO: Biological Process	GO:0006959	humoral immune response	0.002642	4
GO: Biological Process	GO:0006909	phagocytosis	0.003907	4
GO: Biological Process	GO:0002449	lymphocyte mediated immunity	0.004195	4
GO: Biological Process	GO:0002460	adaptive immune response based on somatic recombination of immune receptors built from immunoglobulin superfamily domains	0.004195	4
GO: Biological Process	GO:0002443	leukocyte mediated immunity	0.009493	4
GO: Biological Process	GO:0002250	adaptive immune response	0.01302	4
GO: Biological Process	GO:0002253	activation of immune response	0.02173	4
GO: Biological Process	GO:0050778	positive regulation of immune response	0.03369	4
GO: Biological Process	GO:0006518	peptide metabolic process	0.04377	4
GO: Biological Process	GO:0002252	immune effector process	0.04377	4
GO: Biological Process	GO:0042742	defense response to bacterium	0.02785	3
GO: Biological Process	GO:0006898	receptor-mediated endocytosis	0.03574	3
GO: Biological Process	GO:0002429	immune response-activating cell surface receptor signaling pathway	0.0373	3
GO: Biological Process	GO:0070482	response to oxygen levels	0.0373	3
GO: Biological Process	GO:0006417	regulation of translation	0.0373	3
GO: Biological Process	GO:0002768	immune response-regulating cell surface receptor signaling pathway	0.03898	3
GO: Biological Process	GO:0034248	regulation of cellular amide metabolic process	0.03898	3
GO: Biological Process	GO:0060351	cartilage development involved in endochondral bone morphogenesis	0.01302	2
GO: Biological Process	GO:0036075	replacement ossification	0.01304	2
GO: Biological Process	GO:0001958	endochondral ossification	0.01304	2
GO: Biological Process	GO:0006910	phagocytosis, recognition	0.01753	2
GO: Biological Process	GO:0060325	face morphogenesis	0.01831	2
GO: Biological Process	GO:0030049	muscle filament sliding	0.01831	2
GO: Biological Process	GO:0033275	actin-myosin filament sliding	0.01831	2
GO: Biological Process	GO:0060323	head morphogenesis	0.02006	2
GO: Biological Process	GO:0060350	endochondral bone morphogenesis	0.02526	2
GO: Biological Process	GO:0006911	phagocytosis, engulfment	0.02526	2
GO: Biological Process	GO:0016441	posttranscriptional gene silencing	0.02526	2
GO: Biological Process	GO:0060324	face development	0.02526	2

GO: Biological Process	GO:0035194	posttranscriptional gene silencing by RNA	0.02526	2
GO: Biological Process	GO:0010171	body morphogenesis	0.02612	2
GO: Biological Process	GO:0050853	B cell receptor signaling pathway	0.02785	2
GO: Biological Process	GO:0010324	membrane invagination	0.02785	2
GO: Biological Process	GO:0002433	immune response-regulating cell surface receptor signaling pathway involved in phagocytosis	0.0373	2
GO: Biological Process	GO:0038096	Fc-gamma receptor signaling pathway involved in phagocytosis	0.0373	2
GO: Biological Process	GO:0060349	bone morphogenesis	0.0373	2
GO: Biological Process	GO:0050871	positive regulation of B cell activation	0.0373	2
GO: Biological Process	GO:0038094	Fc-gamma receptor signaling pathway	0.03813	2
GO: Biological Process	GO:0002431	Fc receptor mediated stimulatory signaling pathway	0.03896	2
GO: Biological Process	GO:0070252	actin-mediated cell contraction	0.03898	2
GO: Biological Process	GO:0033273	response to vitamin	0.04043	2
GO: Biological Process	GO:0050684	regulation of mRNA processing	0.04124	2
GO: Biological Process	GO:0030048	actin filament-based movement	0.04755	2
GO: Biological Process	GO:0043200	response to amino acid	0.04895	2
GO: Biological Process	GO:1903311	regulation of mRNA metabolic process	0.04895	2
GO: Biological Process	GO:1990428	miRNA transport	0.02421	1
GO: Biological Process	GO:1902618	cellular response to fluoride	0.02421	1
GO: Biological Process	GO:0043418	homocysteine catabolic process	0.03359	1
GO: Biological Process	GO:0006535	cysteine biosynthetic process from serine	0.03359	1
GO: Biological Process	GO:0042262	DNA protection	0.0373	1
GO: Biological Process	GO:0006565	L-serine catabolic process	0.0373	1
GO: Biological Process	GO:0019343	cysteine biosynthetic process via cystathionine	0.0373	1
GO: Biological Process	GO:0071306	cellular response to vitamin E	0.0373	1
GO: Biological Process	GO:0044691	tooth eruption	0.0373	1
GO: Biological Process	GO:0036135	Schwann cell migration	0.03898	1
GO: Biological Process	GO:1902617	response to fluoride	0.03898	1
GO: Biological Process	GO:0009093	cysteine catabolic process	0.03898	1
GO: Biological Process	GO:0019344	cysteine biosynthetic process	0.03898	1

GO: Biological Process	GO:0019448	L-cysteine catabolic process	0.03898	1
GO: Biological Process	GO:0046439	L-cysteine metabolic process	0.03898	1
GO: Biological Process	GO:1900147	regulation of Schwann cell migration	0.03898	1
GO: Biological Process	GO:0070814	hydrogen sulfide biosynthetic process	0.04377	1
GO: Biological Process	GO:0019346	transsulfuration	0.04377	1
GO: Biological Process	GO:0009092	homoserine metabolic process	0.04377	1
GO: Biological Process	GO:0036072	direct ossification	0.04895	1
GO: Biological Process	GO:0001957	intramembranous ossification	0.04895	1
**Cellular Components**				
GO: Cellular Component	GO:0005615	extracellular space	0.036	5
GO: Cellular Component	GO:0072562	blood microparticle	0.0001646	4
GO: Cellular Component	GO:0042571	immunoglobulin complex, circulating	0.00007241	3
GO: Cellular Component	GO:0019814	immunoglobulin complex	0.00007241	3
GO: Cellular Component	GO:0036464	cytoplasmic ribonucleoprotein granule	0.04429	2
GO: Cellular Component	GO:0035770	ribonucleoprotein granule	0.0443	2
GO: Cellular Component	GO:0005584	collagen type I trimer	0.03328	1
GO: Cellular Component	GO:0071756	pentameric IgM immunoglobulin complex	0.03328	1
GO: Cellular Component	GO:0071754	IgM immunoglobulin complex, circulating	0.03328	1
GO: Cellular Component	GO:0071753	IgM immunoglobulin complex	0.03328	1
GO: Cellular Component	GO:0071749	polymeric IgA immunoglobulin complex	0.03328	1
GO: Cellular Component	GO:0071751	secretory IgA immunoglobulin complex	0.03328	1
GO: Cellular Component	GO:0071748	monomeric IgA immunoglobulin complex	0.03328	1
GO: Cellular Component	GO:0071745	IgA immunoglobulin complex	0.03328	1
GO: Cellular Component	GO:0071746	IgA immunoglobulin complex, circulating	0.03328	1
GO: Cellular Component	GO:0045098	type III intermediate filament	0.036	1
GO: Cellular Component	GO:0071598	neuronal ribonucleoprotein granule	0.0399	1
GO: Cellular Component	GO:0045254	pyruvate dehydrogenase complex	0.0443	1
GO: Cellular Component	GO:0071439	clathrin complex	0.0472	1
